# The *Welwitschia* genome reveals a unique biology underpinning extreme longevity in deserts

**DOI:** 10.1038/s41467-021-24528-4

**Published:** 2021-07-12

**Authors:** Tao Wan, Zhiming Liu, Ilia J. Leitch, Haiping Xin, Gillian Maggs-Kölling, Yanbing Gong, Zhen Li, Eugene Marais, Yiying Liao, Can Dai, Fan Liu, Qijia Wu, Chi Song, Yadong Zhou, Weichang Huang, Kai Jiang, Qi Wang, Yong Yang, Zhixiang Zhong, Ming Yang, Xue Yan, Guangwan Hu, Chen Hou, Yingjuan Su, Shixiu Feng, Ji Yang, Jijun Yan, Jinfang Chu, Fan Chen, Jinhua Ran, Xiaoquan Wang, Yves Van de Peer, Andrew R. Leitch, Qingfeng Wang

**Affiliations:** 1 Core Botanical Gardens/Wuhan Botanical Garden, Chinese Academy of Sciences, Wuhan, China; 2 Sino-Africa Joint Research Centre, Chinese Academy of Sciences, Wuhan, China; 3 Key Laboratory of Southern Subtropical Plant Diversity, FairyLake Botanical Garden, Shenzhen & Chinese Academy of Sciences, Shenzhen, China; 4 Royal Botanic Gardens, Kew, Surrey, UK; 5 Gobabeb Research and Training Centre, Walvis Bay, Namibia; 6 State Key Laboratory of Hybrid Rice, College of Life Sciences, Wuhan University, Wuhan, China; 7 Department of Plant Biotechnology and Bioinformatics, Ghent University, Ghent, Belgium; 8 Center for Plant Systems Biology, VIB, Ghent, Belgium; 9 School of Resources and Environmental Science, Hubei University, Wuhan, China; 10 Key Laboratory of Aquatic Botany and Watershed Ecology, Wuhan Botanical Garden, Chinese Academy of Sciences, Wuhan, China; 11 Seqhealth Technology, Wuhan, China; 12 Institute of Chinese Materia Medica, China Academy of Chinese Medical Sciences, Beijing, China; 13 Shanghai Key Laboratory of Plant Functional Genomics and Resources, Shanghai Chenshan Botanical Garden, Shanghai, China; 14 College of Biology and Environment, Nanjing Forestry University, Nanjing, China; 15 Guangdong Provincial Key Laboratory of Silviculture, Protection and Utilization, Guangdong Academy of Forestry, Guangzhou, Guangdong China; 16 School of Life Sciences, Sun Yat-Sen University, Guangzhou, China; 17 Education Key Laboratory for Biodiversity Science and Ecological Engineering, Fudan University, Shanghai, China; 18 Institute of Genetics and Developmental Biology, Chinese Academy of Sciences, Beijing, China; 19 State Key Laboratory of Systematic and Evolutionary Botany, Institute of Botany, Chinese Academy of Sciences, Beijing, China; 20 Centre for Microbial Ecology and Genomics, Department of Biochemistry, Genetics and Microbiology, University of Pretoria, Hatfield, South Africa; 21 College of Horticulture, Academy for Advanced Interdisciplinary Studies, Nanjing Agricultural University, Nanjing, China; 22 School of Biological and Chemical Sciences, Queen Mary University of London, London, UK

**Keywords:** Evolutionary ecology, Evolutionary genetics, Transcriptomics, Abiotic

## Abstract

The gymnosperm *Welwitschia mirabilis* belongs to the ancient, enigmatic gnetophyte lineage. It is a unique desert plant with extreme longevity and two ever-elongating leaves. We present a chromosome-level assembly of its genome (6.8 Gb/1 C) together with methylome and transcriptome data to explore its astonishing biology. We also present a refined, high-quality assembly of *Gnetum montanum* to enhance our understanding of gnetophyte genome evolution. The *Welwitschia* genome has been shaped by a lineage-specific ancient, whole genome duplication (~86 million years ago) and more recently (1-2 million years) by bursts of retrotransposon activity. High levels of cytosine methylation (particularly at CHH motifs) are associated with retrotransposons, whilst long-term deamination has resulted in an exceptionally GC-poor genome. Changes in copy number and/or expression of gene families and transcription factors (e.g. *R2R3MYB*, *SAUR*) controlling cell growth, differentiation and metabolism underpin the plant’s longevity and tolerance to temperature, nutrient and water stress.

## Abstract (web summary)

*Welwitschia mirabilis* is a unique plant that only has two leaves, but it can survive in hostile conditions of the African desert. Here, the authors report its chromosome-level genome assembly and discuss how gene function and regulation have given rise to its unique morphology and environmental adaptions.

## Introduction

**Fig. 1:**
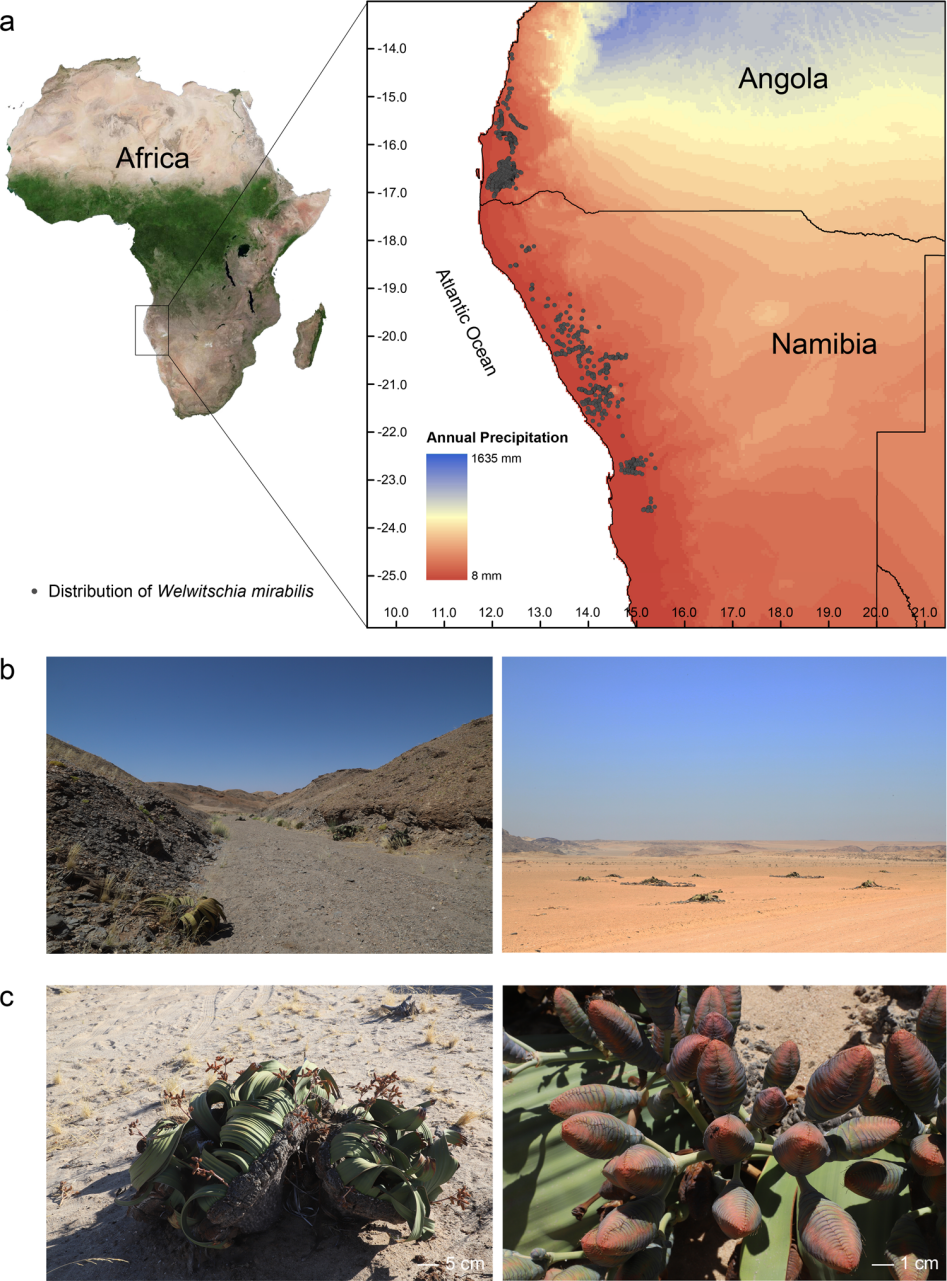
Geographical location and typical habitat of *Welwitschia mirabilis*. **a** The annual precipitation of the area in Africa where the species grows measured using the Koeppen-Geiger index. The distribution of *W. mirabilis* (gray dot) is based on occurrence records available from the Global Biodiversity Information Facility (GBIF) and new data from Professor N. Jürgens (pers. comm.). **b** Two typical niches, an arid valley, and coastal desert. **c** Male (left) and female individuals (right) in cone.

Joseph Dalton Hooker, when the director of Kew Gardens, UK (1865–1885) is reported to have said of *Welwitschia* that “it is out of the question the most wonderful plant ever brought to this country and one of the ugliest”. The species shows remarkable tenacity in surviving in the Kaokoveld Centre of Africa, an arid coastal desert of northern Namibia and southern Angola, with annual precipitation of <50 mm^1^ (Fig. 1, Supplementary Note 1). The species has a highly distinctive morphology, consisting of just two leaves that grow continuously throughout the plant’s life. This can last several thousand years, resulting in the longest-lived leaves in the plant kingdom^2–4^. Ever since its first formal description in 1863^5^, the biological curiosities of *Welwitschia* have been the subject of extensive discussion, including between Charles Darwin, Asa Gray, and Hooker^6^.

*Welwitschia* is the only species of the plant family Welwitschiaceae although recent molecular data suggest there are two genetically and geographically distinct populations that may correspond to sub-species^1^. The species is within Gnetophyta, an ancient gymnosperm lineage that includes only two other genera, *Gnetum* (family Gnetaceae) and *Ephedra* (family Ephedraceae). Most phylogenetic analyses reveal that gnetophytes are monophyletic, with *Welwitschia* and *Gnetum* forming a clade that is sister to *Ephedra*^7^. The divergence of *Welwitschia* and *Gnetum* is likely to have been over 110 million years ago (mya), given a welwitschioid fossil seedling, *Cratonia cotyledon*, found in early Cretaceous beds of Brazil^8^. The relationship of gnetophytes to other gymnosperms and angiosperms has caused much speculation due to their conflicting phylogenetic placement^9,10^, unique morphological features^11^, and the extinction of critical seed plant groups^12^. Nevertheless, the current consensus, based predominantly on gene sequences, is that gnetophytes are more closely related to conifers (the “*Gnepine*”, “*Gnecup*” or “*Gnetifer*” hypotheses) than to other gymnosperms^7^.

In this work, we use genome assembly data together with extensive epigenomic and transcriptomic data to unveil a distinctive genome structure that enhances our understanding of genome evolution in gnetophytes and sheds light on gene families that have given rise to *Welwitschia*’s unique morphology, extreme longevity, and its ability to survive in harsh, arid environments.

## Results

### Genome sequencing and annotation of *Welwitschia*

Here, we report a high-quality chromosome-level sequence assembly of *Welwitschia* (Supplementary Table 1). To better understand the evolution of *Welwitschia*’s adaptations, we also provide the first high-quality chromosomal level genome assembly of *Gnetum montanum* (hereafter *Gnetum*), building upon previous analyses^13^ (Supplementary Table 1, 2). We combined Oxford Nanopore (108×) and Illumina (134×) sequencing to generate a genome assembly of *Welwitschia*, comprising 6.86 gigabases (Gb) and covering 98% of the estimated genome size (7.0 Gb/1C)^14^. Contig and scaffold N50 lengths were 1.48 Mb and 295 Mb, respectively (Supplementary Table 1). For *Gnetum*, 10× Genomics and BioNano Genomics platforms were used to increase the length of scaffolds of a previous *Gnetum* assembly^13^. Optical and chromosome-contact (HiC) maps for both *Welwitschia* and *Gnetum* were then produced and scaffolds were anchored and ordered to generate 21 and 22 pseudo-chromosomes for *Welwitschia*, and *Gnetum*, respectively (Supplementary Fig. 1). The pseudo-chromosomes represent 93.65% (6.43 Gb) of the total assembly length of *Welwitschia* and 86.47% (3.57 Gb) of *Gnetum* (Supplementary Table 1).

The pseudo-chromosomes of *Welwitschia* revealed that the longest chromosome was ~551.97 Mb and 3.3-fold longer than the shortest chromosome (Supplementary Table 3). These results agree with previous cytogenetic observations showing the karyotype of *Welwitschia* to comprise telocentric chromosomes differing considerably in total length^15^. A total of 26,990 protein-coding genes were predicted of which 89.11% were validated by transcript evidence gathered from RNA sequencing of multiple tissues and/or by orthology with genes in other species (Supplementary Fig. 2a, b). BUSCO (Benchmarking Universal Single-Copy Orthologs) analysis suggests that 83.47% of the genes had been recovered. For *Gnetum*, the improved assembly shows a considerable enhancement over the previous release, with scaffold N50 lengths of 157.93 Mb, and identifying 27,354 genes, recovering 84.6% of BUSCO genes (Supplementary Fig. 2c, Supplementary Table 1).

### Genome evolution and dynamics

**Fig. 2:**
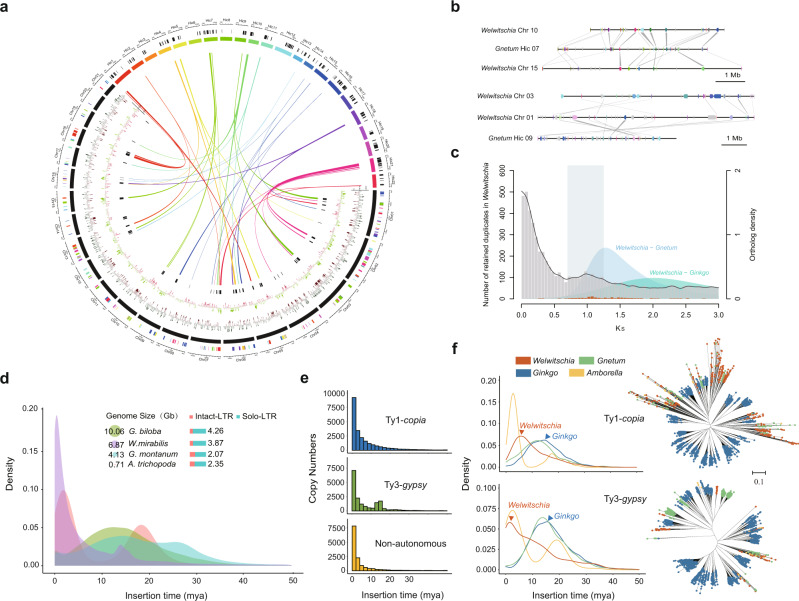
Genome evolutionary history of *Welwitschia*. **a** A circos plot showing the inter-genomic collinearity analysis between the *Welwitschia* and *Gnetum* genomes. From outside to inside, each track shows (1) one-to-one orthologous collinear regions between the two genomes with colors showing their orthologs on different chromosomes in the other species; (2) the chromosomes of *Welwitschia* in black and *Gnetum* in colors; (3) the coverage of tandem repeats in a 5 Mbp window with green and red bars showing above or below the mean coverage of tandem repeats across the genome for *Welwitschia* (standard deviation in gray), respectively; (4) the coverage of long terminal repeats (LTRs) in a 5 Mbp window with light green and light red bars showing above or below the mean coverage of tandem repeats across the genome (standard deviation in gray), respectively; and (5) paralogous collinear regions in the *Welwitschia* genome with at least five anchor pairs. The bands denote collinear regions where one region in *Gnetum* corresponds to two orthologous regions in *Welwitschia*. **b** Two examples illustrating one segment in *Gnetum* corresponding to two paralogous segments in *Welwitschia*. Genes in light gray are non-homologs. Genes with the same colors are homologs and homologous genes are connected with dark gray bands when the two segments are alongside. **c** Distributions of synonymous substitutions per synonymous site (*K*_S_) for the whole paranome of *Welwitschia* (light gray histogram and dark gray line). The *y* axis on the left shows the number of retained duplicates and there is a small peak at *K*_S_ of 1, which represents a WGD event. The y axis on the right shows the orthologue density between the two species. For one-to-one orthologs between *Welwitschia* – *Gnetum* and *Welwitschia* – *Ginkgo* (color-filled curves of kernel-density estimates) the peaks represent species divergence events and *K*_S_ values correspond with the degree of orthologue divergence. The *K*_S_ values for anchor pairs from collinear regions are indicated in orange (left *y* axis). The pale gray rectangle highlights the *K*_S_ regions in which the paralogous genes were used for absolute dating. **d** Estimation of LTR activity showing a recent burst in *Welwitschia* less than two million years ago (mya). **e** In this recent burst of activity, the majority of elements responsible were both autonomous (13,893 copies of Ty1-*copia* and 9,999 copies of Ty3-*gypsy*) and non-autonomous LTRs (10,589 copies), with peaks of activity <5 mya. **f** Heuristic neighbor-joining trees constructed from 3,298 full-length Ty3-*gypsy* and 2,224 of Ty1-*copia* sequences from *Welwitschia*, *Gnetum*, *Ginkgo,* and *Amborella*. There were many more complete elements identified in *Ginkgo* (4,237) than in the other species, elements that were likely to have been derived from a peak of activity about 15 mya. A few clades of *Welwitschia*-derived Ty3-*gypsy* (131 copies) probably arose from the recent activity at ~2 mya (see **e**). Source data underlying Fig. d–f are provided as a Source Data file.

The distribution of synonymous substitutions per synonymous site (*K*_S_) for all paralogous genes in the genomes of *Welwitschia* and *Gnetum*, as well as for paralogous genes in collinear or syntenic regions, suggests an ancient whole-genome duplication (WGD) event for *Welwitschia*, but not *Gnetum* (Fig. 2a, b, Supplementary Fig. 3). In *Welwitschia*, there is a signature peak of duplicate genes with a *K*_S_ value close to 1 (Fig. 2c), as previously observed in *K*_S_-based age analyses using transcriptome data^13,16^, whilst this peak is absent in *Gnetum*. The *Gnetum* genome is also devoid of intra-genomic collinear regions, while for *Welwitschia* we detected 198 pairs of paralogous genes in 47 such duplicated regions in the genome (Supplementary Fig. 3). Inter-genomic comparisons between both gnetophyte genomes further identified 21 genomic segments in the *Gnetum* genome, each corresponding to two orthologous segments in the *Welwitschia* genome (Fig. 2b), again supporting an ancient WGD event unique to *Welwitschia*.

Absolute dating of the WGD in *Welwitschia* suggests that the WGD event occurred ~86 mya with a 90% confidence interval (CI) giving a range of 78–96 mya (Supplementary Fig. 4). Interestingly, although *Welwitschia* and *Gnetum* have a similar number of chromosomes; 21 and 22, respectively, collinear regions from a single chromosome in *Gnetum* often found their orthologs distributed on several chromosomes in *Welwitschia* (Fig. 2a), suggesting substantial genomic rearrangements after the lineages diverged. Extensive reshuffling of genes and/or genomic regions in the *Welwitschia* genome might also explain why so few duplicated regions can be detected, as identifying collinear regions as a signal for the occurrence of a WGD requires conservation in gene order. In support of this, when considering synteny by which paralogous genes are retained but gene collinearity has been lost^17^, we found an additional 773 paralogous genes located in 222 syntenic regions, giving further strong support to the WGD in *Welwitschia* (Supplementary Fig. 3).

The majority (86.85%) of the genome of *Welwitschia* consists of repetitive elements that are distributed over all chromosomes, with no indication from the density of repeats to indicate where centromeric regions lie (Supplementary Fig. 1, Supplementary Table 3). In addition, there is no indication of subtelomeric tandem repeats in *Welwitschia*, although they do occur in *Gnetum* (Supplementary Fig. 1a, b). The most abundant repeats in *Welwitschia* are long terminal repeat-retrotransposons (LTR-RTs), which comprise 55.26% of the genome (Supplementary Table 4). Estimates of sequence divergence times between adjacent 5′ and 3′ LTRs of the same retrotransposon suggest that there was a burst of LTR-RT activity within the last 1–2 mya (Fig. 2d), dominated by both autonomous and non-autonomous LTRs^18^ (Fig. 2e, Supplementary Table 5). Recent bursts of non-autonomous elements have been observed in high-quality genome assemblies of two angiosperm species (*Camellia sinensis*^18^ and *Oryza* species^19^) and may be a phenomenon that becomes more commonly observed as genome assembly qualities improve. Potentially, retrotransposition of non-autonomous elements inhibits the retrotransposition frequency of complete elements, through competition for the proteins needed for amplification that are encoded by autonomous elements^18,19^, hence explaining the high frequency of non-autonomous elements in *Welwitschia*.

Phylogenetic analysis of reverse transcriptase (RT) genes from complete retrotransposons (Ty3-*gypsy* and Ty1-*copia* elements, containing all expected protein-coding domains) in *Welwitschia*, *Gnetum*, *Amborella trichopoda* (hereafter, *Amborella*), and *Ginkgo biloba* (hereafter, *Ginkgo*) revealed deep, ancient diverging clades containing sequences from *Welwitschia*, *Gnetum,* and sometimes also *Amborella*, but excluding sequences from *Ginkgo* (Fig. 2f, Supplementary Table 6). Our previous work comparing full-length Ty3-*gypsy* and Ty1-*copia* elements in *Gnetum* with *Pinus taeda*^13^, were similar to the comparisons between *Gnetum* and *Ginkgo*, in that most LTR clades were species-specific and had deep divergent histories, indicating the slow accumulation of ancient repeats independently in each lineage. These results contrast with the repeats from *Welwitschia*, *Gnetum* and *Amborella*, where multiple deeply diverging clades were not species-specific, but retained elements from all three species.

Our analyses failed to uncover evidence of numerous *Welwitschia*-specific clades (except perhaps some Ty3-*gypsy* clades, Fig. 2f). This pattern differs markedly from that observed in *Ginkgo*, where there are many *Gingko*-specific clades with fans of diverging repeats, probably derived from peaks of activity ~15 mya (Fig. 2d, f). There were also many more complete autonomous elements identified in *Ginkgo* than in the gnetophytes or *Amborella* (Supplementary Table 6).

The ratio of solo LTR/intact LTR was considerably higher in *Welwitschia* (i.e., 3.87; 4,610 solo-LTRs: 1,191 intact LTRs) compared with either *Gnetum* (2.07; 971: 470) or *Amborella* (2.35; 214: 91), whereas even higher ratios were observed in *Ginkgo* (4.26; 60,623: 14,128) (Fig. 2d). Solo-LTRs are thought to arise through excision-based DNA recombination, including between adjacent LTRs of the same element, leading to their removal and genome downsizing^20^. *Welwitschia*, despite having a lineage-specific WGD, has a relatively small genome for a gymnosperm, being ~1/3 the mean genome size of 421 gymnosperm species (i.e., 18 Gb/1 C)^21^. Perhaps, the higher frequency of solo-LTRs in *Welwitschia* compared with *Gnetum* reflects an elevated frequency of recombination-based removal of retroelements.

Overall, in the last two million years it appears that the *Welwitschia* genome has been impacted by the expansion of both autonomous and non-autonomous LTR repeats within a background of the ongoing reduction in all types of retroelements.

**Fig. 3:**
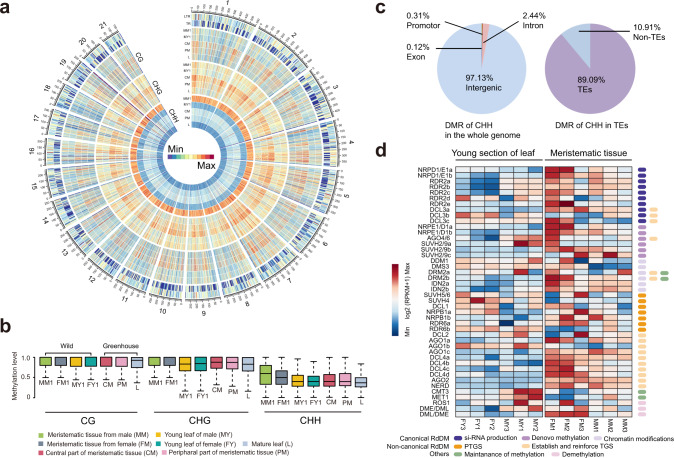
The DNA methylation landscape of *Welwitschia* and dynamic reprogramming between tissues mediated by the RNA-directed DNA methylation machinery. **a** The level of CG methylation varies >75%, whilst CHG and CHH methylation varies >25%, over at least five informative sequenced cytosines. Heat maps showing CG, CHG, and CHH methylation in the basal meristem of a male individual sampled in the wild (MM1), a young leaf from a male individual (the newly emergent region, MY1), and a male individual from the greenhouse, including the central region of the basal meristem (CM), the peripheral part of the basal meristem (PM) and leaf (L). Methylation levels were calculated and presented in 1 Mb windows, with the maximum set to be the highest value among the  five tissues in each 1 Mb window (maximum values were: 4,619 (methylated CG); 7,552 (methylated CHG), 56,186 (methylated CHH)). **b** Box plots showing the variation of CG, CHG, and CHH methylation among different tissues (each box shows the median methylation value (horizontal line) ± SD) and whiskers on vertical lines show minimum and maximum values). Data were analyzed from the total number of CG, CHG, CHH methylation sites from *n* = 7 biologically independent samples (MM1, FM1 (basal meristem of a female individual), MY1, FY1 (young leaf of a female individual), CM, PM, L). **c** Differentially methylated region (DMR) statistics of methylated cytosines at CHH trinucleotides in the genome. (left) The proportion of methylated cytosines that are differentially methylated between individuals in different genomic domains, and (right) the proportion of DMR at CHH trinucleotides occurring in transposable elements (TEs). **d** Most genes involved in canonical and non-canonical methylation mediated by the RdDM pathway *(y* axis) show increased transcript abundance (upregulated red, downregulated blue) in meristematic tissues compared with young leaves.

We compared the DNA methylome of two types of somatic tissue (basal meristem and young leaves) in *Welwitschia* (Supplementary Tables 7–9, Supplementary Note 2), studying both greenhouse material and material collected in the wild (see Plant Materials). The global methylation levels of cytosines in CG dinucleotide and CHG (H represents A, T, or C) trinucleotide sequence contexts were high in meristems and leaves, reaching on average 78.32% and 76.11% of all cytosines, respectively (Fig. 3a, b, Supplementary Data 1). These values are similar to those observed in the conifer *Picea abies*^22^ but considerably higher than is typically reported for angiosperms, where ~50% of cytosines are methylated on average^23^. The average methylation level of cytosines in the CHH context in both meristem and leaf tissue was 35.7%, which is considerably higher than previously reported for angiosperms and gymnosperms (Supplementary Data 1) and is perhaps the highest value for a plant to date. For example, an analysis of 34 angiosperms revealed that 85% of species had CHH methylation levels lower than 10%, with the highest value being 18.8% in *Beta vulgaris*^24^, whilst in the gymnosperm, *P. abies*, only ~1.5% of cytosines in CHH trinucleotides were methylated in cultured tissues^22^.

Despite the high average level of CHH methylation in *Welwitschia*, values varied considerably between tissues and contributed substantially to the occurrence of genome-wide differentially methylated regions (DMRs) (Fig. 3c, Supplementary Table 10). Of the regions that were differentially, CHH methylated between individuals, over 97% of the sites occurred within intergenic regions and 89% of these were within transposable elements (TEs) (Fig. 3c, Supplementary Table 11, Supplementary Note 3). Levels of methylated CHH were consistently lower in leaves (24%) than basal meristems, although in the latter there were substantial differences between wild-collected (58.72%), and glasshouse grown (31.42%) individuals (Fig. 3b, Supplementary Data 1).

An analysis of genes involved in the RNA-directed DNA methylation (RdDM) pathway^25,26^ showed upregulation of most genes in the basal meristem compared with leaves (Fig. 3d and Supplementary Data 2). In particular, we found upregulation of transcripts encoding proteins needed for small RNA (smRNA) biogenesis (e.g., *NRPD4* (a component of RNA Polymerase IV), *RDR2,* and *DCL3*). Furthermore, most genes directly involved in the deposition of methyl groups onto cytosine were upregulated in basal meristems (e.g., *DRM2*), as were key genes associated with the non-canonical RdDM pathway (e.g., *DCL2*, *DCL4*).

Because of the upregulation of genes involved in both canonical and non-canonical RdDM pathways, we assessed the levels of uniquely mapped reads of 21, 22, 23, and 24 nt smRNAs (Supplementary Table 12). We observed an increase in abundance of both 21 nt and 24 nt small interfering RNAs (siRNAs) in the basal meristem (Supplementary Fig. 5a), consistent with the higher levels of CHH methylation found in this tissue. Nevertheless, the majority of 21 and 24 nt siRNAs mapped to intergenic regions (up to 50%) and introns (~10%) (Supplementary Fig. 5b), regions that are rich in TEs (i.e., TEs comprised 84.98% of intergenic regions and 69.29% of introns) (Supplementary Table 13).

The hypermethylation of TEs in meristematic tissue is likely to have been reinforced by both canonical and non-canonical RdDM pathways due to the abundance and nature of 21 and 24 nt siRNA. Differential methylation of these elements reflects both developmental changes (i.e., leaves vs meristems) and environmental effects on basal meristems (i.e. glasshouse versus wild-sourced material). The latter may reflect responses to environmental stresses (light, temperature, water) experienced by the wild-collected plants growing in the Namibian desert. Several studies have shown that environmental factors, such as temperature, can induce “epigenetic memory”^27,28^. Beyond that, the reinforcement of TE silencing is crucial for the maintenance of genome integrity in stem cells and undifferentiated cells since these can develop into tissues such as reproductive organs. High levels of epigenetic silencing of TEs may also be an important, albeit costly response (in terms of nutrients and energy requirements of the epigenetic machinery of repeat silencing) to maintain meristem integrity in long-lived organisms.

Compared with other seed plants, the total GC content of *Welwitschia* is unusually low (~29.07%), with only one plant species with a lower value reported so far^29^ (the orchid *Calypso bulbosa*, 23.9%). Intergenic regions were particularly GC-poor in *Welwitschia* (Supplementary Fig. 6a). Such low levels were also observed in regions identified as being collinear with *Gnetum*, which are not so GC poor, suggesting that the nucleotide landscapes have changed considerably since the genera diverged (Supplementary Fig. 6b). GC-rich DNA provides more targets for methylation than GC-poor DNA^30,31^ and over time more opportunities for deamination of methylated cytosines toward thymine^32^. We found that TEs, including their protein-coding domains, had remarkably high levels of methylation, although their GC content was low (28.77%) (Supplementary Fig. 6c). Furthermore, incomplete LTR-RTs in *Welwitschia* were found to have even lower GC content (29.00%) than intact LTR-RTs (35.82%), whereas incomplete and intact LTR-RTs of *Gnetum* had similar levels (of 38.56% and 39.29%, respectively; Supplementary Fig 6d). The higher GC content in genes compared with other genomic domains could be a consequence of GC-biased gene conversion, which is reported to occur in recombination-rich regions of the genome^33^. Together, these results indicate that long-term deamination of methylated cytosines has occurred particularly in the intergenic regions of *Welwitschia*, reflected by the reduced GC content of TEs and incomplete LTR-RTs. Genomic DNA with high GC content is considered to be more thermostable^34^, yet incurs a higher biochemical cost compared with AT base synthesis^35^. It has been shown that nutrient limitation provides a strong selection pressure on nucleotide usage in prokaryotes^36^ and plants^37^ leading to a bias towards AT-rich genomes. Thus, it is possible that the long-term deamination of methylated cytosine residues, and a reduction in genome size after the ancestral WGD event, would have resulted in a more streamlined, water and nutrient-efficient genome (especially given the nutrient costs needed for high levels of methylation silencing, above) that is better adapted to harsh, nutrient- and water-limited conditions.

### The extreme longevity of two leaves

**Fig. 4:**
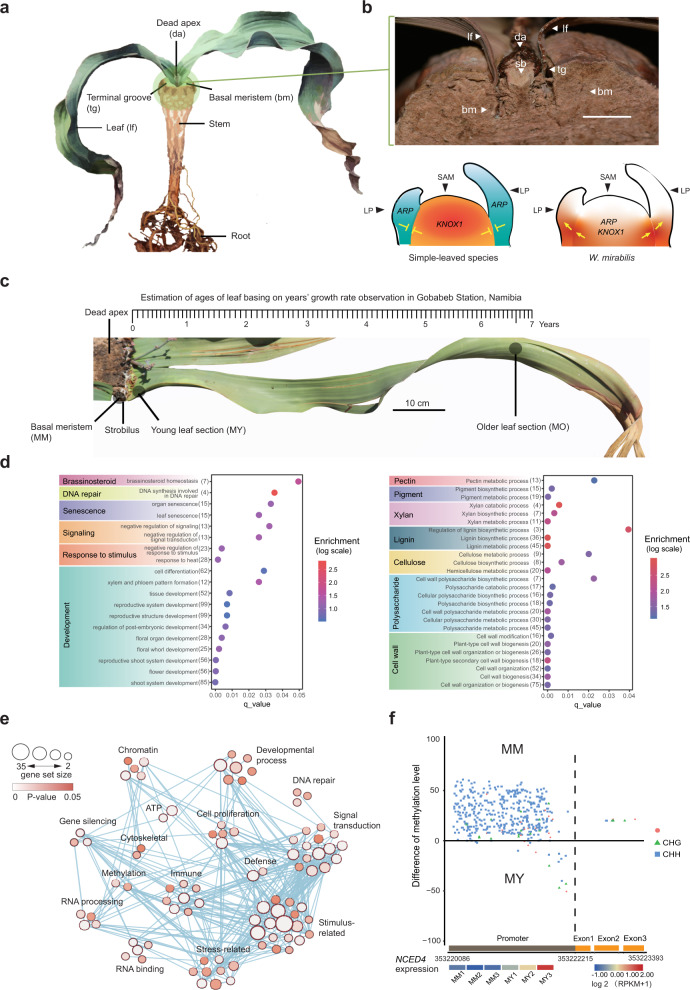
Transcriptome comparisons between meristematic and vegetative tissues. **a** Image showing key features of a young *Welwitschia* plant indicating the position of the basal meristem and the terminal groove from which the strap-like leaves emerge. **b** Longitudinal section of the apical region of a *Welwitschia* stem (upper): lf = leaf, bm = basal meristem, sb = origin of strobilus, tg = terminal groove, da = dead apex. Diagrammatic representation of *KNOTTED*-like homeobox Class 1 (*KNOX 1*) and ASYMMETRIC LEAVES1 /ROUGHSHEATH2 /PHANTASTICA (*ARP*) gene expression in a typical plant with determinate leaf growth (lower left) and in *Welwitschia* (lower right). With determinate leaf growth, there is an antagonistic regulation of *KNOX 1* and *ARP*, which does not occur in *Welwitschia*, resulting in indeterminate leaf growth (SAM = shoot apical meristem, LP = leaf primordia). **c** Position of tissues along the leaf axis that were sampled for transcriptome analysis, along with an estimation of the age for each leaf region sampled, based on growth rates observed during the long-term ecological monitoring of naturally growing plants at the Gobabeb Station, Namibia. **d** Significant upregulated expression of genes in basal meristems compared to young leaf sections (left). The number of genes associated with each category is indicated in brackets after the term description, and enrichment values are indicated by color, as shown in the color scale. GO term enrichment for genes highly expressed in young leaf sections compared with old leaf sections (right). **e** Weighted gene co-expression network analysis revealed expression differences between basal meristems and young leaf material (MM vs MY). The number of genes in each GO term enrichment category at each node (Fisher’s exact test with two-sided and false discovery rate correction used for calculating P values) are shown by the circle size. The probability that the node contains gene(s) that are controlling differential gene expression between tissues is depicted by node color. The lines connecting each node indicate correlated gene expression between genes. **f** The enzyme *NCED* (9-cis-epoxycarotenoid dioxygenase), called *NCED4* in *Welwitschia*, is regulated by differential CHH methylation. Hypomethylated cytosines at CHH trinucleotides in the promoter region of *NCED4* (negative values) in particular correlate with increased expression in young leaf sections (MY) compared with basal meristem tissues (MM).

Unlike other plants, the shoot apical meristem of *Welwitschia* dies in the young plant shortly after the appearance of true leaves and meristematic activity moves to the basal meristem. This meristem generates the two long-lived, highly fibrous, and strap-like leaves, which show indeterminate growth and emerge from two terminal grooves at the top of the stem like a conveyor belt^3,38–40^ (Fig. 4a, b, Supplementary Note 4).

Previous studies in *Welwitschia* proposed that *KNOTTED*-like homeobox Class 1 (*KNOX 1*) genes are expressed in the leaf base, causing a change in the mode of leaf growth from determinate to indeterminate^41^. Co-expression of ASYMMETRIC LEAVES1/ROUGHSHEATH2/ PHANTASTICA (*ARP*) and *KNOX 1* genes in the shoot apical meristem and leaf primordia in *Streptocarpus* have also been linked to the extended leaf basal meristem activity in the development of unequal cotyledons^42^. In this study, we observed overlapping gene expression of *ARP3*, *ARP4,* and *KNOX 1* in the “basal meristem” (Supplementary Fig. 7a, Supplementary Data 3), a situation that is not observed in most simple-leaved species (Fig. 4b). To search for further signatures of indeterminate leaf growth, we characterized gene activity in the basal meristem compared with leaves using GO enrichment and weighted gene co-expression network analyses (WGCNA) (Fig. 4d, e, Supplementary Fig. 8, Supplementary Data 4). One category of genes that was upregulated in the meristem belonged to the category “brassinosteroid homeostasis and metabolic process” (Fig. 4d). Brassinosteroids play an important role in driving meristem growth and cell proliferation^43–45^. We, therefore, investigated whether the upregulation of these genes was also associated with increased synthesis of brassinosteroids and observed, as expected, higher levels of castasterone in basal meristems compared with leaves (Supplementary Fig. 9). We also observed upregulation of genes belonging to the category “DNA synthesis involved in DNA repair” in the basal meristem (Fig. 4d), enabling us to identify the upregulation of specific genes involved in DNA repair and homologous recombination (Supplementary Fig. 7b, Supplementary Data 3). WGCNA show genes and pathways that are specifically co-expressed and revealed the coordinated expression of genes involved in “stress-related”, “stimulus-related” via enhancement of “signal transduction” (Fig. 4e). All these data are consistent with the ongoing meristematic activity required for the continuous, indeterminate growth of *Welwitschia* leaves in the environmentally stressful conditions experienced by the plants throughout their long lives.

To find genes that may have expanded in copy number in response to the unusual growth habit or to stress, we conducted a comprehensive characterization of expanded gene families in *Welwitschia* compared to other representative land plants (Supplementary Fig. 10, Supplementary Data 5, Supplementary Note 5). From these, we identified and further characterized genes in *Welwitschia* that had particularly increased in copy number and are known to be involved in stress responses. These included *R2R3-MYB* transcription factors belonging to subfamily VIII (a subgroup containing *AtMYB11* and its paralogs *AtMYB12*, *AtMYB111*), which are expanded in *Welwitschia* (11 copies) compared to other land plants (other species studied had no more than five copies, Supplementary Fig. 11). Subfamily VIII is the largest subfamily of MYB transcription factors^46^ and *R2R3-MYB* are extensively involved in plant development, secondary metabolism, cell proliferation, and stress responses^47,48^. We observed that both meristematic tissue and young leaf tissue had higher expression levels of these proliferated genes than old leaf sections (Supplementary Fig. 11b). In *Arabidopsis*, overexpression of *AtMYB11* is associated with reduced growth rate and reduced proliferation activity in meristem cells^49^. The expansion of *R2R3-MYB* genes might therefore be an adaptive response in *Welwitschia* for regulating cell division in the basal meristem to enable the slow and continuous growth, tissue development, and maturation over the long periods when environmental conditions are unfavorable.

Previous studies of long-lived or water/heat stress-adapted plants generally suggest that biotic and abiotic stress responses are positively selected for and play roles in the continuous arms race against the environment and pathogens^50–52^. Using GO enrichment to compare patterns of gene expression between young and older leaf sections separated by >6 years of growth^53^ (Fig. 4c, Supplementary Note 4), we observed significantly higher expression of genes in the young leaves involved in “pectin metabolic process”, “lignin biosynthesis”, “cellulose biosynthesis”, and “polysaccharide metabolic” (Fig. 4d). The upregulation of lignin biosynthesis pathway genes is associated with woody fibers laid down in early leaf development (Supplementary Data 4). In addition, subfamilies of the *SAUR* genes (small auxin upregulated RNA genes) involved in regulating cell elongation in plants^54^ were uniquely expanded in copy number in *Welwitschia* (Supplementary Note 6). Typically, *SAUR* genes occur in plant genomes in 60–140 copies^54^ whereas in *Welwitschia* there are specific expansions of gene members in two subfamilies (*SAUR17* and *SAUR43,58*) compared with six angiosperms, three gymnosperms, and one bryophyte species analyzed (Supplementary Fig. 12, Supplementary Data 5, 6). All of these genes are involved in the elongation and development of the highly fibrous strap-like leaves, acting to protect them from herbivory and shearing damage by wind and sandstorms.

Caseinolytic protease (*ClpP*) in plants has a role in maintaining functional proteins through the removal of misfolded, damaged, and short-lived proteins in plastids^55,56^. In *Arabidopsis thaliana* and *Oryza sativa*, Clp proteases are more abundant in younger leaves than older ones^57,58^, whereas some paralogues, like *Clp 3* and *Clp 5*, show higher expression in senescing *Arabidopsis* leaves^59^, perhaps associated with stress responses in these dying tissues. In contrast, there were no obvious differences in the expression of *Clp* genes in *Welwitschia* between young and old leaf sections (smallest *P* value> 0.21), with both tissues showing these genes were upregulated compared with basal meristems (Supplementary Fig. 7c, Supplementary Data 3). These proteins are likely to be important in the transition of proplastids to photosynthetically active chloroplasts in the young leaf, which is one of the most important metabolic processes in plant growth^58,60^. The expression of these proteins from the earliest emergence of the leaf to sections of the leaf 6 years later is likely to reflect the necessity to maintain protein homeostasis throughout the long life of the leaf, in the face of significant temperature and water stress.

Further studies to investigate how *Welwitschia* is able to survive in such hostile environments involved exploring the heat shock proteins (HSPs), which are known to protect other proteins from stress-induced misfolding, denaturation, and aggregation under both temperature and salt stress^61^. In *O. sativa* (rice), HSPs are induced by heat stress where they act to enable seed germination and root growth at high temperatures^62^. In *Welwitschia,* we identified several paralogues belonging to the HSP family *HSP-20* (subfamily CVI) that were amplified in the genome via tandem duplication (Supplementary Note 7). We also observed that they were upregulated in the basal meristem compared with leaf sections (Supplementary Fig. 13, Supplementary Data 3). It is likely that the meristematic tissue of *Welwitschia* holds the key to this plant’s extreme longevity, as well as the continuous growth of its elongated leaves. In wild populations, the main body of the plant can remain healthy even when the leaves are largely destroyed. However, once the meristematic tissue is damaged, the individual soon dies. Thus, it is reasonable to expect a higher expression of HSP in meristems than in leaves, since protecting this tissue from heat or water stress damage is essential.

Similar to the results for HSPs, a subfamily of basic helix-loop-helix (*bHLH*) transcription factors, responsible for survival under water deprivation, was also specifically expanded in *Welwitschia*^63^ (Supplementary Data 5). All these data indicate an adaptative response to coping with abiotic stress conditions, including extremely high temperatures and wide daily fluctuations in temperatures. We observed that nucleotide-binding site–leucine-rich repeat protein genes, which play a role in the biotic stress responses, were expanded in copy number in *Welwitschia* compared with other gnetophytes and herbaceous angiosperms^64^ (Supplementary Data 5), but is similar to that observed in other long-lived gymnosperm and angiosperm trees^51,65^, indicating adaptations related to the long lifespan of these plants^51^.

Abscisic acid (ABA) accumulates as a response to environmental stress in plants^66^. ABA is generated by the cleavage of carotenoids and the metabolic process leading to ABA synthesis requires *NCED* (9-cis-epoxycarotenoid dioxygenase), which converts 9-cis-neoxanthin to xanthoxin and then xanthoxin dehydrogenase (*ABA2*) converts xanthoxin to abscisic aldehyde (Supplementary Fig. 14a). Multiple paralogues of both these genes in *Welwitschia* showed differential expression between basal meristems and young leaf sections (Supplementary Fig. 14b, c). It is known that the activity of *NCED* is a rate-limiting step in ABA synthesis (Supplementary Fig. 14a) and that *NCED* transcripts accumulate before ABA builds up in drought-stressed plants^67^. We observed that tandemly amplified paralogues of *NCED4* genes were particularly upregulated in basal meristems of *Welwitschia* (Supplementary Fig. 14b, c). To further confirm this, the ABA concentration was quantified in meristematic tissue, as well as in young and old sections of leaves. As expected, ABA concentrations reflected *NCED4* gene expression (Supplementary Fig. 14a, Supplementary Data 7). Interestingly, one *NCED4* gene showed hypomethylation of CHH sites in the promoter regions of young leaves compared with basal meristems (Fig. 4f, Supplementary Note 8). In the lettuce, *Lactuca sativa*, the promoter of *NCED4* is reported to play a role in sensing and responding to heat and is necessary to inhibit seed germination at high temperatures^68^. Thus, it is likely that differential CHH methylation in the *NCED4* promoter in *Welwitschia* is an adaptation to control the transcriptional activity of downstream genes^69,70^. It will be informative to fully demonstrate a link between *NCED* expression, epigenetic controls, and ABA synthesis in protecting *Welwitschia* against heat stress. We expect that ABA functions by limiting growth in the earliest developmental stages of the leaf, but once the fibrous tissues are fully developed, this regulation becomes less important for the long-term functioning of the leaf, and the genes become silenced.

## Discussion

*Welwitschia*-like fossils suggest that the *Welwitschia* lineage existed in diverse morphological forms in northern Gondwana during the Early Cretaceous^71^. The species’ current distribution has been arid or semi-arid for ~55–80 million years^1,72^ and, due to the influence of the Benguela Upwelling System, aridity has become increasingly intense over the last 10 million years, leading to the most severe aridity today^73,74^. Increasing aridity may have triggered a cascade of events now visible in the *Welwitschia* genome, such as the burst of LTR-RTs within the last 1–2 million years since these elements are known to be activated by environmental stress^75^.

Because LTR-RTs are both metabolically demanding and potentially damaging to gene activity, an adaptive response may have been to increase genome-wide cytosine methylation to silence their activity, giving rise to the high levels now seen across the genome. Damage caused by TEs or environmental stress such as ultraviolet radiation could have contributed to a high frequency of chromosomal rearrangements and the low levels of synteny between *Welwitschia* and *Gnetum*. DNA that has been damaged and faithfully repaired by homologous recombination can also be marked by methylation^76,77^. High levels of cytosine methylation over millions of years would, in turn, have increased the frequency of deamination of methylated cytosines towards thymine^31,78^, leading to *Welwitschia’*s GC-poor genome. Interestingly, a GC-poor genome may also confer selective advantages under the nutrient stress of *Welwitschia*’s environment, as observed in other plants and bacteria^36,37^, because GC dinucleotides are less N demanding than AT dinucleotides.

An ancient WGD, ~86 mya, coupled with the genome dynamics associated with a high frequency of LTR-RT removal has led to genome downsizing since the last WGD event. This contrasts with other gymnosperms that are predicted to be slowly increasing in genome size^20,79^. It is likely that under nutrient and water stress there has been selected for a smaller genome, which acts to reduce the nutrient requirements of the cell (through fewer nucleic acids and nuclear proteins^80^) and to enhance water use efficiency (through increased stomatal responsiveness of smaller cells^81^).

*Welwitschia* is famous for its longevity. Carbon-14 dating of some of the largest plants has shown that some individuals are over 1,500 years old^2^. Photographs documenting little change in the size of two medium-sized plants over 90 years nevertheless reveal how slowly these plants are growing^82^ (~10–13 cm per year throughout the lifespan of the plant^53^). *Welwitschia* gets its Afrikaans name “tweeblaarkanniedood”, meaning “two leaves that cannot die”, because of the activities of *KNOX1*, *ARP3,* and *ARP4* and other genes typical of meristem activity that are continually expressed, including genes in a subfamily of *R2R3-MYB* transcription factors that are likely to regulate cell growth and differentiation. The expansion in copy number of *HSP20* and *bHLH* gene family members, as well as upregulation of *NCED4,* are all associated with adaptation for efficient metabolism under environmental stress, functioning to prevent the basal meristem and young portions of the leaves from dying during the long periods of adverse conditions.

The genome now provides a benchmark from which further comparative studies will be possible to enhance our understanding of the adaptations that have enabled extreme longevity in harsh and arid environments.

## Methods

### Plant materials

For genome sequencing, we selected an ex situ conserved individual of *Welwitschia* (male) growing in Fairy Lake Botanical Garden (FLBG) (plant accession: SZBG 00052740). We also collected a large range of tissue samples of *Welwitschia* (male cones, root, and leaves) from the same plant for RNA sequencing (RNA-seq) for helping with assembly and assessment. High molecular weight genomic DNA was isolated using the Qiagen DNeasy Plant Mini Kit (Qiagen, USA). Total RNA was isolated using TRIzol (Invitrogen) and further treated with RNase-free DNase I (Promega, USA).

For RNA sequencing for the functional analyses, we sequenced three biological replicates of independent samples of tissues from three plants, the tissues being (i) meristematic tissue of male individuals (samples MM1, MM2, MM3), (ii) meristematic tissue of female individuals (samples FM1, FM2, FM3), (iii) young section (region indicated in Fig. 4c) of the leaf of male individuals (samples MY1, MY2, MY3), (iv) young section of a leaf of female individuals (samples FY1, FY2, FY3), (v) old section of a leaf of male individuals (samples MO1, MO2, MO3), and (vi) old section of a leaf of female individuals (samples FO1, FO2, FO3). These wild-collected samples were collected in “Welwitschia Wash” (S23.6124; E 15.1696), in the Namib-Naukluft Park, and from the northern bank of the Kuiseb River. In addition, three tissue types from a single individual grown in a greenhouse at the FLBG (plant accession: SZBG 00052750) were also collected which comprised (i) the central part of the meristematic tissue (CM), (ii) the peripheral part of the meristematic tissue (PM), and (iii) the mature leaf (L). Summary information for the source of the DNA-, and RNA-seq data for each tissue is given in Supplementary Table 7.

### De novo sequencing and genome assembly

After extracting high molecular weight DNA (>15 kb), the large size fraction was selected by automated gel electrophoresis (BluePippin). Then, the DNA was treated with the End-repair/dA tailing module (New England Biolabs, Inc.). After purification, adapter ligation was performed using a ligation sequencing kit (LSK109, Oxford Nanopore Technologies). Finally, the DNA library was quantified by Qubit. Sequencing data were generated on an Oxford Nanopore GridION, and reads with quality scores of <7 were discarded. Reads passing this quality threshold were corrected using Nextdenovo (version 1.1, with parameters read_cuoff = 1k; seed_cutoff = 15k). The preliminary genome was assembled with WTDBG^83^ (version 1.2.8, with parameters -k 0 -p 19 -S 2 -E 2–rescue-low-cov-edges–aln-noskip). To increase the accuracy of the assembly, the preliminary genome was polished iteratively three times with Illumina short reads by using Nextpolish^84^ with parameters sgs_options = -max_depth 100 -bwa. Genome heterozygosity was estimated by mapping Illumina short reads to the polished version of the genome using Burrow-Wheeler Aligner for short-read alignment (https://github.com/lh3/bwa, http://arxiv.org/abs/1303.3997). The mapping rate was 99%. Alignments were followed by SNP calling with samtools^85^ (https://github.com/samtools). The heterozygosity rate was estimated with bcftools (https://github.com/samtools/bcftools, 10.1093/gigascience/giab008).

To anchor hybrid scaffolds onto chromosomes, genomic DNA was extracted from the leaves of one *Welwitschia* individual to construct a HiC library. We obtained sequencing data using an Illumina Novaseq platform (Illumina, San Diego, CA). First, adapter sequences of raw reads were trimmed, and low-quality paired-end reads were removed using fastp (version 0.12.6). Then, the remaining paired-end reads were aligned to the assembled scaffolds using Bowtie2 (version 2.3.2, with parameters -end-to-end–very-sensitive -L 30). “Valid” paired-end reads (i.e. unique mapped paired-end reads) were identified and retained using HiC-Pro (version 2.8.1 with parameter -v -S -t 100 -m 100000000 -s 100 -l 700 -a) for further analysis. Invalid read pairs, including dangling-end, self-cycle, re-ligation, and dumped products were filtered using HiC-Pro (v2.8.1) (https://github.com/nservant/HiC-Pro, 10.1186/s13059-015-0831-x). The scaffolds were clustered, ordered, and oriented onto chromosomes using LACHESIS (https://github.com/shendurelab/LACHESIS, 10.1038/nbt.2727) with parameters CLUSTER_MIN_RE_SITES = 100, CLUSTER_MAX_LINK_DENSITY = 2.5, CLUSTER NONINFORMATIVE RATIO = 1.4, ORDER MIN N RES IN TRUNK = 60, ORDER MIN N RES IN SHREDS = 60). Finally, placement and orientation errors exhibiting obvious discrete chromatin interaction patterns were manually adjusted. We also used HiC to anchor the scaffolds of *Gnetum* onto chromosomes following the protocol above. The statistics of *Welwitschia* and *Gnetum* genome assemblies are given in Supplementary Table 1.

### Annotation

The chromosome-level assembly of the *Welwitschia* genome was annotated using the following steps: for repeat annotation of the *Welwitschia* genome, both similarity-based predictions and de novo approaches were adopted. Specifically, repeats from the de novo approach were detected by RepeatModeler (version open-1.0.11, http://repeatmasker.org/RepeatModeler/, with parameters -engine ncbi), LTR-FINDER^86^ (version 1.07, https://github.com/xzhub/LTR_Finder/find/master/, with parameters -C -w 2, 10.1093/nar/gkm286), PILER^87^ (version 1.0, http://www.drive5.com/piler/, with default parameters) and RepeatScout^88^ (version 1.0.5, http://bix.ucsd.edu/repeatscout/, with default parameters). Both RepeatMasker (version open-4.0.7, http://www.repeatmasker.org/RepeatMasker/, with parameters -a -nolow -no_is -norna) and RepeatProteinMask (version open-4.0.7, http://www.repeatmasker.org/RepeatMasker/, with parameters -noLowSimple -*p* value 0.0001 -engine wublast) were used to scan the assembled *Welwitschia* genome based on similarity to known repeats in the library of Repbase Update (20170127). In addition, we also used the program Tandem Repeats Finder^89^ (version 4.09, http://tandem.bu.edu/trf/trf.html, with the parameters “2 7 7 80 10 50 2000 -d –h”) to search for tandem repeats.

### Gene prediction

A de novo-based, homology-based, and RNA-seq-based gene prediction approach was used to identify protein-coding genes in the *Welwitschia* genome assembly. Augustus^90^ (version 3.3.1, http://bioinf.uni-greifswald.de/augustus/, with default parameters), SNAP^91^ (version 2006-07-28, http://korflab.ucdavis.edu/, with default parameters) and Genscan^92^ (version 1.0, http://hollywood.mit.edu/burgelab/software.html, with default parameters) were used for the de novo-based gene prediction. Genome sequences and gff files of fourteen species (*Gnetum montanum*, *Pinus taeda*, *Ginkgo biloba*, *Amborella trichopoda*, *Selaginella moellendorffii*, *Physcomitrella patens*, *Azolla filiculoides*, *Salvinia cucullata, Oryza sativa*, *Arabidopsis thaliana*, *Apostasia shenzhenica*, *Vitis vinifera*, *Populus trichocarpa,* and *Solanum lycopersicum*) were used for homology-based prediction using GeMoMa^93^ (version 1.5.3, www.jstacs.de/index.php/GeMoMa, with default parameters). RNA-seq reads from tissues (male cones, root, and leaves) were aligned back to the genome allowing for gapped or spliced alignments of reads using TopHat^94^ (version 2.0.13, http://ccb.jhu.edu/software/tophat, with parameters–max-intron-length 500000 -m 2–library-type fr-unstranded) and Cufflinks (version 2.1.1, http://cufflinks.cbcb.umd.edu/manual.html, with parameters -I 500000 -p 1–library-type fr-unstranded -L CUFF). PASA^95^ (version 2.0.2) was used for the RNA-seq-based method of gene prediction. Finally, the results from the three approaches were integrated using EVidenceModeler^96^ (EVM; version 1.1.1). Genes with TEs were identified and removed from the final gene set by using TransposonPSI software (http://transposonpsi.sourceforge.net/). For gene function annotation, predicted protein-coding genes were annotated using two strategies. (i) First, predicted protein sequences were aligned to SwissProt protein database (http://www.gpmaw.com/html/swiss-prot.html) using Blastp under the best match parameters. The gene pathways of predicted sequences were extracted from the KEGG Automatic Annotation Server (version 2.1, https://www.genome.jp/kaas-bin/kaas_main, with default parameters). (ii) The annotation of motifs and domains was performed using InterProScan^97^ (version 5.32-71.0, http://www.ebi.ac.uk/interpro/) to search against the open database InterPro which includes the databases Pfam, ProDom, PRINTS, PANTHER, SMRT, and PROSITE^97^. These two approaches were combined to form the final dataset.

### Detection of WGD events

*K*_S_-based paralog age distributions were constructed as previously described^98^. In brief, the paranome was constructed by performing an all-against-all protein sequence similarity search using BLASTP with an *E* value cutoff of 1 × 10^−10^. Next, gene families were built with the mclblastline pipeline (v.10-201) (micans.org/mcl)^99^. Each gene family was aligned using MUSCLE^100^ (version 3.8.31), and *K*_S_ estimates for all pairwise comparisons within a gene family were obtained through maximum likelihood estimation using the CODEML^101^ program of the PAML^102^ package (version 4.4c). Gene families were then subdivided into subfamilies for which *K*_S_ estimates between members did not exceed a value of 5. Phylogenetic trees were constructed for each subfamily using PhyML^103^ under default settings, to correct for redundancy of *K*_S_ values (a gene family of *n* members produces *n*(*n*–1)/2 pairwise *K*_S_ estimates for *n*–1 retained duplication events). For each duplication node in the resulting phylogenetic tree, all *m K*_S_ estimates between the two daughter clades were added to the *K*_S_ distribution with a weight of 1/*m* (where *m* is the number of *K*_S_ estimates for a duplication event) so that the weights of all *K*_S_ estimates for a single duplication event summed to one.

Paralogous gene pairs found in duplicated collinear and syntenic segments (anchor pairs) from *Welwitschia*, were detected using i-ADHoRe^104,105^ (version 3.0) with “level_2_only=TRUE” and “cluster_type=hybrid”. The latter parameter enables i-ADHoRe to detect both duplicated collinear and syntenic segments, where anchor pairs are retained with regard and without regard to gene order, respectively. The identified anchor pairs are assumed to correspond to the most recent WGD event (Supplementary Fig. 17). Likewise, the collinear and syntenic segments between *Gnetum* and *Welwitschia* were identified and are shown in Supplementary Fig. 17.

We also performed a pairwise collinear analysis between *Welwitschia* and *Gnetum*. Homologous sequences between the two species were identified using all-against-all BLASTP (*E* value <1 × 10^−5^). Weak matches identified by applying a *c*-score of 0.5 (indicating their BLASTP bit-scores were below 50% of the bit-scores of the best matches)^106^ were removed. Then i-ADHoRe 3.0 was used to identify collinear segments with parameters as described above except using “level_2_only = FALSE”, “cluster_type=collinear”, and “anchor_points=5”, allowing i-ADHoRe to detect collinear regions with more than two segments within and between the two genomes. Identified collinear segments were then visualized by the R packages *circlize*^107^ (Fig. 2a) and *genoPlotR*^108^ (Fig. 2b).

*K*_S_-based ortholog age distributions were constructed by identifying one-to-one orthologs between species using reciprocal best hits^109^, followed by *K*_S_ estimation using the CODEML program as above. To compare different substitution rates in gnetophytes, *K*_S_ distributions for one-to-one orthologs between *Ginkgo* and each of *Welwitschia*, *Gnetum*, and *Ephedra*, as well as one-to-one orthologs between *Ephedra* and each of *Welwitschia* and *Gnetum*, were compared to confirm *Welwitschia* and *Gnetum* have similar substitution rates (Supplementary Fig. 18). Then, we compared *K*_S_ distributions for one-to-one orthologs between *Welwitschia* and each of *Gnetum* and *Ginkgo* with the *K*_S_ distributions of the whole paranome and anchor pairs in *Welwitschia* to locate the WGD (Fig. 2c).

Absolute dating of the identified WGD event in *Welwitschia* was performed as previously described^110^. In brief, paralogous gene pairs located in duplicated segments (anchor pairs) and duplicated pairs lying under the WGD peak (peak-based duplicates) were collected for phylogenetic dating. We selected anchor pairs and peak-based duplicates present under the *Welwitschia* WGD peak and with *K*_S_ values between 0.7 and 1.25 (gray-shaded area in Fig. 2c) for absolute dating. An orthogroup was created for each WGD paralogous pair that included the two paralogs plus several orthologs from other plant species as identified by InParanoid^111^ (version 4.1) using a representative ortholog from the order Rosales, one from the Fabales, one from the Malpighiales, two from the Malvales, one from the Solanales, two from the Poaceae (Poales), one from either *Musa acuminata*^112^ (Zingiberales) or *Phoenix dactylifera* (Arecales), and one ortholog from *Gnetum*^13^. In total, eight orthogroups based on anchor pairs and 131 orthogroups based on peak-based duplicates were collected. The node joining the two *Welwitschia* WGD paralogs was then dated using BEAST^113^ (version 1.7) under an uncorrelated relaxed clock model and an LG + G (four rate categories) evolutionary model. A starting tree with branch lengths satisfying all fossil prior constraints was created according to the consensus APGIV phylogeny^114^. Fossil calibrations were implemented using log-normal calibration priors on the following nodes: the node uniting the Malvidae based on the fossil *Dressiantha bicarpellata*^115^ with prior offset = 82.8, mean = 3.8528, and SD = 0.5^116^; the node uniting the Fabidae based on the fossil *Paleoclusia chevalieri*^117^ with prior offset = 82.8, mean = 3.9314, and SD = 0.5^118^; the node uniting the monocots based on the fossil *Spirematospermum chandlerae*^119^ with prior offset = 83.5, mean = 3.7910, and SD = 0.5^120^; the node uniting the eudicots and monocots based on the sudden abundant appearance of eudicot tricolpate pollen in the fossil record^121^ with prior offset = 124, mean = 4.0786 and SD = 0.5^110^ (see below); the node uniting *Gnetum* and *Welwitschia* based on the fossil *Cratonia cotyledon*^122^ with prior offset = 110, mean = 3.0226 and SD = 0.5^123^; and the root with prior offset = 307, mean = 3.8876, and SD = 0.5^124^. The offsets of these calibrations represent hard minimum boundaries, and their means represent locations for their respective peak mass probabilities. A run without data were performed to ensure proper placements of the marginal calibration priors^125^. A run without data indicated that the distribution of the marginal calibration prior for the node uniting eudicots and monocots did not correspond to the specified calibration density, so we reduced the mean in the calibration prior of the root with offset = 124, mean = 3.5081, SD = 0.5 to locate the marginal calibration prior at 170 Ma^110^. The Markov chain Monte Carlo (MCMC) for each orthogroup was run for 10 million generations with sampling every 1000 generations, resulting in a sample size of 10,000. The resulting trace files of all orthogroups were evaluated manually using Tracer^113^ (version 1.5) with a burn-in of 1000 samples to ensure proper convergence (minimum effective sampling size for all statistics was at least 200). In total, 126 orthogroups selected from both anchor pairs or peak-based duplicates were accepted, and absolute age-estimates of the node uniting the WGD paralogous pairs were grouped into one absolute age distribution (Supplementary Fig. 14, for which kernel-density estimation and a bootstrapping procedure were used to find the peak consensus WGD age estimate and its 90% CI boundaries, respectively (Supplementary Fig. 4). More detailed methods are available in Vanneste *et al*.^110^. In addition, we built a second set of orthogroups for each WGD paralogous pair by removing the orthologs from *Gnetum* in the taxonomic sampling listed above, leading to a separate set of 25 orthogroups based on anchor pairs and 380 orthogroups based on peak-based duplicates. All the fossil calibrations as described above were used except the node uniting *Gnetum* and *Welwitschia*. The MCMC was carried out and 352 orthogroups were accepted and further analyzed as described above, resulting in an alternative estimate of the *Welwitschia* WGD in ~111–122 Ma (Supplementary Fig. 19), earlier than the estimate based on the orthogroups with orthologs from *Gnetum*.

### Identification of LTR-RTs

Genome sequences of two gnetophytes (*Gnetum* and *Welwitschia*), *Ginkgo,* and an angiosperm *Amborella* were chosen to perform a comparative analysis of LTR-RTs. LTR-FINDER^86^ (version 1.07, https://github.com/xzhub/LTR_Finder/find/master/, with parameters -w 2 -d 0 -l 100) was used for the de novo detection of LTR-RTs.

### Estimation of insertion time of the LTR-RTs

The 5′-LTR is usually identical to the 3′-LTR at the time when a retrotransposon is inserted into the genome. All the LTRs sequences identified with complete 5′-LTR and 3′-LTR were used. Each of the 5′-LTR flanking sequences and 3′-flanking sequences was aligned by MUSCLE^100^ (version 3.8.31, http://www.drive5.com/muscle, with default parameters) and the distance of the alignment sequences was calculated by the disMat (EMBOSS: version 6.6.0.0, http://emboss.sourceforge.net/, with parameters -nucmethod 2). The insertion time was calculated using the following formula: *T* = *K*/2*r* (divergence between LTRs/substitution per site per year). The mutation rate (per base per year) used for *Amborella *was 1.8 × 10^−8^ and 2.2 × 10^−9^ for *Ginkgo*, *Gnetum,* and *Welwitschia*^79^.

### Analyses of the RT genes of complete retrotransposons

Proteins of the “Cores Seq” RefSeqdatabase in *Gypsy* Database 2.0 (GyDB^126^) were aligned against the LTR-RT sequences of *Amborella, Ginkgo, Gnetum,* and *Welwitschia* using blastall^127^ (version 2.2.26, ftp://ftp.ncbi.nlm.nih.gov/blast/executables/blast+/, with parameters -p tblastn -e 1e-05 -F T -m 8). Each blast hit was linked by Solar^128^ (version 0.9.6). GENEWISE^129^ (version 2.4.1, https://www.ebi.ac.uk/~birney/wise2/, with default parameters) was used to predict the candidate gene structure based on the homogenous alignments. Then the RT sequence regions were extracted from the protein sequences and only the longest RT sequences with >140 aa (amino acid) and <2 stop codons for each LTR-RT were retained for the phylogenetic analysis. This comprised making multiple sequence alignments using MUSCLE^100^ (version 3.8.31, http://www.drive5.com/muscle, with default parameters). Subsequently, the phylogenetic trees of Ty1-*copia*-like and Ty3-*gypsy*-like LTR-RTs were constructed using Neighbor-Joining (NJ) in TreeBeST^130^ (version 1.9.2, http://treesoft.sourceforge.net/treebest.shtml, parameters: -t mm –b 1).

The LTR-RTs containing alignments with the domains were classed into five types. “Ty1-*copia*” with domains “INT-RT-RNaseH” or “RNaseH-RT-INT”, “Ty3-*gypsy*” with domains “RT-RNaseH-INT” or “INT-RNaseH-RT”, “Retroviridae” with domains “ENV”, “Incompleted Ty1-*copia* or Ty3-*gypsy*“ contains part domains of “INT”, “RT”, “RNaseH”. Those with no domains were defined as non-autonomous^126^.

### Definition and detection of solo-LTRs and intact LTRs

All initial LTR-RTs detected from LTR-FINDER were blasted against the “Cores Seq” RefSeqdatabase in *Gypsy* Database 2.0 (GyDB^126^) using blastall^127^ (version 2.2.26, ftp://ftp.ncbi.nlm.nih.gov/blast/executables/blast+/, with parameters -m 8 -a 4 -F F -v 500 -b 250 -e 1e-5). Each blast hit was linked by Solar^128^ (version 0.9.6). Alignments, where both the coverage and identity were >30%, were retained. Those LTR-RTs containing alignments with the domains of “GAG” (Capsid protein), “AP” (Aspartic proteinase), “INT” (Integrase), “RT”, and “RH” (RNaseH) were considered to be intact LTR-RTs^131^. Using the LTR sequences (5’LTR or 3’LTR) from intact LTR-RTs, a nucleotide BLAST search was performed against the genome to find potential solo-LTRs. An in-house Perl script was written for filtering out the following types of false solo-LTRs: (a) LTRs which overlapped with truncated LTR-RTs; (b) LTRs located within 5 kb of the scaffold edge; (c) LTRs with <0.7 coverage and <0.7 identity cutoff; (d) LTRs identified within 500 bp either side of a gap sequence in the assemblies. For the detection of truncated LTR-RTs, all LTR-RT sequences reported from LTR-FINDER were blasted against the genomes, and alignments with coverage >80% and identity >60% were considered to correspond to the presence of truncated LTR-RTs.

### Methylome and transcriptome sequencing

Total DNAs were extracted using the Hi-DNAsecure Plant Kit (Tiangen Biotech Co., Ltd. Beijing, Cat. No. DP350) following the manufacturer’s instructions. The integrity of DNA was visualized using electrophoresis on a 0.8% (w/v) agarose gel. The purity of DNA was determined by examining the A260/A280 ratio using a Nanodrop^TM^ OneC spectrophotometer (Thermo Fisher Scientific Inc.). DNA was quantified using a Qubit3.0 with the Qubit^TM^ DNA broad range assay kit (Life Technologies). DNA was sonicated in a sonicator (JY92-IIN, Xinzhi, Ningbo, Zhejiang, China), to give a fragment size ranging from 200 to 500 bp, purified with Ampure^TM^ XP beads (Beckman Coulter). Bisulfite conversion was conducted on 1 μg fragmented DNA using the EZ DNA Methylation-Gold Kit (Zymo Research, Cat. No. D5005). A total of 5 ng of lambda phage DNA was added to determine the efficiency of C-U conversion of unmethylated Cs. The conversion was carried out using the EZ DNA Methylation-Gold™ Kit (Zymo Research, Cat. No. D5005). Sequencing libraries were prepared using the Accel-NGS^®^ Methyl-Seq DNA Library kit (Swift Biosciences^TM^, Cat. No. 30024). PCR products corresponding to 300-500 bp were enriched, quantified, and finally sequenced on a HiSeq X-10 sequencer (Illumina).

For RNA sequencing (RNA-seq), the sample collection information for different tissue types is given in Supplementary Table 6. Total RNA was isolated using TRIzol reagent (Invitrogen) and then further treated with RNase-free DNase I (Promega, USA). All RNA-seq libraries were constructed using the NEB Next Ultra^TM^ RNA Library Prep Kit (NEB, USA) and sequenced using the NovaSeq 6000 Illumina platform.

Following bisulphite sequencing, raw reads were first cleaned with SOAPnuke^132^ (version 2.0.5) to remove residual adaptor sequences and reads with low-quality scores. Cleaned reads were mapped to the reference genome and duplicated reads were removed using Bismark^133^ (version 0.20.1). The depth and coverage on chromosomes were calculated with samtools^85^ (version 1.4) and bedtools^134^ (version 2.26.0). The methylation call for every cytosine was evaluated by Bismark and the methylation ratio was calculated as the number of reads supporting methylated Cs divided by the total unique reads covering the cytosine position (Supplementary Data 8).

### Detection of DMRs

The DMRs between different individuals and tissues were detected with metilene^135^ (version 0.2–7) for CG, CHG, and CHH nucleotide contexts. The mean methylation difference of each DMR had to be bigger than 0.1. Pathway enrichment analyses of DMR annotated genes were conducted with KOBAS^136^ (version 2.1.1).

### Combined analyses of the methylome and transcriptome

The coding genes located in DMRs in all three methylation contexts (CHH, CG, and CHG) and the differentially expressed genes (DEG) in RNA-seq data were identified. Pathway enrichment analysis was performed on genes that were both differentially expressed and had differential methylation between male basal meristems (MM) and young male leaves (MY) (Supplementary Fig. 20).

### Weighted gene co-expression network analyses

The DEGs were also analyzed using the edgeR R package (FDR < 0.05, logFC ≥1)^137^ between MM and MY. Then Gene Ontology (GO) enrichment analysis was performed. The expression levels of the genes involved GO result were used to construct the correlation network by using the WGCNA R package^138^.

### smRNA-seq and data analysis

Total RNA was extracted from *Welwitschia* using of TRIzol Reagent (Invitrogen, cat. NO 15596026) following the methods by Chomczynski et al.^139^. DNA digestion was carried out after RNA extraction by using DNase I. RNA quality was determined by examining A260/A280 using a NanodropTM OneC UV-Vis spectrophotometer (Thermo Fisher Scientific Inc). RNA integrity was confirmed by 1.5% (w/v) agarose gel electrophoresis. RNAs of suitable quality were identified using a Qubit3.0 with the QubitTM RNA Broad Range Assay kit (Life Technologies, Q10210).

A total of 1 μg RNA was used as input for microRNA (miRNA) library preparation using KC-DigitalTM smRNA Library Prep Kit for Illumina® (Catalog no. DR08602, Wuhan Seqhealth, China) following the manufacturer’s instructions. The kit is designed to eliminate duplication bias in PCR and sequencing steps by using unique molecular identifiers (UMI) of eight random bases to label the pre-amplified smRNA molecules. The eluted cDNA library was separated on a 6% w/v) PAGE gel. cDNA bands of ~160 bp were isolated, purified, and quantified by Qubit3.0, and finally sequenced on Hiseq X-10 sequencer (Illumina) with PE150 model.

Raw sequencing data were first filtered using the fastx_toolkit (version: 0.0.13.2) to discard low-quality reads and then adaptor sequences were trimmed using cutadapt^140^ (version: 1.15). Cleaned reads were further treated with in-house scripts to eliminate any remaining duplication bias introduced in library preparation and sequencing. Cleaned reads were clustered according to their unique molecular identifier (UMI) sequences, and reads with the same UMI sequence were grouped into the same cluster and then compared with each other by pairwise alignment. After all, sub-clusters were generated, multiple sequence alignment was performed to build consensus sequences for each sub-cluster. After these steps, errors and biases introduced by PCR amplification or sequencing were eliminated.

The consensus sequences from each sample were mapped to the reference genome of *Welwitschia*, using bowtie^141^ (version: 1.1.2) using default parameters. The package of mirdeep2^142^ (version:2.0.0.8) was used for mapping the reads to the known primary-miRNA in the miRBase^143^ database enabling predictions of novel miRNA. Any miRNAs that were differentially expressed between groups were identified using the edgeR package^137^ (version: 3.12.1). A cutoff with a *P* value <0.05 and | Log2Fold-change | > 1 was used to judge the statistical significance of miRNA expression differences. The target mRNAs of differentially expressed miRNAs were predicted using miRanda v3.3a.

To illustrate the functional differences between smRNAs, we compared the genome-wide distribution of smRNAs of individual lengths between 21 and 25 nt. The reads were re-aligned to the genome using bowtie and the distribution of reads on genomic regions was calculated using samtools.

### Measurement of phytohormones and determination of endogenous brassinosteroid level

Five biological replicates for each type of tissue (meristematic tissue, young section of leaf, and older section of leaf) were ground to a powder in liquid nitrogen. All the samples were obtained from five individuals grown in Wuhan Botanical Garden, CAS (all individuals were secondarily introduced from FLBG. The accession numbers of the five selected individuals were: SZBG 00052743, SZBG 00052744, SZBG 00052745, SZBG 00052746, SZBG 00052747).

Phytohormones were extracted from the powder at 4 °C for 12 h with 1 mL ethyl acetate. The supernatant was collected after centrifugation (14,000 × *g*, 20 min, 4 °C), after which the pellet was again extracted with 0.5 mL ethyl acetate at 4 °C for 1 hour. The supernatant from the second extraction was pooled with the first extraction. The supernatant was evaporated to dryness under N_2_ and the residue was resuspended in 0.1 mL of 50% acetonitrile (v/v). After being centrifuged (14,000 × *g*, 20 min, 4 °C), the supernatant was then analyzed by HPLC-ESI-MS/MS at Shanghai Applied Protein Technology company (Shanghai, China). The mobile phase consisted of a combination of solvent A (0.05% formic acid in water, v/v) and solvent B (0.05% formic acid in acetonitrile, v/v). The linear gradient was as follows: 2%-98% B (v/v) for 10 min, 2% B (v/v) for 10.1 min, and held at 2% B to 13 min. The mass spectrometer (Qtrap 5500 System, AB Sciex, Concord, Canada) equipped with an electrospray ionization (ESI) source was operated in positive/negative ionization and multiple reaction monitoring (MRM) modes. The MS parameters were set as follows: source temperature, 500 °C; ion source gas1 (GS1), 45 psi; ion source gas 2 (GS2), 45 psi; curtain gas, 30 psi; and ion spray voltage, 4500 V.

BRs are steroid hormones ubiquitously found in plants and are essential for normal plant growth^144^. Our comparative analyses of the transcriptome between basal meristematic tissue and young leaf material of *Welwitschia* indicated that the synthesis of BRs may be more active in the meristem. To further address this, the quantification of endogenous BRs was performed based on the method reported previously with some simplifications in sample pretreatment^145^. In brief, the harvested plant materials were first ground to a fine powder. Then 200 mg of the powder was extracted with 90% aqueous methanol (MeOH) in an ultrasonic bath for 1 hour.

D3-castasterone (D3-CS) was added to the extract as an internal standard for BR content measurement. After the mixed-mode cation exchange (MCX) solid-phase extraction cartridge was activated and equilibrated with MeOH, water, and 40% MeOH in sequence, and the crude extracts suspended in 40% MeOH were loaded onto the cartridge. The MCX cartridge was washed with 40% MeOH and then BRs were eluted with MeOH. After drying in a stream of N_2_, the eluent was redissolved with acetonitrile (ACN) to make a BR derivative using 2-methoxypyridine-5-boronic acid (MPyBA) prior to ultra-performance liquid chromatography-tandem mass spectrometry (UPLC-MS/MS) analysis. The analysis of BRs was performed on a quadrupole linear ion trap hybrid MS (QTRAP 5500, AB SCIEX) equipped with an EI source coupled with a UPLC (Waters). The UPLC inlet method, ESI source parameters, MRM transitions, and the related compound-dependent parameters were set as described previously^145^. In brief, 5 μL of each sample was injected onto a BEH C18 column (100 mm × 2.1 mm, 1.7 μm). The inlet method was set as follows: mobile phase A, 0.05% (v/v) acetic acid in water, and B, 0.05% (v/v) acetic acid in ACN. Gradient: 0 to 3 min, 65% B to 75% B; 3 to 11 min, 75% B to 95% B; 11 to 13 min, 95% B; 13 to 14.5 min, 95% B to 65% B; and 14.5 to 16 min, 65% B. CS and D_3_-CS was detected in positive MRM mode using the MRM transition 594.4 > 190.1 and 597.4 > 190.1, respectively. The ESI source parameters were set as ion spray voltage, 5000 V; desolvation temperature, 550 °C; nebulizing gas1, 45; desolvation gas 2, 45; and curtain gas, 30. As for CS and D3-CS, the MRM transition 582.4 > 178.1 and 585.4 > 178.1 was used for quantification. The results showed that the concentration of D3-CS was significantly higher in meristematic tissue (~3 pg/mg on average) than in the leaf (~2 pg/mg on average) (Supplementary Fig. 9).

### Estimation of the growth rate of leaves

A light scratch mark was made across a section of a particular leaf where it emerges from the basal meristem and at the next monthly visit, the distance of this mark from the basal meristem was measured (±0.1 mm) with callipers near the middle of the leaf width (Supplementary Fig. 22). The age of leaf sections estimated in this study was calculated from the total internal length between the young and older leaf section divided by the average growth rate.

### Characterization of specific expanded gene families

The OrthoMCL^146^ (version 2.0, https://orthomcl.org/orthomcl/) clustering method was used to classify the complete proteomes of 12 sequenced land plant genomes, including *Welwitschia* (Supplementary Table 2), into orthologous gene lineages (that is, orthogroups). In the first step, pairwise sequence similarities between all input protein sequences were calculated using BLASTP with an *e* value cutoff of 1e-05. Markov clustering of the resulting similarity matrix was used to define the ortholog cluster structure, using an inflation value (-I) of 1.5 (OrthoMCL default). We selected the following taxa to represent all major land plant and green algal lineages, including two core eudicots (*A. thaliana* and *S. lycopersicum*), two monocots (*O. sativa* and *Zea mays*), two early-diverging angiosperms (*Amborella* and *Liriodendron chinense*), three gymnosperms (*Gnetum*, *Ginkgo* and *Welwitschia*), two ferns (*A. filiculoides* and *S. cucullata*), one bryophyte (*P. patens*). In total, 55,913 orthogroups containing at least two genes were circumscribed of which 12,584 contained at least one gene from *Welwitschia* (Supplementary Fig. 10a).

All the protein-coding genes of *Welwitschia* and other representative seed plants were searched by PfamScan^147^ (version 1.6, http://pfam.xfam.org/, with default parameters) using Pfam database version 32.0. The number of pfam domains in each species was counted. Fisher’s exact test method was used to calculate a *P* value of each orthogroup or pfam domain to check whether the number for *Welwitschia* had expanded or contracted compared to other species. False discovery rate was used to get the adjusted *P* value (Supplementary Fig. 10b).

RNA-Seq reads from different tissues were mapped to the genome using TopHat^94^ (version 2.0.13, http://ccb.jhu.edu/software/tophat, with parameters–max-intron-length 500000 -m 2–library-type fr-unstranded). Htseq-count^148^ (version 0.11.2, https://htseq.readthedocs.io/en/master/count.html, with default parameters) to count the total number of aligned reads (read count). The total number of aligned reads (read counts) for each gene was normalized to the reads per kilobase exon model per million mapped reads^149^. Tandemly duplicated genes were searched for using MCScanX^150^ (version 1.0, http://chibba.pgml.uga.edu/mcscan2/, with default parameters).

### Reporting summary

Further information on research design is available in the Nature Research Reporting Summary linked to this article.

## Supplementary information

Supplementary Information

Review file

Supplementary Data

Description of Additional Supplementary Files

Reporting Summary

## Data Availability

Data supporting the findings of this work are available within the paper and its Supplementary Information files. A reporting summary for this article is available as a Supplementary Information file. The *Welwitschia* genome project data has been deposited at the NCBI under the BioProject number PRJNA680422. The whole-genome-sequencing data were deposited in the Sequence Read Archive database under the accession number SAMN16953877. The *Welwtschia* and *Gnetum* assemblies, gene sequences, and annotation data are also available at Dryad [10.5061/dryad.ht76hdrdr] or China National GeneBank DataBase [https://db.cngb.org/search/project/CNP0001943/]. Source data are provided with this paper.

## Source data

Source Data

## References

[1] Jürgens N, Oncken I, Oldeland J, Gunter F, Rudolph B (2021). Welwitschia: phylogeography of a living fossil, diversified within a desert refuge. Sci. Rep..

[2] Herre H (1961). The age of Welwitschia bainesii (Hook. f) Cearr.: C14 research. S. Afr. J. Bot..

[3] Bornman CH (1977). Welwitschia mirabilis: structural and functional anomalies. Madoqua.

[4] Talalaj, S., Talalaj, D. & Talalaj, J. The strangest plants in the world. (Hill of Content, 1991).

[5] Hooker JI (1862). On Welwitschia, a new genus of Gnetaceæ. Trans. Linn. Soc. Lond..

[6] Friedman WE (2015). Development and evolution of the female gametophyte and fertilization process in Welwitschia mirabilis (Welwitschiaceae). Am. J. Bot..

[7] Leebens-Mack JH (2019). One thousand plant transcriptomes and the phylogenomics of green plants. Nature.

[8] Dilcher DL, Bernardes-De-Oliveira ME, Pons D (2005). Welwitschiaceae from the lower Cretaceous of northeastern Brazil. Am. J. Bot..

[9] Wickett NJ (2014). Phylotranscriptomic analysis of the origin and early diversification of land plants. Proc. Natl Acad. Sci. USA.

[10] Li Z (2017). Single-copy genes as molecular markers for phylogenomic studies in seed plants. Genome Biol. Evol..

[11] Doyle JA (2012). Molecular and fossil evidence on the origin of angiosperms. Annu. Rev. Earth Planet. Sci..

[12] Bateman R (2019). Hunting the Snark: the flawed search for mythical Jurassic angiosperms. J. Exp. Bot..

[13] Wan T (2018). A genome for gnetophytes and early evolution of seed plants. Nat. Plants.

[14] Leitch IJ, Hanson L, Winfield M, Parker J, Bennett MD (2001). Nuclear DNA C-values complete familial representation in gymnosperms. Ann. Bot..

[15] Khoshoo TN, Ahuja MR (1963). The chromosomes and relationships of Welwitschia mirabilis. Chromosoma.

[16] Li Z (2015). Early genome duplications in conifers and other seed plants. Sci. Adv..

[17] Van de Peer Y (2004). Computational approaches to unveiling ancient genome duplications. Nat. Rev. Genet.

[18] Zhang Q-J (2020). The chromosome-level reference genome of tea tree unveils recent bursts of non-autonomous LTR retrotransposons to drive genome size evolution. Mol. Plant.

[19] Zhang QJ, Gao LZ (2017). Rapid and recent evolution of LTR retrotransposons drives rice genome evolution during the speciation of AA-genome Oryza species. G3 (Bethesda, Md.).

[20] Cossu RM (2017). LTR retrotransposons show low levels of unequal recombination and high rates of intraelement gene conversion in large plant genomes. Genome Biol. Evol..

[21] Roddy, A. et al. The scaling of genome size and cell size limits maximum rates of photosynthesis with implications for ecological strategies. *Int. J. Plant. Sci.*10.1101/619585 (2019).

[22] Ausin I (2016). DNA methylome of the 20-gigabase Norway spruce genome. Proc. Natl Acad. Sci. USA.

[23] Takuno S, Ran J-H, Gaut BS (2016). Evolutionary patterns of genic DNA methylation vary across land plants. Nat. Plants.

[24] Niederhuth CE (2016). Widespread natural variation of DNA methylation within angiosperms. Genome Biol..

[25] Zhang X (2006). Genome-wide high-resolution mapping and functional analysis of DNA methylation in Arabidopsis. Cell.

[26] Matzke MA, Kanno T, Matzke AJM (2015). RNA-Directed DNA methylation: the evolution of a complex epigenetic pathway in flowering plants. Annu. Rev. Plant Biol..

[27] Johnsen Ø (2005). Climatic adaptation in Picea abies progenies is affected by the temperature during zygotic embryogenesis and seed maturation. Plant Cell Environ..

[28] Yakovlev IA, Carneros E, Lee Y, Olsen JE, Fossdal CG (2016). Transcriptional profiling of epigenetic regulators in somatic embryos during temperature induced formation of an epigenetic memory in Norway spruce. Planta.

[29] Trávníček P (2019). Diversity in genome size and GC content shows adaptive potential in orchids and is closely linked to partial endoreplication, plant life-history traits and climatic conditions. N. Phytol..

[30] Cacciò S (1997). Methylation patterns in the isochores of vertebrate genomes. Gene.

[31] Serres-Giardi L, Belkhir K, David J, Glémin S (2012). Patterns and evolution of nucleotide landscapes in seed plants. Plant Cell.

[32] Ossowski S (2010). The rate and molecular spectrum of spontaneous mutations in Arabidopsis thaliana. Science.

[33] Glémin S (2010). Surprising fitness consequences of GC-biased gene conversion: I. Mutation load and inbreeding depression. Genetics.

[34] Vinogradov AE (2003). DNA helix: the importance of being GC-rich. Nucleic Acids Res..

[35] Rocha EP, Danchin A (2002). Base composition bias might result from competition for metabolic resources. Trends Genet..

[36] Shenhav L, Zeevi D (2020). Resource conservation manifests in the genetic code. Science.

[37] Kelly S (2018). The amount of nitrogen used for photosynthesis modulates molecular evolution in plants. Mol. Biol. Evol..

[38] Martens P (1977). Welwitschia mirabilis and neoteny. Am. J. Bot..

[39] Robert JR (1958). Leaf anatomy of Welwitschia. i. Early development of the leaf. Am. J. Bot..

[40] Bornman CH (1972). Welwitschia mirabilis: paradox of the Namib Desert. Endeavour.

[41] Pham T, Sinha N (2003). Role of KNOX genes in shoot development of Welwitschia mirabilis. Int. J. Plant Sci..

[42] Nishii K (2010). A complex case of simple leaves: indeterminate leaves co-express ARP and KNOX1 genes. Dev. Genes Evol..

[43] Hacham Y (2011). Brassinosteroid perception in the epidermis controls root meristem size. Dev. (Camb., Engl.).

[44] Sun S (2015). Brassinosteroid signalling regulates leaf erectness in Oryza sativa via the control of a specific U-type cyclin and cell proliferation. Dev. Cell.

[45] Wei Z, Li J (2016). Brassinosteroids regulate root growth, development, and symbiosis. Mol. Plant.

[46] Jiang CK, Rao GY (2020). Insights into the diversification and evolution of R2R3-MYB transcription factors in plants. Plant Physiol..

[47] Dubos C (2010). MYB transcription factors in Arabidopsis. Trends Plant Sci..

[48] Pandey A, Misra P, Trivedi PK (2015). Constitutive expression of Arabidopsis MYB transcription factor, AtMYB11, in tobacco modulates flavonoid biosynthesis in favor of flavonol accumulation. Plant Cell Rep..

[49] Petroni K (2008). The AtMYB11 gene from Arabidopsis is expressed in meristematic cells and modulates growth in planta and organogenesis in vitro. J. Exp. Bot..

[50] Gugger PF, Peñaloza-Ramírez JM, Wright JW, Sork VL (2017). Whole-transcriptome response to water stress in a California endemic oak, Quercus lobata. Tree Physiol..

[51] Plomion C (2018). Oak genome reveals facets of long lifespan. Nat. Plants.

[52] Jaiwal SKCA, Mahajan S, Kumar S, Sharma VK (2021). The genome sequence of Aloe vera reveals adaptive evolution of drought tolerance mechanisms. iScience.

[53] Henschel JR, Seely MK (2000). Long-term growth patterns of Welwitschia mirabilis, a long-lived plant of the Namib desert (including a bibliography). Plant Ecol..

[54] Stortenbeker N, Bemer M (2019). The SAUR gene family: the plant’s toolbox for adaptation of growth and development. J. Exp. Bot..

[55] Wei J (2015). The E3 ligase AtCHIP positively regulates Clp proteolytic subunit homeostasis. J. Exp. Bot..

[56] Olinares PD, Kim J, Davis JI, van Wijk KJ (2011). Subunit stoichiometry, evolution, and functional implications of an asymmetric plant plastid ClpP/R protease complex in Arabidopsis. Plant Cell.

[57] Sjögren LL, Stanne TM, Zheng B, Sutinen S, Clarke AK (2006). Structural and functional insights into the chloroplast ATP-dependent Clp protease in Arabidopsis. Plant Cell.

[58] Dong H (2013). A rice virescent-yellow leaf mutant reveals new insights into the role and assembly of plastid caseinolytic protease in higher plants. Plant Physiol..

[59] Nakabayashi K, Ito M, Kiyosue T, Shinozaki K, Watanabe A (1999). Identification of clp genes expressed in senescing Arabidopsis leaves. Plant cell Physiol..

[60] Koussevitzky S (2007). An Arabidopsis thaliana virescent mutant reveals a role for ClpR1 in plastid development. Plant Mol. Biol..

[61] Vierling E (1991). The roles of heat shock proteins in plants. Annu. Rev. Plant Physiol. Plant Mol. Biol..

[62] Guo LM, Li J, He J, Liu H, Zhang HM (2020). A class I cytosolic HSP20 of rice enhances heat and salt tolerance in different organisms. Sci. Rep..

[63] Waseem M, Rong X, Li Z (2019). Dissecting the role of a basic helix-loop-helix transcription factor, SlbHLH22, under salt and drought stresses in transgenic Solanum lycopersicum L. Front. Plant Sci..

[64] De La Torre AR, Lin YC, Van de Peer Y, Ingvarsson PK (2015). Genome-wide analysis reveals diverged patterns of codon bias, gene expression, and rates of sequence evolution in Picea gene families. Genome Biol. Evol..

[65] Neale DB, Martínez-García PJ, De La Torre AR, Montanari S, Wei XX (2017). Novel insights into tree biology and genome evolution as revealed through genomics. Annu. Rev. Plant Biol..

[66] Nakashima K, Yamaguchi-Shinozaki K, Shinozaki K (2014). The transcriptional regulatory network in the drought response and its crosstalk in abiotic stress responses including drought, cold, and heat. Front. Plant Sci..

[67] Jiang F (2019). The apricot (Prunus armeniaca L.) genome elucidates Rosaceae evolution and beta-carotenoid synthesis. Hortic. Res..

[68] Huo H, Dahal P, Kunusoth K, McCallum CM, Bradford KJ (2013). Expression of 9-cis-EPOXYCAROTENOID DIOXYGENASE4 is essential for thermoinhibition of lettuce seed germination but not for seed development or stress tolerance. Plant Cell.

[69] Wang H (2015). CG gene body DNA methylation changes and evolution of duplicated genes in cassava. Proc. Natl Acad. Sci. USA.

[70] Xu J (2018). Single-base methylome analysis reveals dynamic epigenomic differences associated with water deficit in apple. Plant Biotechnol. J..

[71] Friis EM, Pedersen KR, Crane PR (2014). Welwitschioid diversity in the early Cretaceous: evidence from fossil seeds with pollen from Portugal and eastern North America. Grana.

[72] Damme PV, Vernemmen P (1992). The natural environment of the Namib Desert. Afr. Focus.

[73] Siesser WG (1980). Late Miocene origin of the Benguela upswelling system off northern Namibia. Science.

[74] Meyers, P. A., Brassell, S. C., Huc, A. Y., Barron, E. J. & Stradner, H. Organic geochemistry of sediments recovered by DSDP/IPOD Leg 75 from under the Benguela current. Volume 10, pp.14. (Plenum Press, 1983).

[75] Alzohairy AM, Yousef MA, Edris S, Kerti B, Alzohairy M (2012). Detection of LTR retrotransposons reactivation induced by in vitro environmental stresses in barley (Hordeum vulgare) via RT-qPCR. Life Sci. J..

[76] Morano A (2014). Targeted DNA methylation by homology-directed repair in mammalian cells. Transcription reshapes methylation on the repaired gene. Nucleic Acids Res..

[77] Russo G (2016). DNA damage and repair modify DNA methylation and chromatin domain of the targeted locus: mechanism of allele methylation polymorphism. Sci. Rep..

[78] Doerfler W (2006). The almost-forgotten fifth nucleotide in DNA: an introduction. Curr. Top. Microbiol. Immunol..

[79] Nystedt B (2013). The Norway spruce genome sequence and conifer genome evolution. Nature.

[80] Guignard M (2017). Impacts of nitrogen and phosphorus: from genomes to natural ecosystems and agriculture. Front. Ecol. Evol..

[81] Drake PL, Froend RH, Franks PJ (2013). Smaller, faster stomata: scaling of stomatal size, rate of response, and stomatal conductance. J. Exp. Bot..

[82] Massmann U (1976). Welwitschia: nach 90 jahren. Namib. und Meer.

[83] Ruan J, Li H (2020). Fast and accurate long-read assembly with wtdbg2. Nat. Methods.

[84] Hu J, Fan J, Sun Z, Liu S (2020). NextPolish: a fast and efficient genome polishing tool for long-read assembly. Bioinformatics.

[85] Li H (2009). The sequence alignment/map format and SAMtools. Bioinformatics.

[86] Xu Z, Wang H (2007). LTR_FINDER: an efficient tool for the prediction of full-length LTR retrotransposons. Nucleic Acids Res..

[87] Edgar RC, Myers EW (2005). PILER: identification and classification of genomic repeats. Bioinformatics.

[88] Price AL, Jones NC, Pevzner PA (2005). De novo identification of repeat families in large genomes. Bioinformatics.

[89] Benson G (1999). Tandem repeats finder: a program to analyze DNA sequences. Nucleic Acids Res..

[90] Stanke M, Diekhans M, Baertsch R, Haussler D (2008). Using native and syntenically mapped cDNA alignments to improve de novo gene finding. Bioinformatics.

[91] Korf I (2004). Gene finding in novel genomes. BMC Bioinformatics.

[92] Burge C, Karlin S (1997). Prediction of complete gene structures in human genomic DNA. J. Mol. Biol..

[93] Keilwagen J (2016). Using intron position conservation for homology-based gene prediction. Nucleic Acids Res..

[94] Trapnell C, Pachter L, Salzberg SL (2009). TopHat: discovering splice junctions with RNA-Seq. Bioinformatics.

[95] Haas BJ (2003). Improving the Arabidopsis genome annotation using maximal transcript alignment assemblies. Nucleic Acids Res..

[96] Haas BJ (2008). Automated eukaryotic gene structure annotation using EVidenceModeler and the program to assemble spliced alignments. Genome Biol..

[97] Mitchell AL (2019). InterPro in 2019: improving coverage, classification and access to protein sequence annotations. Nucleic Acids Res..

[98] Vanneste K, Van de Peer Y, Maere S (2013). Inference of genome duplications from age distributions revisited. Mol. Biol. Evol..

[99] Enright AJ, Van Dongen S, Ouzounis CA (2002). An efficient algorithm for large-scale detection of protein families. Nucleic Acids Res..

[100] Edgar RC (2004). MUSCLE: multiple sequence alignment with high accuracy and high throughput. Nucleic Acids Res..

[101] Goldman N, Yang Z (1994). A codon-based model of nucleotide substitution for protein-coding DNA sequences. Mol. Biol. Evol..

[102] Yang Z (2007). PAML 4: phylogenetic analysis by maximum likelihood. Mol. Biol. Evolution..

[103] Guindon S (2010). New algorithms and methods to estimate maximum-likelihood phylogenies: assessing the performance of PhyML 3.0. Syst. Biol..

[104] Proost S (2012). i-ADHoRe 3.0–fast and sensitive detection of genomic homology in extremely large data sets. Nucleic Acids Res..

[105] Fostier J (2011). A greedy, graph-based algorithm for the alignment of multiple homologous gene lists. Bioinformatics.

[106] Putnam NH (2007). Sea anemone genome reveals ancestral eumetazoan gene repertoire and genomic organization. Science.

[107] Gu Z, Gu L, Eils R, Schlesner M, Brors B (2014). Circlize Implements and enhances circular visualization in R. Bioinformatics.

[108] Guy L, Kultima JR, Andersson S (2010). G. genoPlotR: comparative gene and genome visualization in R. Bioinformatics.

[109] Moreno-Hagelsieb G, Latimer K (2008). Choosing BLAST options for better detection of orthologs as reciprocal best hits. Bioinformatics.

[110] Vanneste K, Baele G, Maere S, Van de Peer Y (2014). Analysis of 41 plant genomes supports a wave of successful genome duplications in association with the Cretaceous-Paleogene boundary. Genome Res..

[111] Ostlund G (2010). InParanoid 7: new algorithms and tools for eukaryotic orthology analysis. Nucleic Acids Res..

[112] D’Hont A (2012). The banana (Musa acuminata) genome and the evolution of monocotyledonous plants. Nature.

[113] Drummond AJ, Suchard MA, Xie D, Rambaut A (2012). Bayesian phylogenetics with BEAUti and the BEAST 1.7. Mol. Biol. Evol..

[114] Group AP (2016). An update of the angiosperm phylogeny group classification for the orders and families of flowering plants: APG IV. Bot. J. Linn. Soc..

[115] Gandolfo M, Nixon K, Crepet W (1998). A new fossil flower from the Turonian of New Jersey: Dressiantha bicarpellata gen. et sp. nov. (Ceapparales). Am. J. Bot..

[116] Beilstein MA, Nagalingum NS, Clements MD, Manchester SR, Mathews S (2010). Dated molecular phylogenies indicate a Miocene origin for Arabidopsis thaliana. Proc. Natl Acad. Sci. USA.

[117] Crepet W, Nixon K (1998). Fossil Clusiaceae from the late Cretaceous (Turonian) of new Jersey and implications regarding the history of bee pollination. Am. J. Bot..

[118] Xi Z (2012). Phylogenomics and a posteriori data partitioning resolve the Cretaceous angiosperm radiation Malpighiales. Proc. Natl Acad. Sci. USA.

[119] Friis EM (1988). Spirematospermum chandlerae sp. nov., an extinct species of Zingiberaceae from the North American Cretaceous. Tert. Res..

[120] Janssen T, Bremer K (2004). The age of major monocot groups inferred from 800+rbcL sequences. Bot. J. Linn. Soc..

[121] Doyle JA (2005). Early evolution of angiosperm pollen as inferred from molecular and morphological phylogenetic analyses. Grana.

[122] Rydin C, Pedersen KR, Friis EM (2004). On the evolutionary history of Ephedra: cretaceous fossils and extant molecules. Proc. Natl Acad. Sci. USA.

[123] Magallón S (2010). Using fossils to break long branches in molecular dating: a comparison of relaxed clocks applied to the origin of angiosperms. Syst. Biol..

[124] Clarke JT, Warnock RC, Donoghue PC (2011). Establishing a time-scale for plant evolution. N. phytologist.

[125] Heled J, Drummond AJ (2012). Calibrated tree priors for relaxed phylogenetics and divergence time estimation. Syst. Biol..

[126] Llorens C (2011). The Gypsy Database (GyDB) of mobile genetic elements: release 2.0. Nucleic Acids Res..

[127] Altschul SF, Gish W, Miller W, Myers EW, Lipman DJ (1990). Basic local alignment search tool. J. Mol. Biol..

[128] Yu XJ, Zheng HK, Wang J, Wang W, Su B (2006). Detecting lineage-specific adaptive evolution of brain-expressed genes in human using rhesus macaque as outgroup. Genomics.

[129] Birney E, Clamp M, Durbin R (2004). GeneWise and genomewise. Genome Res..

[130] Vilella AJ (2009). EnsemblCompara geneTrees: complete, duplication-aware phylogenetic trees in vertebrates. Genome Res..

[131] Seberg O, Petersen G (2009). A unified classification system for eukaryotic transposable elements should reflect their phylogeny. Nat. Rev. Genet..

[132] Chen Y (2018). SOAPnuke: a MapReduce acceleration-supported software for integrated quality control and preprocessing of high-throughput sequencing data. Gigascience.

[133] Krueger F, Andrews SR (2011). Bismark: a flexible aligner and methylation caller for Bisulfite-Seq applications. Bioinformatics.

[134] Quinlan AR, Hall IM (2010). BEDTools: a flexible suite of utilities for comparing genomic features. Bioinformatics.

[135] Jühling F (2016). Metilene: fast and sensitive calling of differentially methylated regions from bisulfite sequencing data. Genome Res..

[136] Xie C (2011). KOBAS 2.0: a web server for annotation and identification of enriched pathways and diseases. Nucleic Acids Res..

[137] Robinson MD, McCarthy DJ, Smyth GK (2010). edgeR: a Bioconductor package for differential expression analysis of digital gene expression data. Bioinformatics.

[138] Langfelder P, Horvath S (2008). WGCNA: an R package for weighted correlation network analysis. BMC Bioinformatics.

[139] Chomczynski P, Sacchi N (1987). Single-step method of RNA isolation by acid guanidinium thiocyanate-phenol-chloroform extraction. Anal. Biochem..

[140] Kechin A, Boyarskikh U, Kel A, Filipenko M (2017). CutPrimers: a new tool for accurate cutting of primers from reads of targeted next generation sequencing. J. Comput. Biol..

[141] Langmead B, Trapnell C, Pop M, Salzberg SL (2009). Ultrafast and memory-efficient alignment of short DNA sequences to the human genome. Genome Biol..

[142] Friedländer MR, Mackowiak SD, Li N, Chen W, Rajewsky N (2012). miRDeep2 accurately identifies known and hundreds of novel microRNA genes in seven animal clades. Nucleic Acids Res..

[143] Kozomara A, Birgaoanu M, Griffiths-Jones S (2019). miRBase: from microRNA sequences to function. Nucleic Acids Res..

[144] Li Z, He Y (2020). Roles of brassinosteroids in plant reproduction. Int. J. Mol. Sci..

[145] Xin P, Yan J, Fan J, Chu J, Yan C (2013). An improved simplified high-sensitivity quantification method for determining brassinosteroids in different tissues of rice and Arabidopsis. Plant Physiol..

[146] Li L, Stoeckert CJ, Roos DS (2003). OrthoMCL: identification of ortholog groups for eukaryotic genomes. Genome Res..

[147] Finn RD (2014). Pfam: the protein families database. Nucleic Acids Res..

[148] Anders S, Pyl PT, Huber W (2015). HTSeq–a Python framework to work with high-throughput sequencing data. Bioinformatics.

[149] Mortazavi A, Williams BA, McCue K, Schaeffer L, Wold B (2008). Mapping and quantifying mammalian transcriptomes by RNA-Seq. Nat. Methods.

[150] Wang Y (2012). MCScanX: a toolkit for detection and evolutionary analysis of gene synteny and collinearity. Nucleic Acids Res..

