# Supplementary material for: The Welwitschia genome reveals a unique biology underpinning extreme longevity in deserts
Source: Nat Commun. 2021 Jul 12;12:4247. doi: 10.1038/s41467-021-24528-4 (PMC8275611; doi:10.1038/s41467-021-24528-4)
Supplement: Supplementary file 1 — Supplementary Information [file 41467_2021_24528_MOESM1_ESM.pdf]

The *Welwitschia* genome reveals unique biology underpinning extreme longevity in deserts

Wan *et al.*

Supplementary Note 1. An introduction to and structural anomalies of *Welwitschia.*

'It is out of the question the most wonderful plant ever brought to this country, and one of the ugliest.' This was the response of Joseph Hooker, the Regius Keeper of the Royal Botanic Gardens, Kew, in 1863 when presented with a plant of *Welwitschia mirabilis*, hereafter, *Welwitschia*. *Welwitschia* is unusual for its large, strap like leaves that grow continuously along the ground. Throughout its entire life, each plant produces only two leaves, which often split into many segments as a result of the leaves being whipped by the wind. Carbon-14 dating of the largest plants have shown that some individuals are over 1,500 years old (Supplementary Fig. 15).

Welwitschia belongs to the family Welwitschiaceae, which is monogeneric and monospecific. Together with two other monogeneric families Gnetaceae (*Gnetum*) and Ephedraceae (*Ephedra*) they comprise the Gnetales, a distinctive lineage within the gymnosperms. The phylogenetic position of *Welwitschia* as well as Gnetales in general has triggered much debate. The 'anthophyte hypothesis' whereby Gnetales are considered to be the sister group to angiosperms[1,2] was proposed based on shared morphological similarities with angiosperms (e.g. 'double fertilization'[3] and xylem vessel structure[4]). However, this hypothesis has only equivocal support from molecular sequence data[5,6]. Phylogenetic reconstructions using data from a variety of genic regions have given rise to the most widespread opinion that Gnetales are most closely related to the conifers, either as sister to Pinaceae ('Gnepine'), sister to cupressophytes ('Gnecup'), or sister to all conifers ('Gnetifer'), depending on the sequence(s) and analytical methods deployed[6,7].

Welwitschia is often considered to be a desert plant as its distribution is restricted to the arid to hyperarid western parts of Namibia and Angola, north of 23.5° S latitude. However, natural populations do not only occur in the central and northern Namib Desert, but are also abundant in adjacent, more mesic parts of the Arid Savanna (Fig. 1). *Welwitschia* has a short, unbranched stem and a massive woody concave crown bearing two huge strap-shaped leaves. The leaves function as permanent photosynthetic organs and continue to grow from a basal meristem throughout the life of the plant. Unlike other plants, the shoot apical meristem (SAM) of *Welwitschia* dies shortly after germination and the meristematic activity moves to the periphery of the crown. Borman et. al. (1972)[8] presented a longitudinal section through a young individual showing the insertion of leaves and the strobilus into the terminal groove. Here we used Nuclear Magnetic Resonance Imaging (MRI) to scan a young plant of *Welwitschia* (*ex-situ* specimen preserved in the Fairy Lake Botanical Garden for 5 years) to produce comparative images to further clarify the unusual morphological structures (Supplementary Fig. 16). Images of the reproductive organs taken from living specimens growing in the wild are also shown.

Supplementary Note 2. The DNA methylation landscape of *Welwitschia.*

For wild individuals of *Welwitschia*, we bisulfite-sequenced genomic DNA from two tissue types with three replicates for each type (meristematic tissue of male individuals (MM1, MM2, MM3), meristematic tissue of female individual (FM1, FM2, FM3), young section of leaf of male individual (MY1, MY2, MY3) and young section of leaf of female individual (FY1, FY2, FY3). The six individuals (MM1-MY1, MM2-MY2, MM3-MY3, FM1-FY1, FM2-FY2, FM3-FY3) were of different ages (years different), as estimated from the lengths of leaves and assumed slow growth rate[9,10], For a single greenhouse individual, we analysed three types of tissue. This comprised the central part of the meristematic tissue (CM), the peripheral part of the meristematic tissue (PM) and the mature leaf (L). In general, all samples showed that the genome of *Welwitschia* was highly methylated in CG, CHG context and little variation was observed between male and female individuals or between tissue types. In contrast, CHH patterns differed between tissue types (Supplementary Fig. 21).

Supplementary Note 3. Detection of differentially methylated regions.

Differentially methylated regions (DMRs) at CHH trinucleotides represent the majority of DMRs, and most of them were shown to be distributed in the intergenic regions (Supplementary Table 11). Furthermore, we found DMRs in CHH were most abundantly located in TE regions (see Fig. 4c, Supplementary Table 13).

Supplementary Note 4. Long-term monitoring program in Gobabeb station, Namibia and estimation of growth rate of *Welwitschia's* leaves.

Since 1980s, a long-term ecological research program has been conducted in Gobabeb (the training and research center which is located in the heart of the hyperarid Namib Desert and was established early in 1963). With over 14 years' monitoring, Henschel JR. and Seely MK[12] in 2000 presented a comprehensive analysis of the growth pattern of *Welwitschia*, including the growth rate of leaves, population structure and sex ratios. The general low rate of leaf growth rate was estimated to be 0.37 mm day^{-1} with fluctuating rates caused by episodic rainfall events. We conducted further measurements during 2014-2019 field trips, following the protocol adopted previously.

Supplementary Note 5. Expansion of *R2R3-MYB* genes.

R2R3-MYB proteins belong to the MYB transcription factors (TFs), one of the largest classes of TFs in plants comprising MYB-related, *R2R3-MYB*, *R1R2R3-MYB*, and *4R-MYB* proteins, with the size of the group mainly attributed to the rapid expansion of *R2R3-MYB*[13]. Previous studies have indicated that *R2R3-MYB* TFs are extensively involved in plant development, secondary metabolism, cell proliferation and stress response[13-15]. Due to lineage-specific gene loss or expansion, there is little consensus on the classification schemes of *R2R3-MYB*, including the number of subgroups.

A recent study suggested that at least ten subfamilies of *R2R3-MYB* might exist in ancestral

land plants and that more than half of the *R2R3-MYB* genes within land plant species belonged to subfamily VIII[16]. The number of repeats of the MYB DNA-binding (Pfam ID: PF00249) domain has been used to classify MYB proteins into different subfamilies with *R2R3-MYB* containing two repeats[17].

PfamScan[18] (version 1.6, http://pfam.xfam.org/, with default parameters) was used to search for MYB DNA-binding proteins in *Welwitschia* as well as in other land plants (Supplementary Table 2) and those proteins containing two MYB DNA-binding domains were extracted. Multiple sequence alignments were generated with the software MUSCLE[19] (version 3.8.31, http://www.drive5.com/muscle, with default parameters) and maximum-likelihood (ML) phylogenetic trees were constructed using FastTree (version 2.1.11, http://www.microbesonline.org/fasttree/treecmp.html, with default parameters). In our study, the scheme of clades was mainly based on the orthogroups described above, and was partly informed by the topology suggested in Jiang and Rao's investigation[16] . We found that gene members belonging to a subgroup comprising of *AtMYB11* and its paralogs *AtMYB12*, *AtMYB111* were expanded in *Welwitschia* (11 copies). In contrast, the other plant species studied had no more than five copies (Supplementary Fig. 11). *AtMYB11* and its paralogs have been widely investigated in *A. thaliana* where it has been proposed that they are essential to control cell proliferation[20]. Their expansion in the *Welwitschia* genome may have contributed to the slow growth rate of this species.

Supplementary Note 6. Expansion of small auxin up-regulated RNA genes.

Small auxin up-regulated RNA (*SAUR*) genes comprise a family of auxin-responsive genes with ~60-140 members in most higher plant species[21]. With recent advances in the characterization of the SAUR genes in *Arabidopsis* and other plant species, the major role of SAUR genes is now considered to be the regulation of cell elongation[22]. The auxin-inducible (Pfam ID:PF02519) domain was used to search for these proteins in *Welwitschia* and other land plants (see Note 5) using PfamScan[18] (version 1.6, http://pfam.xfam.org/, with default parameters). Proteins of each species which contained the auxin-inducible domain were extracted. Multiple sequence alignments were generated with the software MUSCLE[19] (version 3.8.31, http://www.drive5.com/muscle, with default parameters) and maximum-likelihood (ML) phylogenetic trees were constructed using FastTree (version 2.1.11, http://www.microbesonline.org/fasttree/treecmp.html, with default parameters). Subgroups were classified using *Arabidopsis* proteins[23]. A total of 78 *SAUR* genes were identified in *Welwitschia*, including a considerable expansion of genes in the subclade containing *AtSAUR58*, *AtSAUR43* and in the subclade of *AtSAUR17* (Supplementary Fig. 12).

Supplementary Note 7. Expansion of small Heat shock proteins.

The Small Heat Shock Proteins (sHSPs) comprise a major family of HSPs induced by heat stress in plants[24]. Among the sHSPs, the most abundant and complex members in higher plants belong to the HSP20 family[25,26]. We compared the number of members in *Welwitschia*

as well as in other land plants mentioned above. Proteins belonging to the *HSP20* family all contain a specific pfam domain (Pfam ID:PF00011[27]). This was used to search for HSP20 proteins in *Welwitschia* and other representative seed plants using PfamScan[18] (version 1.6, http://pfam.xfam.org/, with default parameters). Those proteins which contained pfam domain PF00011[27] were extracted and the multiple sequence alignments were generated using MUSCLE[19] (version 3.8.31, http://www.drive5.com/muscle, with default parameters). Finally, maximum-likelihood (ML) phylogenetic trees were constructed using FastTree (version 2.1.11, http://www.microbesonline.org/fasttree/treecmp.html, with default parameters). The HSP proteins in *Oryza saiva* were used to further classify HSP20 members into 12 subgroups[27] (C I, C II, C III, C IV, C V, C VI, C VII, P, Po, M I, M II, ER) (Supplementary Fig. 13).

Supplementary Note 8. ABA synthesis in *Welwitschia* and its preferential upregulation in the meristem and young leaf sections in response to drought stress.

Abscisic acid **(**ABA) has been implicated as a key component in response to drought and other environmental stress[28]. In combined analyses of the methylome and transcriptome, we observed that genes in the carotenoid biosynthesis pathway were differentially expressed between the basal meristem and leaf, whilst displayed differential methylation (Supplementary Fig. 14). We therefore retrieved the genes involved in the carotenoid biosynthesis and found that the *NCED4* (9-cis-epoxycarotenoid dioxygenase 4) gene, which is a member of the *NCED* family, was specifically expanded in *Welwitschia* giving rise to16 copies. In contrast, only one or two copies have been observed in other seed plants that have been analysed to date (Supplementary Fig. 14). Moreover, a cluster of copies, assumed to have arisen via tandem duplication, showed significantly increased expression in the basal meristem compared with other tissues examined, suggestive of an important function in this tissue.

It is noticeable that we identified one *NCED4* gene, *W. mirabilis02116* whose promoter was hypomethylated at CHH sites in young leaf tissue, and which also showed elevated expression compared to other tissues. The complete information on the methylation status of *W. mirabilis02116* was retrieved and aligned to the reference genome. Fig. 4f shows the dynamic changes in CHH methylation including genome coordinate and gene structure information.

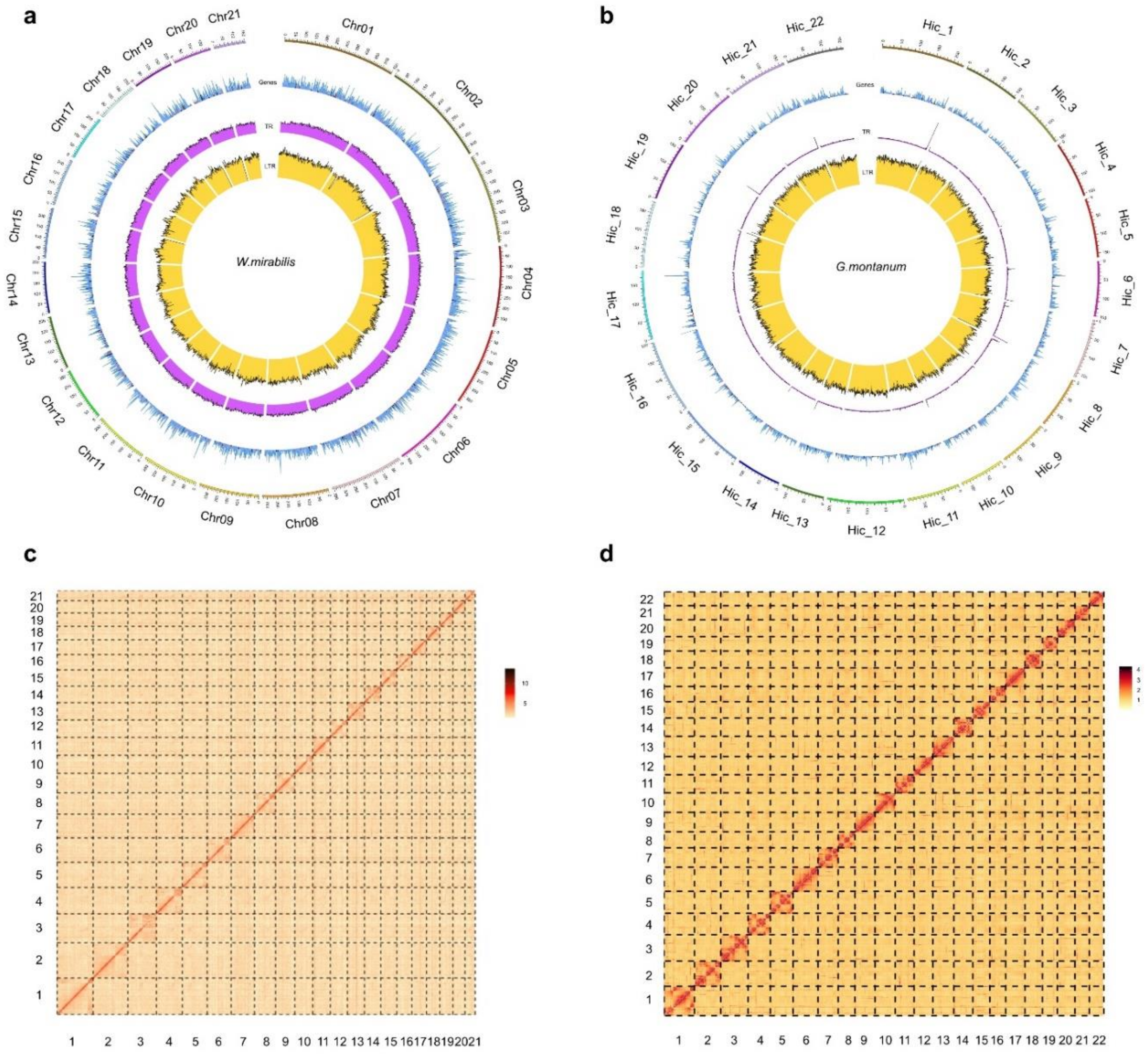

Supplementary Figure 1. **Chromosome level assembly maps of two gnetophytes. a** Assembly of 21 chromosomes of *Welwitschia* (optical and HiC), and; **b** of 22 chromosomes of *Gnetum* (HiC). The light blue circles represent gene densities, purple circles represent the distribution of tandem repetitive sequences (TR) along the chromosomes, yellow circles represent the distribution of Long Terminal Repeat retrotransposons (LTRs) along the chromosomes. **c, d** Contact maps for chromosomes of *Welwitschia* and *Gnetum* respectively.

a

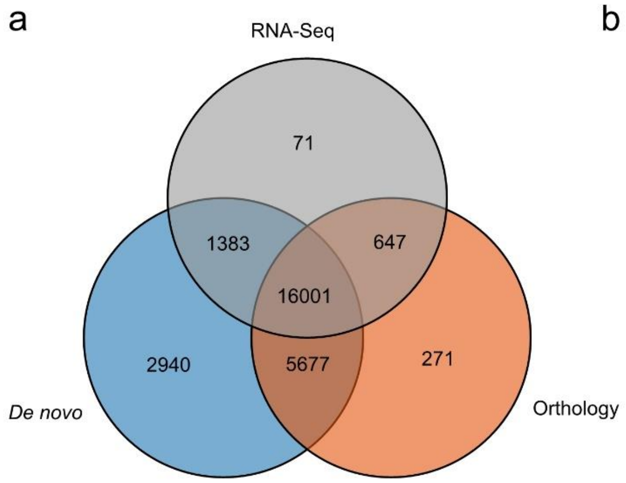

b

Type		number
RNA-seq	single sample	14
	mutiple samples	57
Orthology	single species	17
	mutiple species	254
De novo	single model	352
	mutiple models	2,588
Orthology+RNA-seq		647
De novo+RNA-seq		1,383
De novo+Orthology		5,677
De novo+Orthology+RNA-seq		16,001
Total		26,990

c

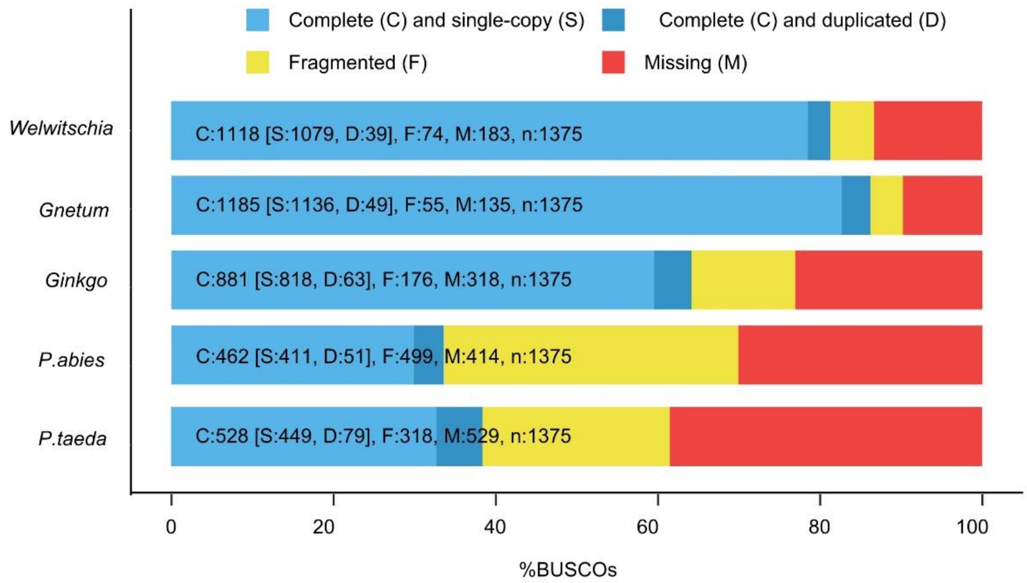

Supplementary Figure 2. **Annotation and assembly quality assessment. a** Venn diagram and **b** detailed breakdown showing number of genes identified by *de novo* assembly, RNA-seq, and by orthology with existing protein sequences. A total of 24,050（89.11%）of all 26,990 genes identified were supported by evidence from orthology or RNA-seq. **c** BUSCO (Benchmarking Universal Single-Copy Orthologs) analysis of gnetophytes and the available representative gymnosperm genomes.

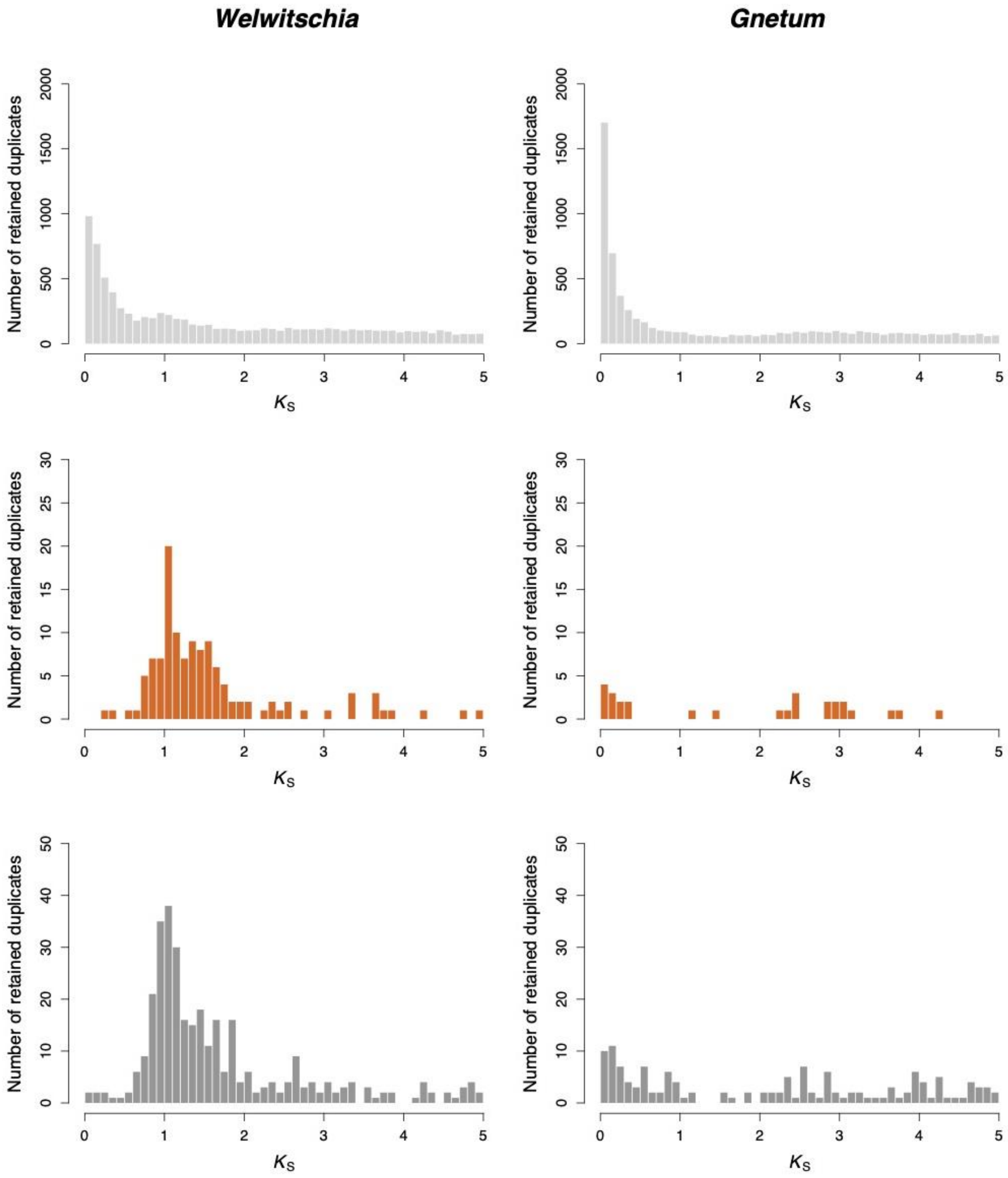

Supplementary Figure 3. Distributions of synonymous substitutions per synonymous site (K_S) in *Welwitschia* and *Gnetum* for the whole paranome (top row), anchor pairs in collinear regions (middle row), and anchor pairs in syntenic regions (bottom row). Please note that the *Gnetum* genome has few collinear and syntenic regions.

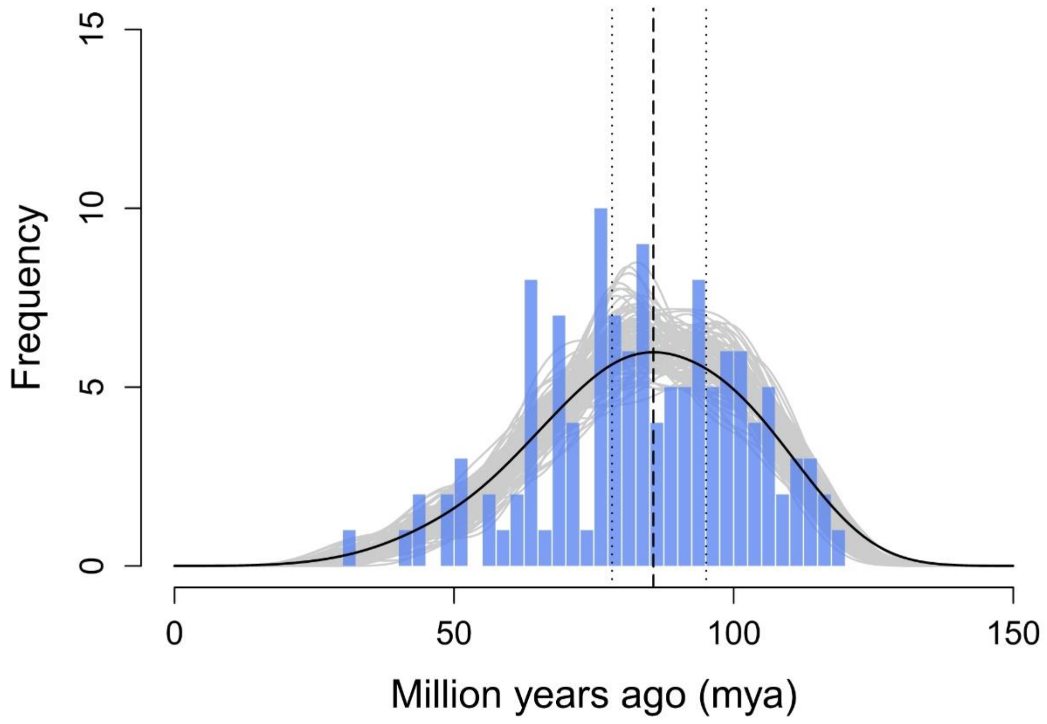

Supplementary Figure 4. Absolute age of the *Welwitschia* WGD event. Absolute age distribution obtained by phylogenomic dating of *Welwitschia* paralogs. The solid black line represents the kernel density estimates (KDE) of the dated paralogs, and the vertical dashed black lines represents its peak (mode) at 86 mya, which was used as the consensus WGD age estimate. The grey lines represent density estimates from 2,500 bootstrap replicates and the vertical black dotted lines represent the 90% confidence intervals for the WGD age estimate, giving a range of 78 – 96 mya. The histogram shows the raw distribution of dated paralogs.

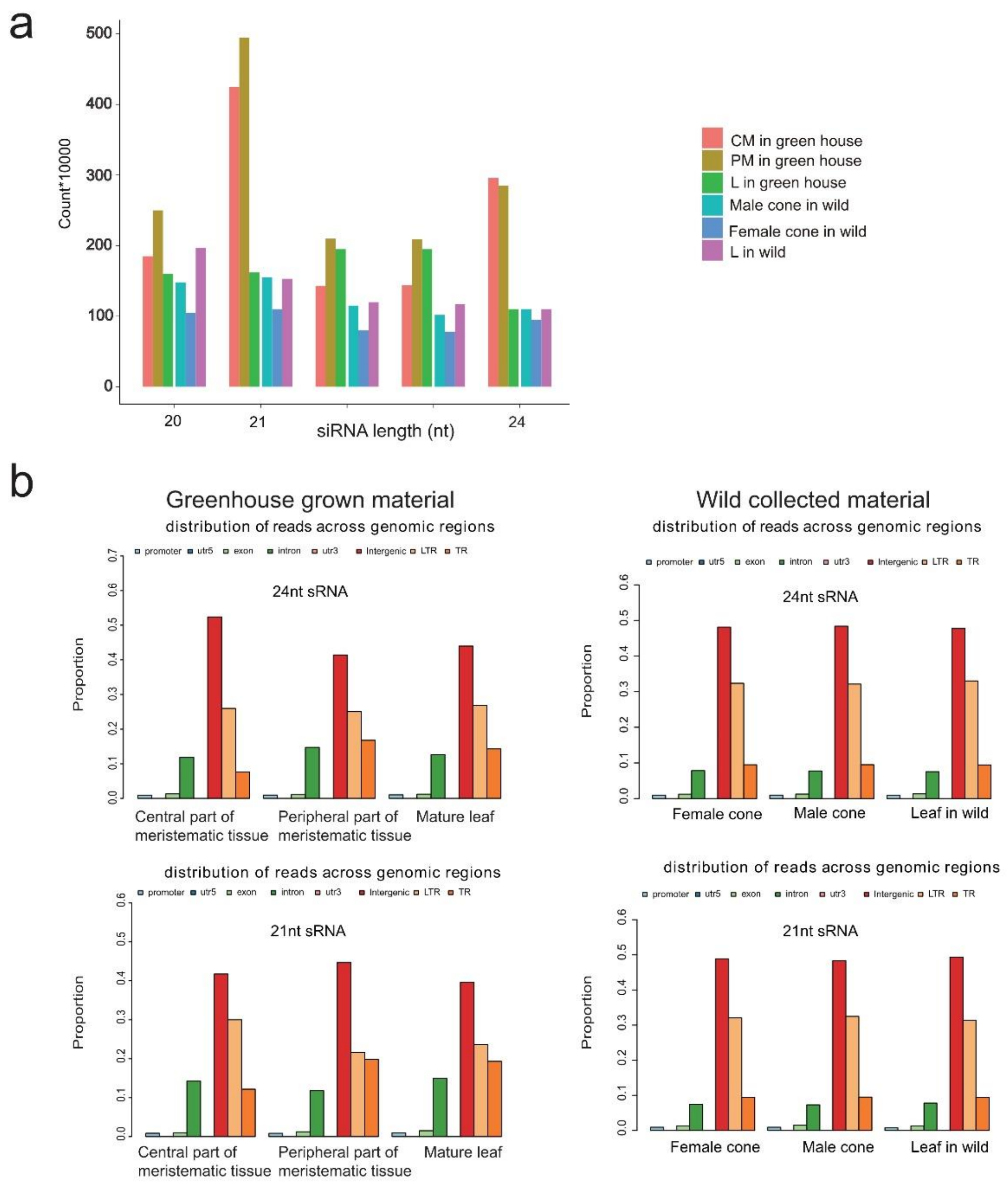

Supplementary Figure 5. The variation in biogenesis of small RNAs between tissues and distribution of 21 and 24 siRNA reads across genomic regions. **a** Small RNAs of 21nt and 24nt are especially abundant in meristematic tissues (the central region of the basal meristem (CM), the peripheral part of the basal meristem (PM) compared with leaf (L) and cone material). **b** The abundance of 21 and 24 small RNAs across different tissues and genomic regions (promoter, 5' untranslated region (utr5), exon, intron, 3' untranslated region (utr3) intergenic, long terminal repeat (LTR) and simple tandem repeat (SR)) from a glasshouse grown individual in Fairy Lake Botanic Garden (left) and wild-collected material from Namibia (right). Both the reads of 21 and 24 siRNAs were mainly mapped to the intergenic region, LTRs and tandem repeats (TR).

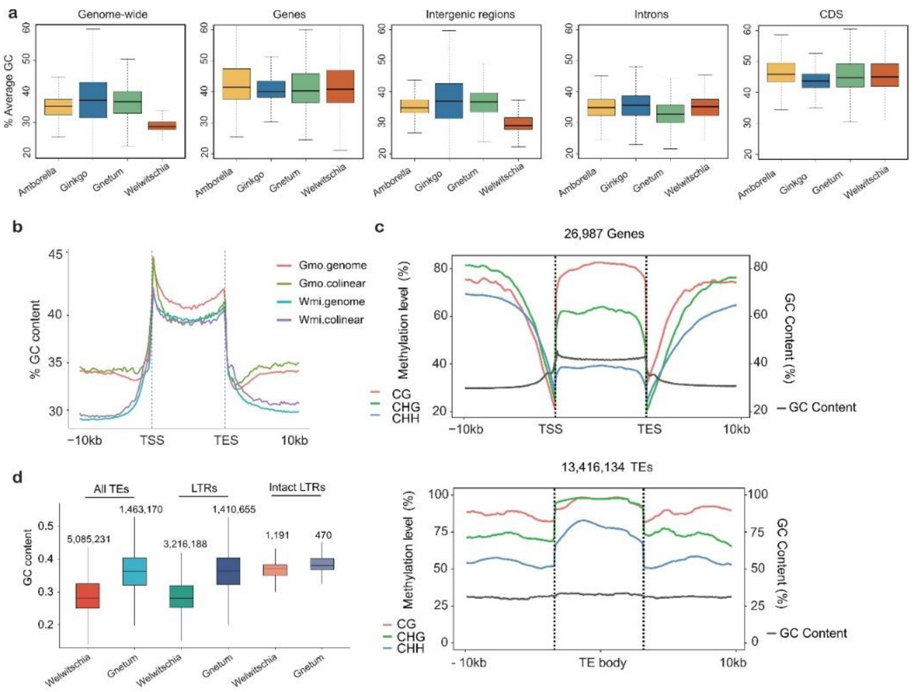

Supplementary Figure 6. The variation in GC content for selected plant genomes and DNA methylation patterns across genomic regions in *Welwitschia*. a For each case, the GC values were calculated in 500 kb windows with the number of separate window samples shown above each box plot (boxplot shows median value ± SD and whiskers represent maximum and minimum values). Boxplots of GC content across different genomic regions in *Amborella* (6,979 windows for genome-wide; 26,846 for genes; 31,977 for intergenic region; 82,937 for introns; 26,846 for CDS), *Gnetum* (129,709 windows for genome-wide; 27,392 for genes; 150,963 for intergenic region; 90,595 for introns; 27,419 for CDS), *Welwitschia* (13,744 windows for genome-wide; 27,010 for genes; 31,813 for intergenic region; 101,585 for introns; 26,990 for CDS) and *Ginkgo* (6,473,469 windows for genome-wide; 41,925 for genes; 6,500,395 for intergenic region; 136,090 for introns; 41,136 for CDS). **b** The GC content in genic and intergenic regions of *Gnetum* (Gmo) and *Welwitschia* (Wmi), including regions colinear between genomes show that the low levels of GC in intergenic regions of *Welwitschia* (Wmi.genome) are also GC poor in the regions in *Welwitschia* that are colinear with *Gnetum* (Wmi-colinear). Upstream, gene body and downstream regions around genes were divided into 100 proportionally sized bins. TSS – transcription start site; TES, transcription end site. **c** The methylation levels (left Y axis)) in three genomic contexts (CG, CHG, CHH) and GC content (right Y axis) are shown across coding genes (upper) and TE body (the protein coding regions) in *Welwitschia* (lower). In each case '−10 kb' indicates the upstream 10,000 bp and '10 kb' indicates the downstream 10,000 bp of the gene (TSS and TES respectively) or the start and end of the TE body divided into 82 proportionally sized bins. **d** Comparison of GC content between *Welwitschia* and *Gnetum* for all TEs, all LTR-RTs and intact LTR-RTs shows reduced GC level in incomplete LTR-RTs of *Welwitschia* compared with *Gnetum*. The number of elements analysed is shown above each box plot which gives median values (horizontal line) +/- SD, whiskers representing maximum and minimum values. Source data underling Supplementary Figure 6a and 6d are provided as a Source Data file.

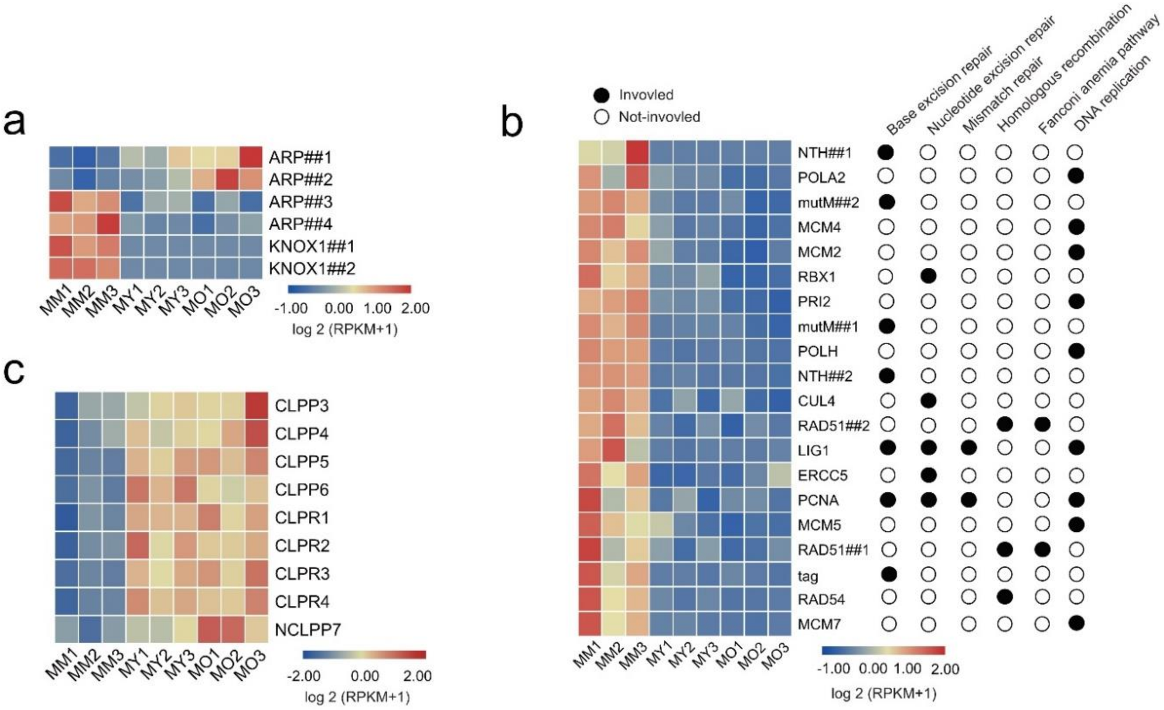

Supplementary Figure 7. Variation in expression patterns of specific genes among different tissue types. a The co-expression of *ARP3* and *ARP4* with *KNOX1-1* and *KNOX1-2* genes was observed in the basal meristem (MM1-3). **b** Twenty genes belonging to pathways functionally associated with DNA repair show differential expression between basal meristem compared with young (MY) and old (MO) leaf tissue. In general, these genes are upregulated in basal meristem tissues. **c** The continued expression pattern of *ClpP* genes is observed in young and old leaf sections of *Welwitschia*.

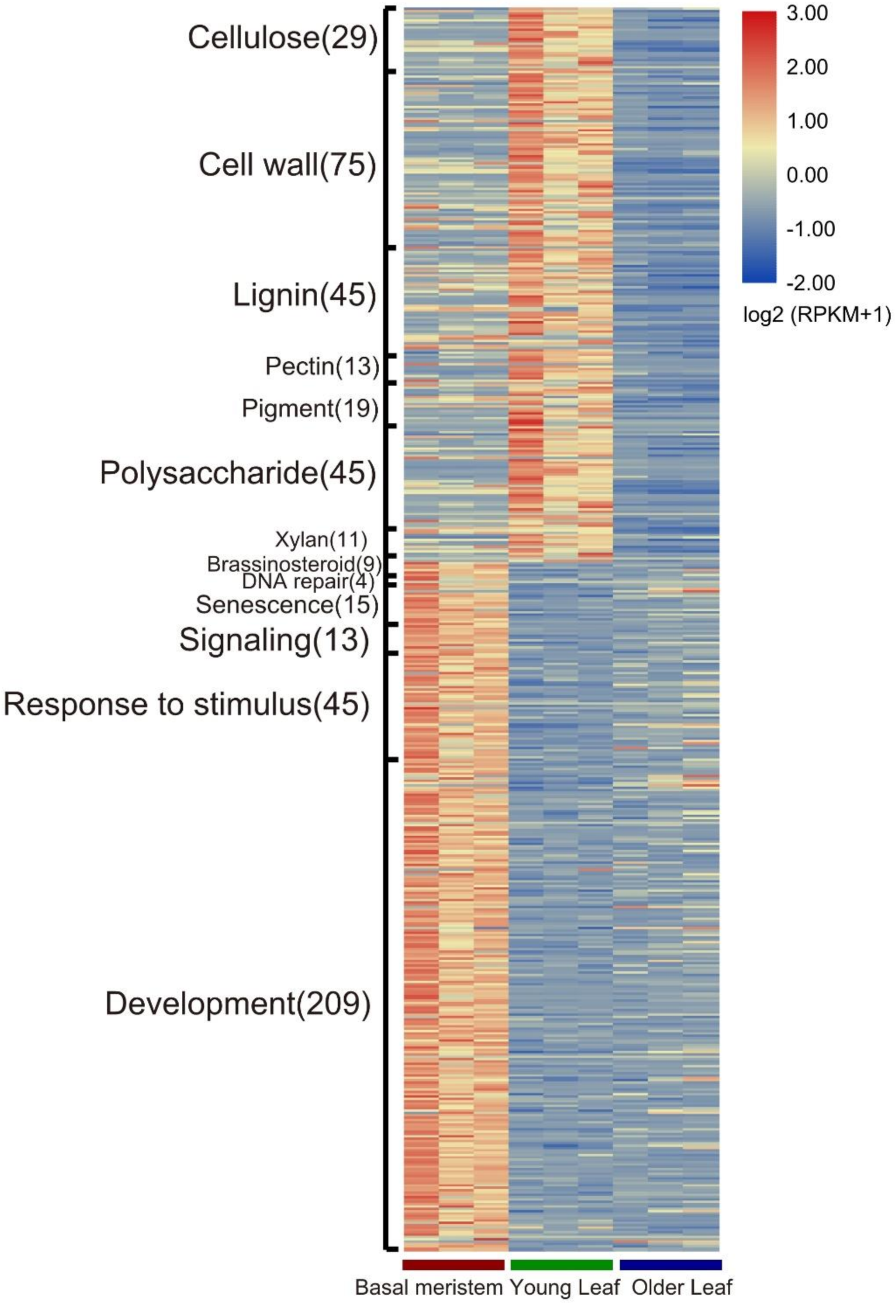

Supplementary Figure 8. Gene Ontology (GO) term enrichment for gene expression in basal meristems, young and old leaf sections. Heatmap showing differential gene expression patterns among basal meristems, young and old leaf sections. The number of genes in each category is indicated in brackets.

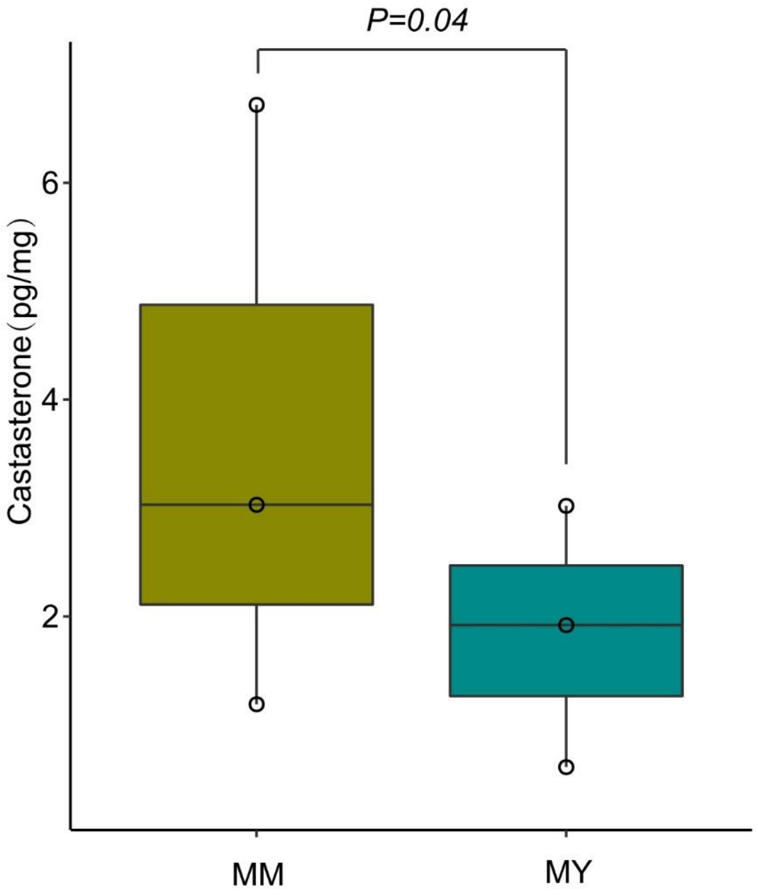

Supplementary Figure 9. Box plots showing the variation of absolute castasterone content between basal meristematic and young leaf (MY) tissue). n = 3 biologically independent samples for each group (boxplot shows median value +/- SD and whiskers represent maximum and minimum values), Fisher's exact test (two-sided analysis) was made for comparison, *P* = 0.04.

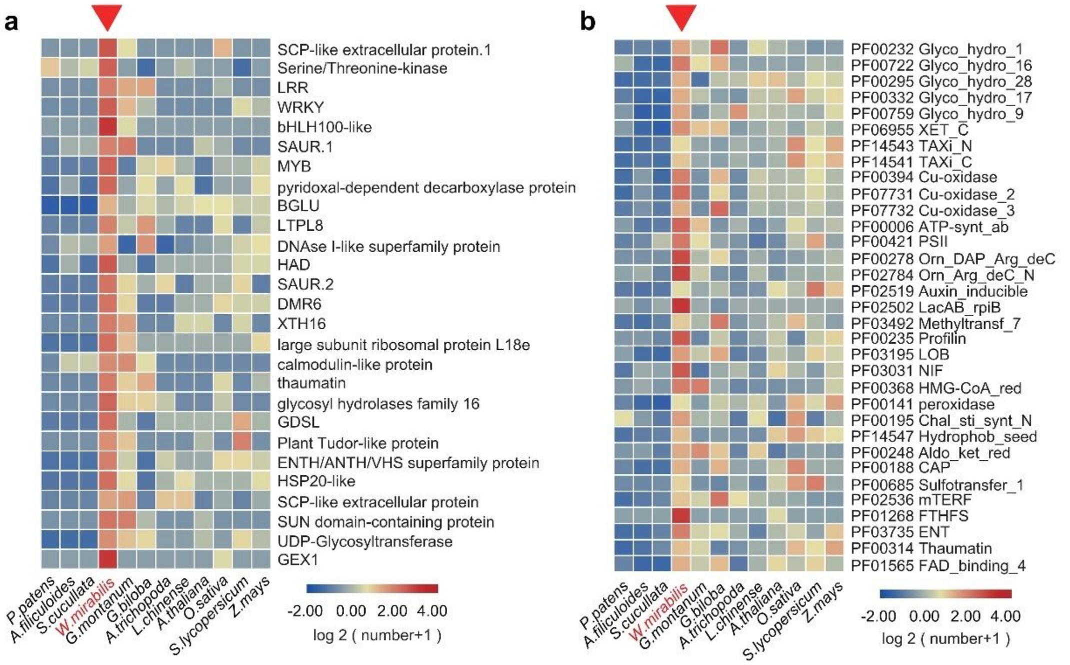

Supplementary Figure 10. Specific expansion of gene families and functional domains in *Welwitschia* compared with other land plants. a The heatmap shows categorized orthogroups which have significantly increased numbers in *Welwitschia* compared with other land plants analysed. **b** The pfam domain-based comparison highlights the probable number of functional genes in plants surveyed here.

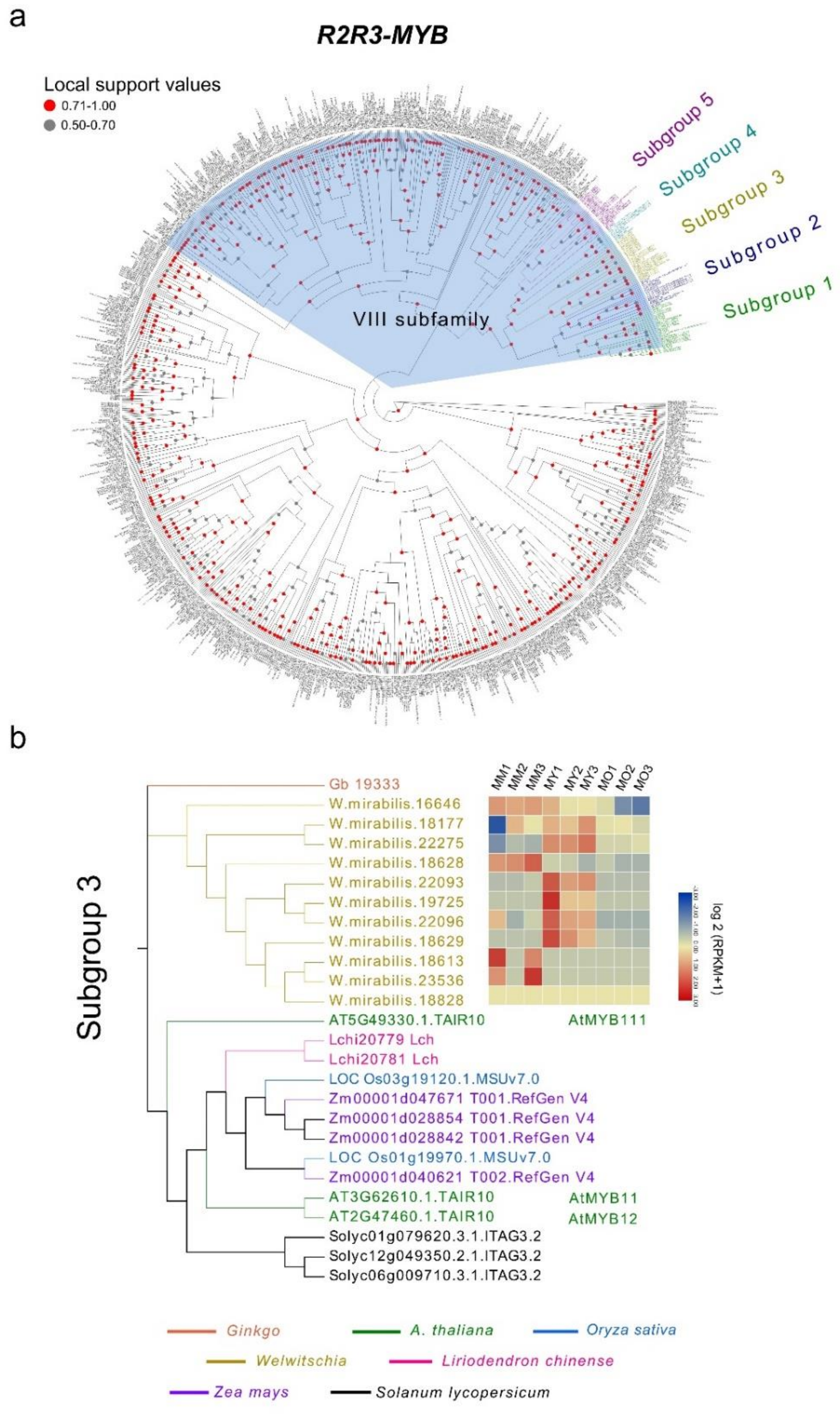

Supplementary Figure 11. Phylogenetic tree of *R2R3-MYB* genes using sequences from *Ginkgo, Welwitschia, Arabidopsis thaliana, Oryza sativa, Zea mays, Liriodendron chinense* and *Solanum lycopersicum*. a Subfamily VIII represents the most expanded clade in the *R2R3-MYB* family. The subgroups closely related to *AtMYB11* are highlighted in different colors. **b** *Welwitschia* showed specific expansion in subgroup 3 with gene expression differing between tissue types. MM1/2 - the basal meristem of a male individual, MY1/2/3 - a young leaf from a male individual (the newly emergent region), MO1/2/3 - old section of leaf of male individuals. Source data are provided as a Source Data file.

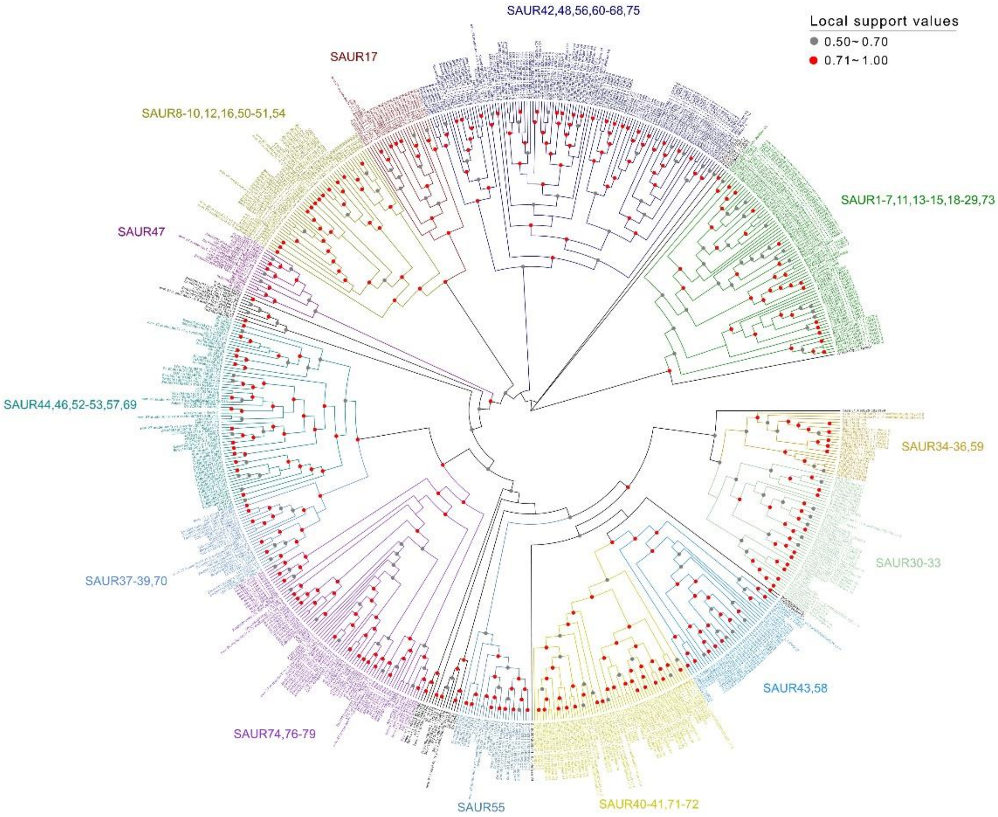

Supplementary Figure 12. Phylogenetic tree of the *SAUR* gene family. The tree was divided into 13 subfamilies according to the tree topology. Extensive expansion of genes was found in *SAUR* 17 and *SAUR*43,58 clades. Source data are provided as a Source Data file.

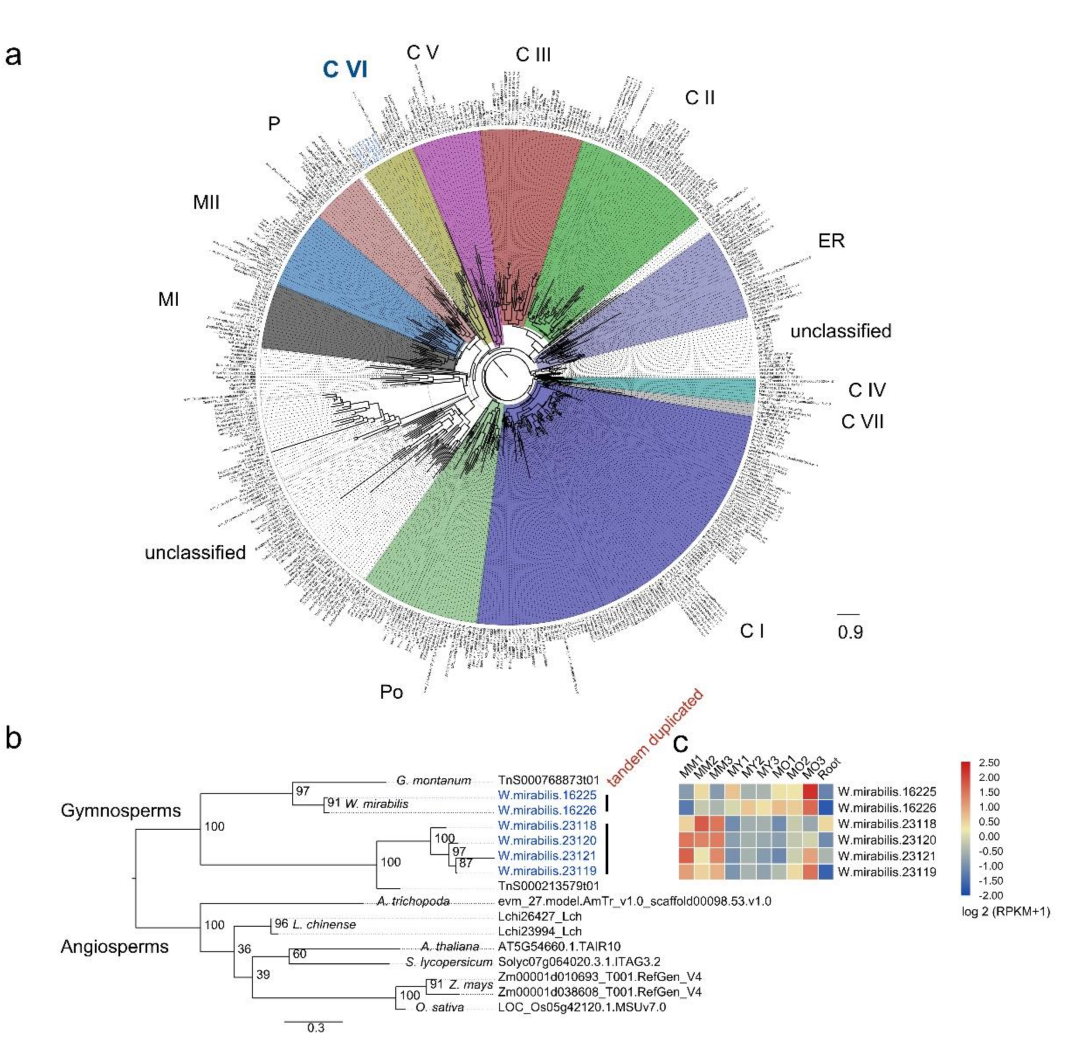

Supplementary Figure 13. **Phylogenetic tree of HSP20 proteins identified in representative seed plants**. **a** The analysis included three gymnosperms (*Gnetum montanum*, *Welwitschia*, *Ginkgo*), six angiosperms (*Amborella trichopoda*, *Liriodendron chinense*, *Solanum lycopersicum*, *Arabidopsis thaliana*, *Zea mays*, *Oryza sativa*). An unrooted neighbour-joining tree of 450 HSP20 proteins with clades representing distinctive subgroups of HSP20 proteins shown in different colours based on previous studies in *O. sativa*[58]. **b** The gene members of subgroup C VI are expanded in *Welwitschia* via tandem duplication. **c** Most of the paralogues of subgroup C VI in *Welwitschia* are highly expressed in the basal meristem. MM1/2 - the basal meristem of a male individual, MY1/2/3 - a young leaf from a male individual (the newly emergent region), MO1/2/3 - old section of leaf of male individuals, Root – male greenhouse grown individual. Source data underlying Figure 13a are provided as a Source Data file.

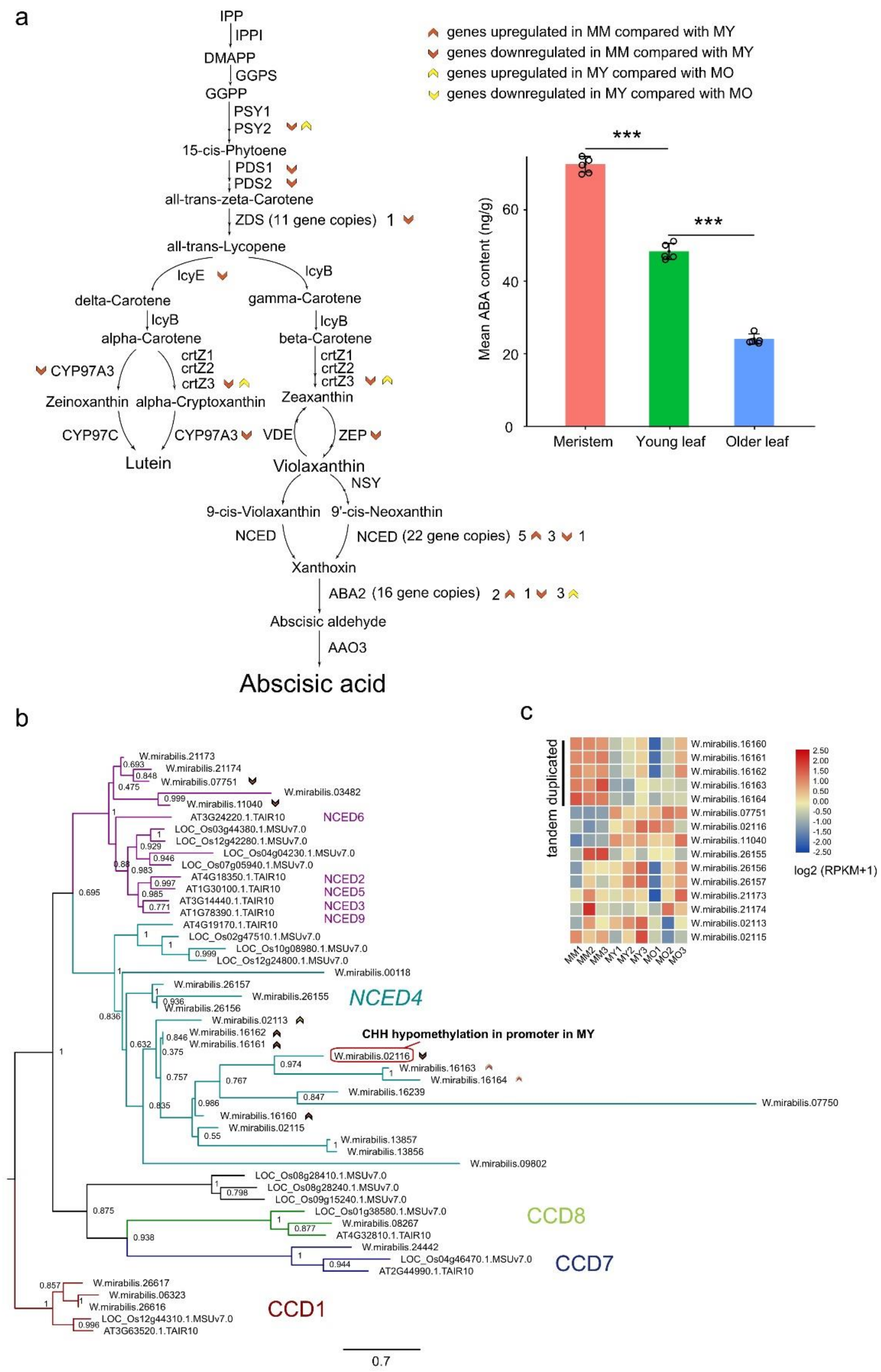

Supplementary Figure 14. ABA synthesis pathway in *Welwitschia* and enhanced activity of key genes in meristematic tissue and young leaf sections. a. Upward- and downward-pointing arrows indicate upregulated and downregulated genes, respectively (left). The different colours of the arrows represent different tissue comparisons. The copy numbers of *NCED* genes identified in *Welwitschia* are given, as are the number of genes with differential

expression. The concentration of ABA showed significant variation between tissues (right, Supplementary Data 7). n = 5 biologically independent samples for each group, mean ± SD; one-way analysis of variance (ANOVA): F = 752.8, P = 2.44 × 10-13; Tukey's honestly significant difference (HSD) post hoc test: ***, *P* < 0.001. MM - the basal meristem of a male individual, MY - young leaf from a male individual (the newly emergent region), MO - old section of leaf of male individuals. **b** A neighbour joining (NJ) tree showing well-categorized members of carotenoid cleavage dioxygenase gene family sequences from *Arabidopsis thaliana*, *Oryza sativa* and *Welwitschia*. Several clades are highlighted by branch colour. In the NCED4 clade, one gene family member from *Welwitschia* was hypomethylated at CHH sites in the promoter regions in young leaf compared to basal meristem tissue. **c** The varied expression level of *NCED* among three tissues. A clade of *NCED* genes expanded specifically in *Welwitschia* via tandem duplication were upregulated in the basal meristem. Source data underlying Supplementary Figure 14b are provided as a Source Data file.

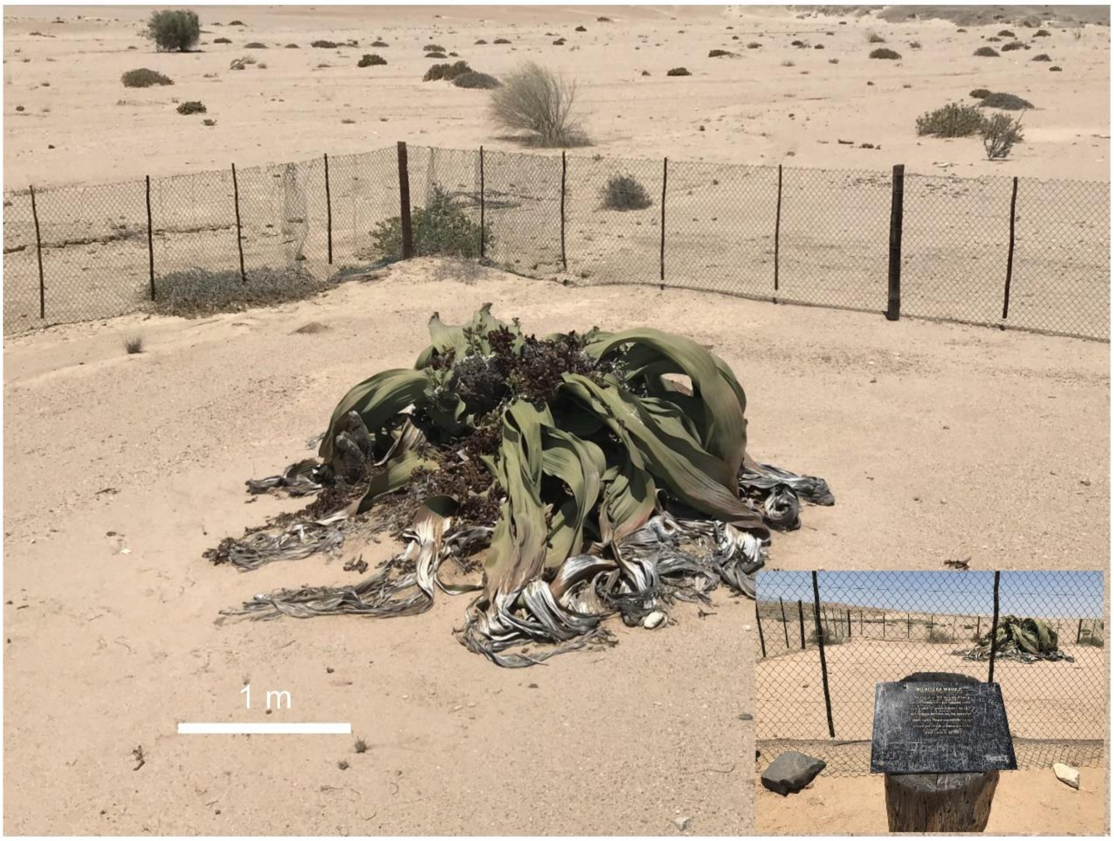

Supplementary Figure 15. An aged individual protected by the local government and the stone tablet (lower right corner) highlights its probable longevity of around 1,500 years. *Welwitschia* occurs on a diverse range of soils and rocky areas, including coarse alluvial sand in small riverbeds. The huge wide leaves split into strips that then grow at different rates and in different directions from the basal meristems.

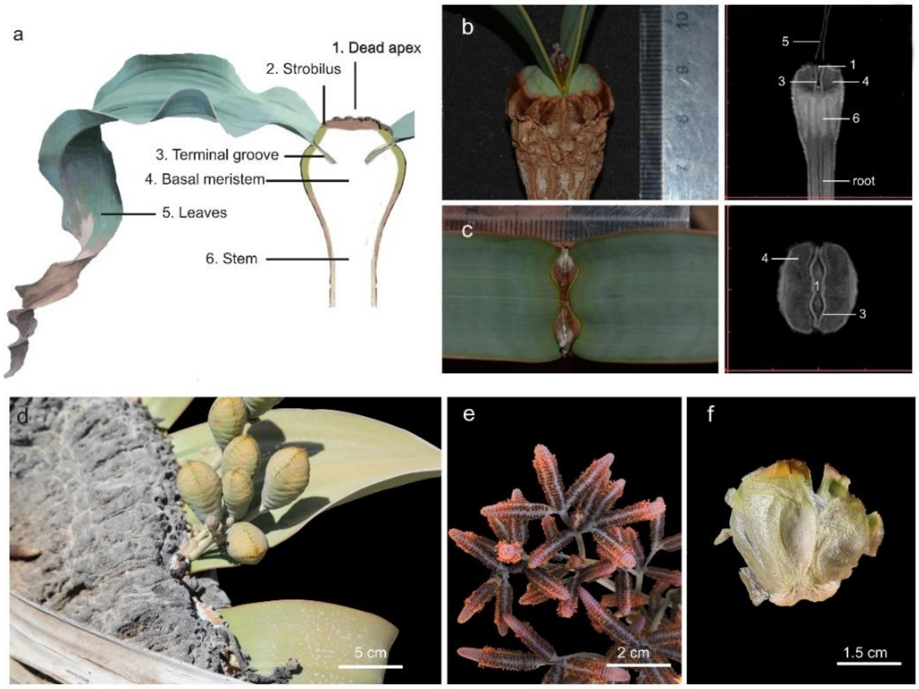

Supplementary Figure 16. Nuclear Magnetic Resonance Imaging (MRI) scan of *Welwitschia*. a Schematic image and some of the salient features of *Welwitschia* of a young plant showing the insertion of leaf and strobilus into the stem. The position where the strobilus forms is also indicated on the figure as '2. Strobilus'. **b** A young individual showing the two leaves growing from the 'basal meristem' (left). The corresponding MRI scan (right) shows the different density of the tissues. The stem, meristematic tissue and root can be easily identified, as can the scar body locate in the middle of the shoot apex (1 = dead apex, 2 = strobilus, 3 = terminal groove, 4 = basal meristem, 5 = leaf, 6 = stem). **c** Aerial view of *Welwitschia*, highlighting the leaves and scar body (left), while the corresponding scan (right) shows boundaries between the two leaves, meristematic tissue, scar body and the terminal groove. **d** The female cones are produced from the edge of the concave crown, see position '2 Strobilus' in (a). **e** The male cones are produced in the same way as the female cones. **f** *Welwitschia*'s seeds have lateral wings formed by the fusion of a pair of bracts and are probably dispersed by wind or water.

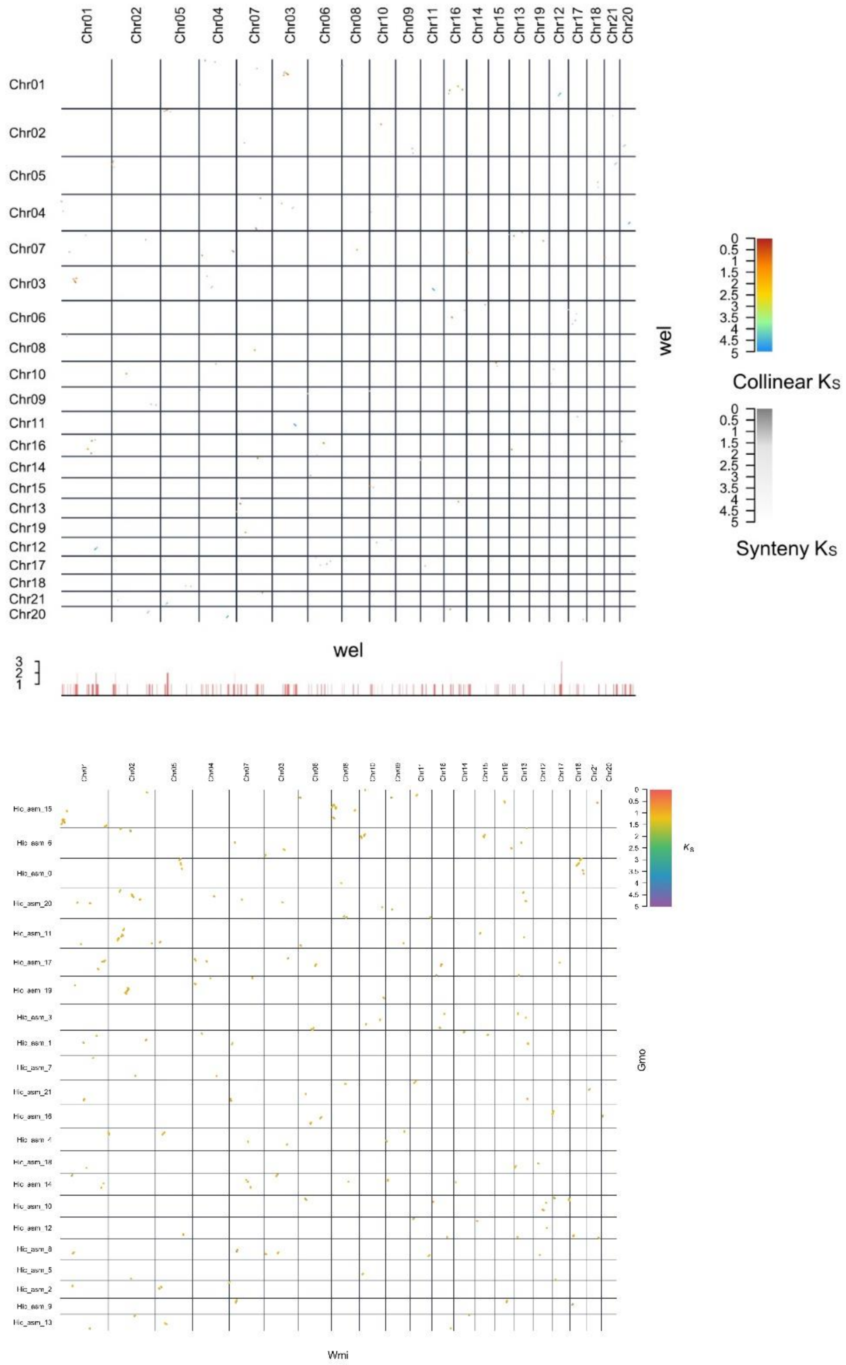

Supplementary Figure 17. Dot plot of inter-genomic comparison between *Gnetum* and *Welwitschia* and intra-genomic comparison of *Welwitschia*. Only collinear segments (genomic blocks with homologous genes in the same order; K_S values shown in a color scale) and syntenic segments (genomic blocks with homologous genes but not in the same order; K_S values shown in a grey scale) with at least four anchor pairs are shown. The red bars below the dot plot illustrate the duplication depths (the number of connected collinear and syntenic segments overlapping at each position). Many of these collinear and syntenic regions in the *Welwitschia* genome are covered by their paralogous segments once, while a few of them may have up to three paralogous segments in the genome, indicating the possibility of a more ancient large-scale duplication event[29-31] (upper). Likewise, the collinear segments between *Gnetum* and *Welwitschia* were displayed (lower)

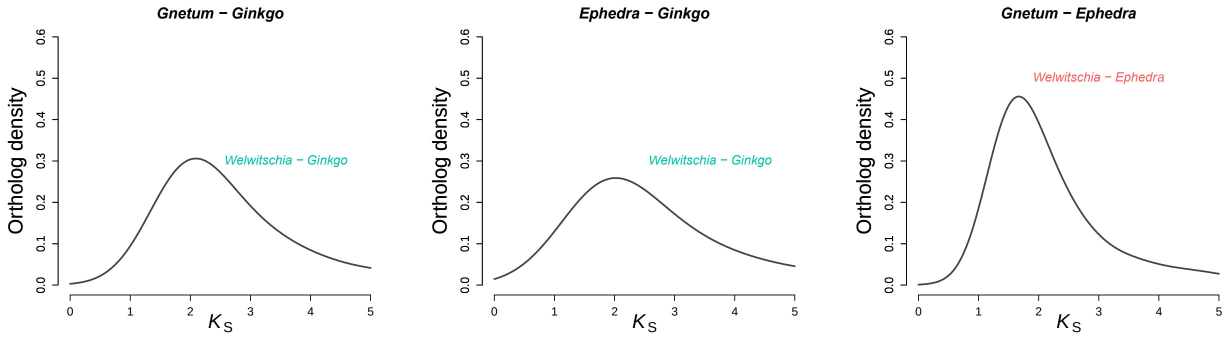

Supplementary Figure 18. Distributions of K_S of one-to-one orthologs identified between Ginkgo and three gnetophyte species and between Ephedra and the other two gnetophyte species. In each plot, the kernel density estimation (KDE) of an orthologous K_S distribution between *Welwitschia* and *Ginkgo* (green) or *Ephedra* (red) is compared with the KDE of the orthologous K_S distribution between the other two gnetophyte species with *Ginkgo* or *Ephedra* (dark grey line), respectively.

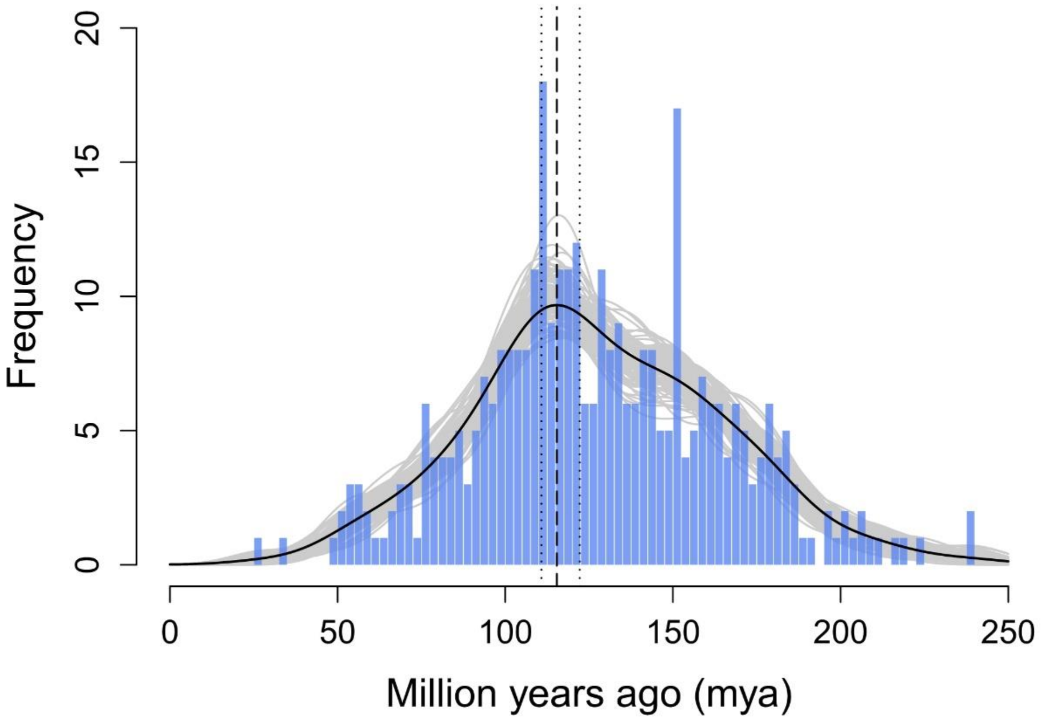

Supplementary Figure 19. Absolute age of the *Welwitschia* WGD event. Absolute age distribution obtained by phylogenomic dating of Welwitschia paralogs. The solid black line represents the kernel density estimates (KDE) of the dated paralogs, and the vertical dashed black lines represents its peak (mode) at 115 mya, which was used as the consensus WGD age estimate. The grey lines represent density estimates from 2,500 bootstrap replicates and the vertical black dotted lines represent the corresponding 90% confidence interval for the WGD age estimate, 111 – 122 Ma. The histogram shows the raw distribution of dated paralogs.

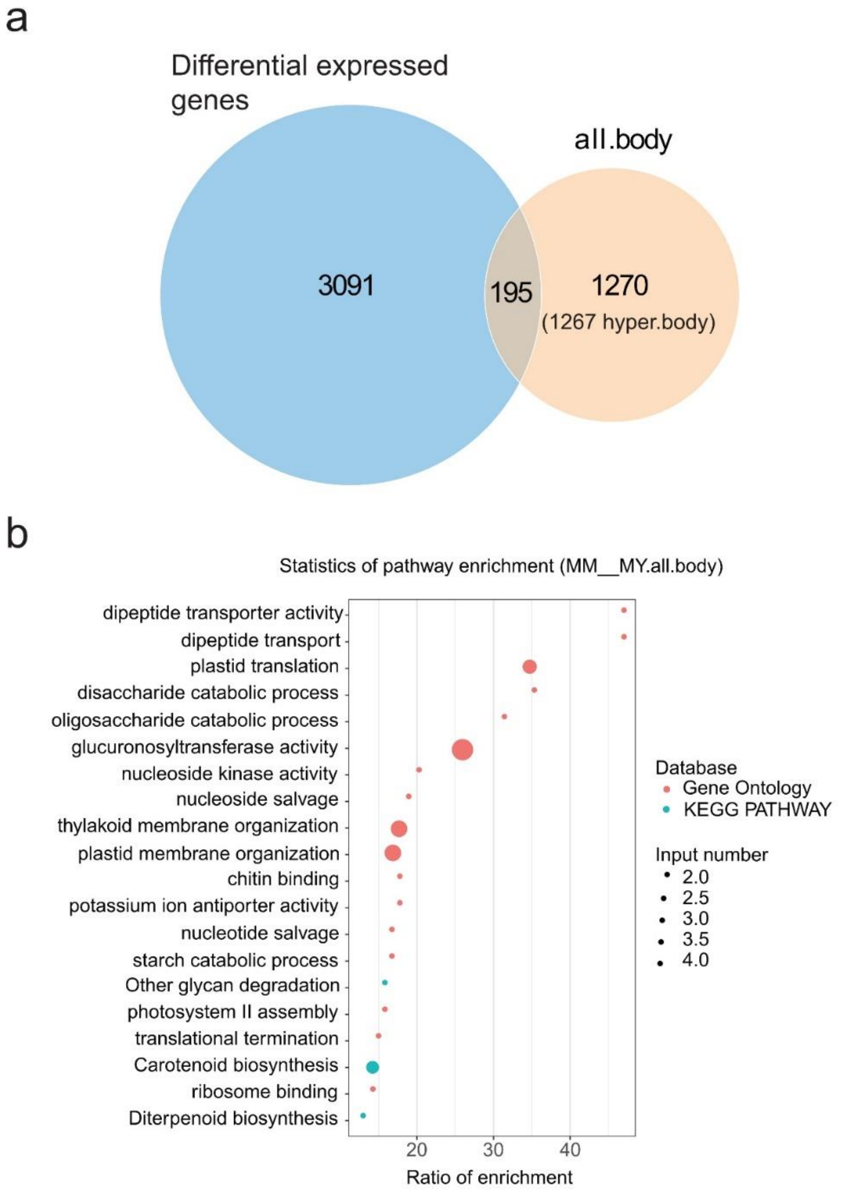

Supplementary Figure 20. **Pathway enrichment analysis of genes identified to be both differentially methylated and differentially expressed in male basal meristems (MM) and young male leaves (MY**). **A** 195 genes identified that are both differentially methylated and differentially expressed. Mostly hypermethylation in the gene body occurred in MM. **b** The enriched pathways correspond to the genes showing elevated expression level in MY.

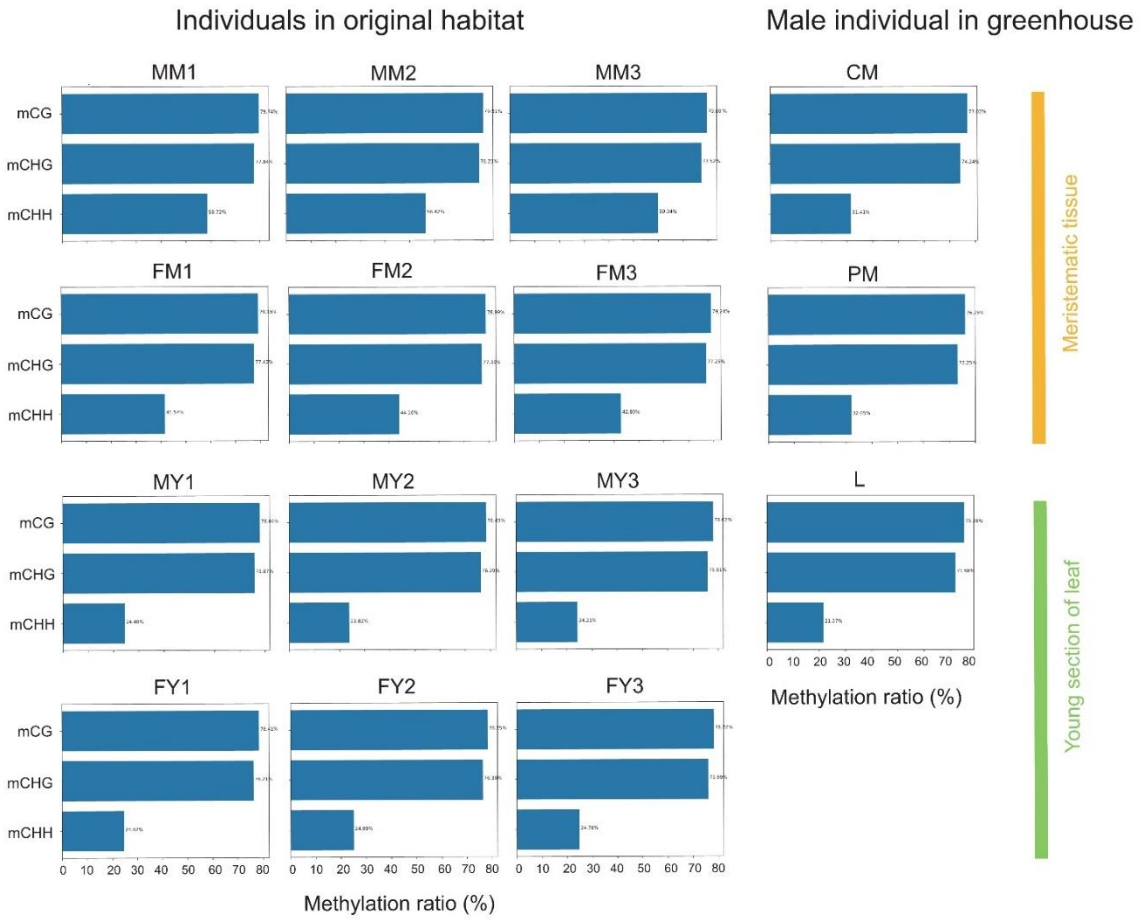

Supplementary Figure 21. Global average DNA methylation ratios of CG, CHG and CHH in different replicates of leaf and meristematic tissue from male and female individuals. The CG and CHG methylation ratios are both high and stable in all samples (close to 80% of all cytosines). CHH methylation ratio in leaves showed little variation (~25% of all cytosines) between individuals. In contrast, there was considerable variation between tissue types, sexes, and wild-collected and greenhouse-grown individuals.

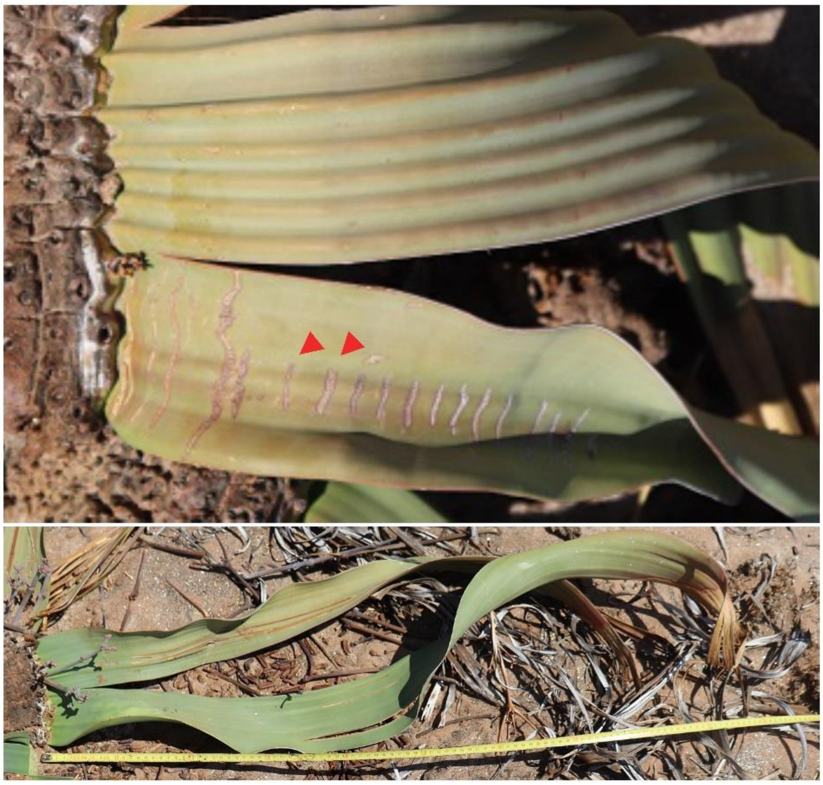

Supplementary Figure 22. Monthly measurement of the leaf growth. The red triangles (upper image) show scratch marks used to estimate the growth rate of *Welwitschia*'s leaf. The internal length of leaf sections sampled were measured to predict the ages of the leaves (lower image).

Supplementary Table 1. Statistics of *W. mirabilis* and *G. montanum* genome assemblies.

		W.mirabilis	*G.montanum*
Contig Assembly	Total Number of Contigs	15,330	483,454
	Assembly size (bp)	6,865,481,713	3,868,026,537
	N50 (bp)	1,481,633	24,977
	N90 (bp)	287,630	3,785
	Largest Contig (bp)	12,794,740	381,306
Scaffold Assembly	Total Number of Scaffolds	7,156	122,369
	Assembly size (bp)	6,866,294,267	4,132,221,696
	N50 (bp)	295,495,320	156,939,799
	N90 (bp)	188,211,703	284,780
	Largest Scaffold (bp)	551,969,684	228,294,350
	Chromosome size (bp)	6,430,884,056	3,573,235,772
	Chromosome mount rate (%)	93.65	86.47
Assessment	GC content (%)	29.07	37.25
	Repeat density (%)	86.85	85.93
	Mapped reads (%)	99.00	99.83
	Polymorphism rate (%)	0.59	0.76
	BUSCO (%)*	83.47	84.60
Annotation	Number of protein-coding genes	26,990	27,354
	% of genes with InterPro domain	70.06	72.12
	% of genes with GO terms	48.24	52.60
	Mean gene length (bp)	25,949.11	7,386.40
	Mean exon length (bp)	216.36	231.36
	Mean intron length (bp)	6,620.19	1,784.31

* Protein sequence were used for BUSCO

Supplementary Table 2. Data sources used in this study.

Group	Species	Abbreviation	Database	Source
Bryophyta	*Physcomitrella patens*	Ppa	Ensembl	ftp://ftp.ensemblgenomes.org/pub/release-25/plants/
Pteridophyta	*Salvinia cucullata*	Scu	Fernbase	ftp://ftp.fernbase.org/
	Azolla filiculoides	Afi	Fernbase	ftp://ftp.fernbase.org/
	Ceratopteris gametophytes(RNA)	Cga	Dryad	https://doi.org/10.5061/dryad.f7f57
	Lygodium japonicum (RNA)	Lja	Dryad	https://doi.org/10.5061/dryad.f7f57
	Pteridium aquilinum (RNA)	Paq	Dryad	https://doi.org/10.5061/dryad.f7f57
Gnetales	*Gnetum montanum*	Gmo	Dryad	https://datadryad.org/stash/dataset/doi:10.5061/dryad.0vm37
	Gnetum montanum (RNA)	Gne	Dryad	https://doi.org/10.5061/dryad.f7f57
	Welwitschia mirabilis(RNA)	Wel	Dryad	https://doi.org/10.5061/dryad.f7f57
	Ephedra equisetina	Eeq	Dryad	https://datadryad.org/stash/dataset/doi:10.5061/dryad.0vm37
	Ephedra equisetina (RNA)	Eph	Dryad	https://doi.org/10.5061/dryad.f7f57
Cycadales + Ginkgoaceae	*Ginkgo biloba*	Gbi	GigaDB	http://gigadb.org/dataset/100209
	Ginkgo_biloba (RNA)	Gin	Dryad	https://doi.org/10.5061/dryad.f7f57
	Cyca revoluta (RNA)	Cre	Dryad	https://doi.org/10.5061/dryad.f7f57
	Zamia furfuracea (RNA)	Zma	Dryad	https://doi.org/10.5061/dryad.f7f57
Pinaceae	*Picea abies*	Pab	ConGenIE	http://congenie.org/
	Picea abies (RNA)	Pic	Dryad	https://doi.org/10.5061/dryad.f7f57
	Pinus taeda	Pta	ConGenIE	http://congenie.org/
	Pinus taeda (RNA)	Pit	Dryad	https://doi.org/10.5061/dryad.f7f57
	Abies firma (RNA)	Abi	Dryad	https://doi.org/10.5061/dryad.f7f57
Conifer II	*Araucaria cunninghamii (RNA)*	Ara	Dryad	https://doi.org/10.5061/dryad.f7f57

	Cephalotaxus sinensis (RNA)	Cep	Dryad	https://doi.org/10.5061/dryad.f7f57
	Metasequoia glyptostroboides (RNA)	Met	Dryad	https://doi.org/10.5061/dryad.f7f57
	Podocarpus macrophyllus (RNA)	Pod	Dryad	https://doi.org/10.5061/dryad.f7f57
	Sciadopitys verticillata (RNA)	Sci	Dryad	https://doi.org/10.5061/dryad.f7f57
	Taxus chinensis (RNA)	Tax	Dryad	https://doi.org/10.5061/dryad.f7f57
Basal angiosperms	*Amborella trichopoda*	Atr	Ensembl	ftp://ftp.ensemblgenomes.org/pub/plants/release-25/plants/
	Liriodendron chinense	Lch	NCBI	https://www.ncbi.nlm.nih.gov/assembly/GCA_003013855.2
Eudicots	*Arabidopsis thaliana*	Ath	JGI	https://phytozome.jgi.doe.gov/pz/portal.html
	Solanum lycopersicum	Sly	JGI	https://phytozome.jgi.doe.gov/pz/portal.html
	Populus trichocarpa	Ptr	JGI	https://phytozome.jgi.doe.gov/pz/portal.html
	Vitis vinifera	Vvi	JGI	https://phytozome.jgi.doe.gov/pz/portal.html
Monocots	*Ananas comosus*	Aco	JGI	https://phytozome.jgi.doe.gov/pz/portal.html
	Oryza sativa	Osa	MSU	http://rice.plantbiology.msu.edu/
	Zea_mays	Zma	JGI	https://phytozome.jgi.doe.gov/pz/portal.html
	Musa acuminata	Mac	JGI	https://phytozome.jgi.doe.gov/pz/portal.html

Supplementary Table 3. Repetitive sequences distribution along the chromosomes of *Welwitschia* and *Gnetum*.

Species	Chromosome	Length(bp)	Tandem repeat		LTR	
			Repeat size(bp)	% of chromo-some	Repeat size(bp)	% of chromo-some
Welwitschia	Chr01	551,969,684	143,013,865	25.91	309,159,878	56.01
	Chr02	534,277,133	139,612,528	26.13	290,406,672	54.36
	Chr03	440,121,154	115,266,287	26.19	247,455,422	56.22
	Chr04	398,342,641	103,702,704	26.03	220,405,456	55.33
	Chr05	384,200,962	97,464,471	25.37	208,839,019	54.36
	Chr06	368,624,366	95,880,469	26.01	205,087,801	55.64
	Chr07	359,756,438	91,441,428	25.42	194,890,620	54.17
	Chr08	323,699,550	83,597,687	25.83	178,602,869	55.18
	Chr09	295,495,320	78,378,491	26.52	164,040,964	55.51
	Chr10	275,933,556	73,206,828	26.53	151,864,066	55.04
	Chr11	270,531,590	70,221,196	25.96	150,458,689	55.62
	Chr12	262,840,185	72,015,579	27.40	148,379,919	56.45
	Chr13	262,343,103	70,157,593	26.74	149,304,419	56.91
	Chr14	251,350,775	65,493,471	26.06	139,748,296	55.60
	Chr15	246,406,728	64,790,532	26.29	138,132,022	56.06
	Chr16	231,872,730	59,362,700	25.60	125,898,596	54.30
	Chr17	224,340,954	59,227,431	26.40	123,062,383	54.86
	Chr18	204,201,206	53,261,880	26.08	113,964,444	55.81
	Chr19	202,044,856	52,062,439	25.77	109,235,814	54.07
	Chr20	188,211,703	49,557,078	26.33	102,225,940	54.31
	Chr21	154,319,422	40,534,531	26.27	83,132,597	53.87
	ContigUN	436,123,611	91,814,096	21.05	240,440,600	55.13
	Total	6,867,007,667	1,770,063,284	25.78	3,794,736,486	55.26
Gnetum	Hic_1	228,294,350	5,330,531	2.33	143,095,904	62.68
	Hic_2	155,796,758	3,282,193	2.11	106,022,801	68.05
	Hic_3	151,286,287	3,387,788	2.24	99,482,508	65.76
	Hic_4	155,722,635	3,470,301	2.23	101,813,849	65.38
	Hic_5	162,095,710	3,504,256	2.16	106,569,647	65.74
	Hic_6	151,434,850	3,412,730	2.25	99,057,234	65.41
	Hic_7	163,104,109	4,216,604	2.59	109,992,635	67.44
	Hic_8	142,617,651	3,422,944	2.40	92,535,359	64.88
	Hic_9	134,605,858	2,810,012	2.09	88,139,164	65.48
	Hic_10	122,874,075	2,642,610	2.15	76,472,108	62.24
	Hic_11	143,781,008	3,093,042	2.15	93,695,437	65.17
	Hic_12	209,211,847	4,502,073	2.15	139,630,632	66.74
	Hic_13	117,687,481	2,741,318	2.33	77,150,642	65.56

Hic_14	124,313,964	2,680,793	2.16	78,743,325	63.34
Hic_15	184,043,878	3,935,295	2.14	120,119,826	65.27
Hic_16	200,177,288	4,374,437	2.19	135,023,972	67.45
Hic_17	188,787,460	4,160,572	2.20	123,431,114	65.38
Hic_18	180,025,510	3,921,636	2.18	119,884,140	66.59
Hic_19	163,567,625	3,574,186	2.19	107,038,956	65.44
Hic_20	172,814,875	4,472,759	2.59	114,962,526	66.52
Hic_21	164,052,754	3,967,139	2.42	110,280,845	67.22
Hic_22	156,939,799	3,328,787	2.12	100,947,234	64.32
UN	558,985,924	14,723,051	2.63	140,318,383	25.10
Total	4,132,221,696	94,955,057	2.26	2,484,408,241	63.79

Supplementary Table 4. Prediction of the repetitive elements in *Welwitschia*.

Type		Repeat size(bp)	% of genome
TRF[a]		1,770,063,284	25.78
RepeatProteinMask[b]		1,215,427,956	17.70
RepeatMasker+Homology+Denovo[c]	DNA	207,531,001	3.02
	LINE	300,532,832	4.38
	SINE	2,576,780	0.04
	LTR	3,794,736,486	55.26
	Other	80,019,750	1.17
	Unknown	2,216,786,041	32.28
Total[d]		6,052,188,457	88.13

a: TRF was used to predict tandem repeats (SR).

b: RepeatProteinMask was used to identify repeats in genome according to homology to identified repeat elements in Repbase.

c: RepeatMasker was firstly used to identify repeats in the genome according results of RepeatScout, LTR-FINDER, Piler-DF and RepeatModeler. Then according to homology to identified repeat elements in Repbase.

d: Total stat of all repetitive elements in *Welwitschia mirabilis*.

Supplementary Table 5. The historical activity of major elements of LTRs in *Welwitschia.*

Time (mya)	Categories of LTR	Copies in *Welwitschia*
0-5	Ty1-*copia*	13,893
	Ty3-*gypsy*	9,999
	Non-autonomous	10,589
10-15	Ty1-*copia*	2,045
	Ty3-*gypsy*	2,690
	Non-autonomous	746
All	Ty1-*copia*	40,344
	Ty3-*gypsy*	27,672
	Non-autonomous	21,134

Supplementary Table 6. Identification of the reverse transcriptase genes from complete retrotransposons (containing all expected protein coding domains).

	Welwitschia	*Gnetum*	*Ginkgo*	*Amborella*
Ty1-*copia*	378	115	1707	24
Ty3-*gypsy*	131	618	2530	19

Supplementary Table 7. Information of DNA data collection and type of samples in this study.

Type of samples	Species	Experiment	Information[a]	Clean data(Gbp)	Sequence depth[b]
DNA	*Welwitschia*	Illumina	Leaf	936.21	133.74
		Nanopore	Leaf	758.35	108.34
		Hi-C	Leaf	807.78	115.40
	Gnetum	10X Genomics	Leaf	381.46	92.81
		BioNano	Leaf	395.44	96.21
		Hi-C	Leaf	587.90	143.04

a: MM is short for basal meristem from male individual. FM is short for basal meristem from female individual. MY is short for young leaf from male individual. FY is short for young leaf from female individual. CM is short for central region of the basal meristem from male individual in greenhouse. PM is short for peripheral part of the basal meristem from male individual in greenhouse.

b: *G. montanum's* genome size (4.11Gb/1C) and *W. mirabilis's* genome size (7Gb/1C) were estimated from k-mer analysis.

Supplementary Table 8. Information of RNA sequencing data collection and type of samples in this study.

Type of samples	Experiment	Information[a]	Clean data (Gbp)	Sequence depth[b]	Mapping rate (%)
RNA	Illumina	Root	7.02	1.00	72.00
		Leaf	7.56	1.08	77.40
		Male cone	6.55	0.94	78.20
		Basal meristem from male individual (MM1)	7.24	1.03	75.50
		Basal meristem from male individual (MM2)	8.12	1.16	76.20
		Basal meristem from male individual (MM3)	7.28	1.04	76.60
		Basal meristem from female individual (FM1)	6.72	0.96	75.30
		Basal meristem from female individual (FM2)	6.73	0.96	75.60
		Basal meristem from female individual (FM3)	7.72	1.10	75.10
		Young leaf from male individual (MY1)	6.58	0.94	76.40
		Young leaf from male individual (MY2)	7.28	1.04	76.00
		Young leaf from male individual (MY3)	7.23	1.03	76.40
		Young leaf from female individual (FY1)	7.21	1.03	76.80
		Young leaf from female individual (FY2)	6.42	0.92	76.50
		Young leaf from female individual (FY3)	6.40	0.91	76.50
		Older leaf from male individual (MO1)	9.09	1.30	76.30
		Older leaf from male individual (MO2)	9.08	1.30	76.40
		Older leaf from male individual (MO3)	10.02	1.43	76.30
		Older leaf from female individual (FO1)	7.63	1.09	76.60
		Older leaf from female individual (FO2)	7.83	1.12	76.40
		Older leaf from female individual (FO3)	8.94	1.28	76.00

Central region of the basal meristem from male individual in greenhouse (CM)	5.56	0.79	71.50
Peripheral part of the basal meristem from male individual in greenhouse (PM)	5.84	0.83	71.10
Leaf from male individual in greenhouse (L)	4.08	0.58	78.20

a: MM is short for basal meristem from male individual. FM is short for basal meristem from female individual. MY is short for young leaf from male individual. FY is short for young leaf from female individual. CM is short for central region of the basal meristem from male individual in greenhouse. PM is short for peripheral part of the basal meristem from male individual in greenhouse.

b: *G.montanum's* genome size (4.11Gb/1C) and *W. mirabilis's* genome size (7Gb/1C) were estimated from k-mer analysis.

Supplementary Table 9. Information of methylome data collection and type of samples in this study.

Type of samples	Experiment	Information[a]	Clean data (Gbp)	Sequence depth[b]	Mapping rate (%)	Coverage (%)
Bisulfite sequencing	Illumina	Basal meristem from male individual in wild (MM1)	100.96	14.42	73.28	75.00
		Basal meristem from male individual in wild (MM2)	100.12	14.30	72.98	74.89
		Basal meristem from male individual in wild (MM3)	94.20	13.46	72.87	74.18
		Basal meristem from female individual in wild (FM1)	103.47	14.78	72.89	75.32
		Basal meristem from female individual in wild (FM2)	102.25	14.61	72.87	75.35
		Basal meristem from female individual in wild (FM3)	89.26	12.75	73.38	74.19
		Young leaf from male individual in wild (MY1)	100.49	14.36	72.54	74.59
		Young leaf from male individual in wild (MY2)	105.08	15.01	72.30	74.14
		Young leaf from male individual in wild (MY3)	103.54	14.79	73.02	75.19
		Young leaf from female individual in wild (FY1)	102.90	14.70	72.20	75.05
		Young leaf from female individual in wild (FY2)	97.58	13.94	71.73	74.04
		Young leaf from female individual in wild (FY3)	100.56	14.37	72.33	75.25
		Central region of the basal meristem from male individual in greenhouse (CM)	118.66	16.95	70.71	76.40
		Peripheral part of the basal meristem from male individual in greenhouse (PM)	110.06	15.72	70.49	75.95
		Leaf from male individual in greenhouse (L)	98.82	14.12	69.70	74.32

a: MM is short for basal meristem from male individual. FM is short for basal meristem from female individual. MY is short for young leaf from male individual. FY is short for young leaf

from female individual. CM is short for central region of the basal meristem from male individual in greenhouse. PM is short for peripheral part of the basal meristem from male individual in greenhouse.

b: *G.montanum's* genome size (4.11Gb/1C) and *W. mirabilis's* genome size (7Gb/1C) were estimated from k-mer analysis.

Supplementary Table 10. The significant variation on methylation of CHH context and location in TE region.

Groups[a]		In whole genome		In TEs region	
		Number	Percent (%)	Number	Percent (%)
MM-MY	Promoter	1,402	0.31	1,022	72.90
	UTR5	-	-	-	-
	Exon	562	0.12	347	61.74
	Intron	11,180	2.44	9,863	88.22
	UTR3	-	-	-	-
	Intergenic	445,401	97.13	396,781	89.08
FM-FY	Promoter	56	0.67	45	80.36
	UTR5	-	-	-	-
	Exon	41	0.49	31	75.61
	Intron	424	5.06	384	90.57
	UTR3	-	-	-	-
	Intergenic	7,854	93.78	7,063	89.93
MM-FM	Promoter	126	4.12	75	59.52
	UTR5	-	-	-	-
	Exon	107	3.05	57	53.27
	Intron	205	6.71	110	53.66
	UTR3	-	-	-	-
	Intergenic	2,617	85.66	1,448	55.33
MY-FY	Promoter	5	3.03	2	40.00
	UTR5	-	-	-	-
	Exon	11	6.67	2	18.18
	Intron	7	4.27	5	71.43
	UTR3	-	-	-	-
	Intergenic	142	86.06	78	54.93

a: MM = basal meristem from male individual. FM = basal meristem from female individual. MY = young leaf from male individual. FY = young leaf from female individual.

Supplementary Table 11. The numbers of DMRs in all three methylation contexts (CHH, CG and CHG) in different tissues, individuals and different genomic regions.

Context	Group	Type	Promoter	utr5	Exon	Intron	utr3	Intergenic
CHH	PM__L	hyper	2270	0	595	17237	0	648522
	PM__L	hypo	36	0	7	192	0	6449
	MM__MY	hyper	1402	0	562	11180	0	445400
	MM__MY	hypo	0	0	0	0	0	1
CHG	PM__L	hyper	124	0	313	2739	0	2703
	PM__L	hypo	308	0	207	678	0	1615
	MM__MY	hyper	18	0	37	198	0	2102
	MM__MY	hypo	173	0	50	231	0	1172
CG	PM__L	hyper	88	0	182	206	0	761
	PM__L	hypo	264	0	233	250	0	974
	MM__MY	hyper	3	0	7	8	0	60
	MM__MY	u	158	0	39	84	0	735

PM_L = peripheral part of basal meristem compared with mature leaf; MM_MY = male meristem compared with male young leaf. ‘hypo’ and ‘hyper’ refer to the level of methylation in the first tissue of the pair compared with the second.

Supplementary Table 12. Information of small RNA data collection and type of samples in this study.

Type of samples	Experiment	Information[a]	Clean data (Gbp)	Sequence depth[b]
smRNA-seq	Illumina	Cone of female individual	0.47	0.07
		Cone of male individual	0.51	0.07
		Leaf of male individual	0.56	0.08
		Central region of the basal meristem from male individual in greenhouse (CM)	0.59	0.08
		Peripheral part of the basal meristem from male individual in greenhouse (PM)	0.80	0.11
		Leaf from male individual in greenhouse (L)	0.54	0.08

a: MM is short for basal meristem from male individual. FM is short for basal meristem from female individual. MY is short for young leaf from male individual. FY is short for young leaf from female individual. CM is short for central region of the basal meristem from male individual in greenhouse. PM is short for peripheral part of the basal meristem from male individual in greenhouse.

b: *G.montanum's* genome size (4.11Gb/1C) and *W. mirabilis's* genome size (7Gb/1C) were estimated from k-mer analysis.

Supplementary Table 13. Statistics of TEs in intergenic region and introns.

	Length (bp)	TEs length (bp)	Percent (%)
Intergenic region	6,166,745,993	5,240,320,179	84.98
Introns	672,574,202	466,045,064	69.29

Supplementary References

1 Crane, P. R. Phylogenetic analysis of seed plants and the origin of angiosperms. *Ann. Mo. Bot. Gard.* **72**, 716-793 (1985).

2 Doyle, J. A. & Donoghue, M. J. Seed plant phylogeny and the origin of angiosperms: an experimental cladistic approach. *Bot. Rev.* **52**, 321-431 (1986).

3 Friedman, W. E. Double fertilization in *Ephedra*, a nonflowering seed plant: its bearing on the origin of angiosperms. *Science* **247**, 951-954 (1990).

4 Carlquist, S. Wood, bark and stem anatomy of new world species of *Gnetum*. *Bot. J. Linn. Soc.* **120**, 1-19 (1996).

5 Doyle, J. A., Donoghue, M. J. & Zimmer, E. A. Integration of morphological and ribosomal RNA data on the origin of angiosperms. *Ann. Mo. Bot. Gard.* **81**, 419-450 (1994).

6 Wickett, N. J. *et al.* Phylotranscriptomic analysis of the origin and early diversification of land plants. *Proc. Natl. Acad. Sci. USA* **111**, E4859 (2014).

7 Leebens-Mack, J. H. *et al.* One thousand plant transcriptomes and the phylogenomics of green plants. *Nature* **574**, 679-685 (2019).

8 Bornman, C., Elsworthy, J. A., ButIer, V. & Botha, C. E. J. *Welwitschia mirabilis*: observations on general habit, seed, seedling, and leaf characteristics. *Madoqua* **1**, 53-66 (1972).

9 Herre, H. The age of *Welwitschia bainesii* (hook. f) carr.: c14 research. *S. Afr. J. Bot.* **27**, 139–140 (1961).

10 Burke, N, J., Seely, M. K. & Jacobsen, K. *Desert.* Pp. 189–214. In: Cowling, R.M., Richardson, D.M. & Pierce, S.M. (eds.), Vegetation of Southern Africa. Cambridge University Press, Cambridge. (1997).

11 Ausin, I. *et al.* DNA methylome of the 20-gigabase Norway spruce genome. *Proc. Natl. Acad. Sci. USA* **113**, E8106-e8113 (2016).

12 Henschel, J. R. & Seely, M. K. Long-term growth patterns of *Welwitschia mirabilis*, a long-lived plant of the Namib Desert (including a bibliography). *Plant Ecol.* **150**, 7-26 (2000).

13 Dubos, C. *et al. MYB* transcription factors in *Arabidopsis*. *Trends Plant Sci.* **15**, 573-581 (2010).

14 Pandey, A., Misra, P. & Trivedi, P. K. Constitutive expression of *Arabidopsis MYB* transcription factor, *AtMYB11*, in tobacco modulates flavonoid biosynthesis in favor of flavonol accumulation. *Plant Cell Rep.* **34**, 1515-1528 (2015).

15 Daneva, A., Gao, Z., Van Durme, M. & Nowack, M. K. Functions and regulation of programmed cell death in plant development. *Annu. Rev. Cell. Dev. Biol.* **32**, 441-468 (2016).

16 Jiang, C. K. & Rao, G. Y. Insights into the diversification and evolution of *R2R3-MYB* transcription factors in plants. *Plant Physiol.* **183**, 637-655 (2020).

17 Stracke, R., Werber, M. & Weisshaar, B. The *R2R3-MYB* gene family in *Arabidopsis thaliana*. *Curr. Opin. Plant Biol.* **4**, 447-456 (2001).

18 Finn, R. D. *et al.* Pfam: the protein families database. *Nucleic Acids Res.* **42**, D222-230 (2014).

19 Edgar, R. C. MUSCLE: multiple sequence alignment with high accuracy and high throughput. *Nucleic Acids Res.* **32**, 1792-1797 (2004).

20 Petroni, K. *et al.* The *AtMYB11* gene from *Arabidopsis* is expressed in meristematic cells and modulates growth in planta and organogenesis *in vitro*. *J. Exp. Bot.* **59**, 1201-1213 (2008).

21 Li, X. *et al.* A genome-wide analysis of the small auxin-up RNA (SAUR) gene family in cotton. *BMC genomics* **18**, 815 (2017).

22 Sun, N. *et al. Arabidopsis SAURs* are critical for differential light regulation of the development of various organs. *Proc. Natl. Acad. Sci. USA* **113**, 6071-6076 (2016).

23 Ren, H. & Gray, W. M. *SAUR* proteins as effectors of hormonal and environmental signals in plant growth. *Mol. Plant* **8**, 1153-1164 (2015).

24 Water *et al.* Evolution, structure and function of the small heat shock proteins in plants. *J. Exp. Bot.* **47**, 325-338 (1996).

25 Schöffl, F. & Key, J. L. An analysis of mRNAs for a group of heat shock proteins of soybean using cloned cDNAs. *J. Mol. Appl. Genet.* **1**, 301-314 (1982).

26 Vierling, E. The roles of heat shock proteins in plants. *Annu. Rev. Plant Physiology Plant Mol. Biol.* **42**, 579-620 (1991).

27 Ouyang, Y., Chen, J., Xie, W., Wang, L. & Zhang, Q. Comprehensive sequence and expression profile analysis of *Hsp20* gene family in rice. *Plant Mol. Biol.* **70**, 341-357 (2009).

28 Nakashima, K., Yamaguchi-Shinozaki, K. & Shinozaki, K. The transcriptional regulatory network in the drought response and its crosstalk in abiotic stress responses including drought, cold, and heat. *Front. Plant Sci.* **5**, 170 (2014).

29 Jiao, Y. *et al.* Ancestral polyploidy in seed plants and angiosperms. *Nature* **473**, 97-100 (2011).

30 Ruprecht, C. *et al.* Revisiting ancestral polyploidy in plants. *Sci. Adv.* **3**, 1603195 (2017).

31 Zwaenepoel, A. & Van de Peer, Y. Inference of ancient whole-genome duplications and the evolution of gene duplication and loss rates. *Mol. Biol. Evol.* **36**, 1384-1404 (2019).
