# Supplementary material for: The Welwitschia genome reveals a unique biology underpinning extreme longevity in deserts
Source: Nat Commun. 2021 Jul 12;12:4247. doi: 10.1038/s41467-021-24528-4 (PMC8275611; doi:10.1038/s41467-021-24528-4)
Supplement: Supplementary file 4 — Description of Additional Supplementary Files [file 41467_2021_24528_MOESM4_ESM.pdf]

Description of additional supplementary files

Title: Supplementary Data 1
Description: The methylation level of CG, CHG and CHH in different type of tissues in *Welwitschia*

Title: Supplementary Data 2
Description: Identification of key gene components involved in RdDM pathway in Welwitschia (including expression profile)

Title: Supplementary Data 3
Description: The expression profile of specific genes investigated in this study

Title: Supplementary Data 4
Description: Gene Ontology enrichment of differentially expressed genes within different tissues

Title: Supplementary Data 5
Description: *Welwitschia* compared with other land plants

Title: Supplementary Data 6
Description: The expression profile of *SAUR* genes

Title: Supplementary Data 7
Description: Measurement of phytohormones in different tissues in *Welwitschia* grown in greenhouse (ng/g)

Title: Supplementary Data 8
Description: The statistics of clean data quality of bisulphite sequencing
