# Supplementary material for: The Welwitschia genome reveals a unique biology underpinning extreme longevity in deserts
Source: Nat Commun. 2021 Jul 12;12:4247. doi: 10.1038/s41467-021-24528-4 (PMC8275611; doi:10.1038/s41467-021-24528-4)
Supplement: Supplementary file 5 — Reporting Summary [file 41467_2021_24528_MOESM5_ESM.pdf]

nature research

Corresponding author(s): Yves Van de Peer
Andrew R. Leitch
Xiao-Quan Wang
Qing-Feng Wang

Last updated by author(s): June 9, 2021

Reporting Summary

Nature Research wishes to improve the reproducibility of the work that we publish. This form provides structure for consistency and transparency in reporting. For further information on Nature Research policies, see our Editorial Policies and the Editorial Policy Checklist.

Statistics

For all statistical analyses, confirm that the following items are present in the figure legend, table legend, main text, or Methods section.

n/a	Confirmed	
☐	☒	The exact sample size (*n*) for each experimental group/condition, given as a discrete number and unit of measurement
☐	☒	A statement on whether measurements were taken from distinct samples or whether the same sample was measured repeatedly
☐	☒	The statistical test(s) used AND whether they are one- or two-sided<br>*Only common tests should be described solely by name; describe more complex techniques in the Methods section.*
☐	☒	A description of all covariates tested
☐	☒	A description of any assumptions or corrections, such as tests of normality and adjustment for multiple comparisons
☐	☒	A full description of the statistical parameters including central tendency (e.g. means) or other basic estimates (e.g. regression coefficient) AND variation (e.g. standard deviation) or associated estimates of uncertainty (e.g. confidence intervals)
☐	☒	For null hypothesis testing, the test statistic (e.g. *F*, *t*, *r*) with confidence intervals, effect sizes, degrees of freedom and *P* value noted<br>*Give P values as exact values whenever suitable.*
☐	☒	For Bayesian analysis, information on the choice of priors and Markov chain Monte Carlo settings
☐	☒	For hierarchical and complex designs, identification of the appropriate level for tests and full reporting of outcomes
☐	☒	Estimates of effect sizes (e.g. Cohen's *d*, Pearson's *r*), indicating how they were calculated

Our web collection on statistics for biologists contains articles on many of the points above.

Software and code

Policy information about availability of computer code

Data collection	The preliminary genome was assembled with WTDBG(version 1.2.8, with parameters -k 0 -p 19 -S 2 -E 2 --rescue-low-cov-edges --aln-noskip);Burrow-Wheeler Aligner (BWA) for short-read alignment (https://github.com/lh3/bwa);Alignments were followed by SNP calling with samtools (https://github.com/samtools). The heterozygosity rate was estimated with bcftools (https://github.com/samtools/bcftools); For bisulphite sequencing, raw reads were first cleaned with SOAPnuke (version 2.0.5); Augustus (version 3.3.1, http://bioinf.uni-greifswald.de/augustus/, with default parameters), SNAP (version 2006-07-28, http://korflab.ucdavis.edu/, with default parameters) and Genscan (version 1.0, http://hollywood.mit.edu/burgelab/software.html, with default parameters) were used for the de novo-based gene prediction.
Data analysis	Paralogous gene pairs found in duplicated collinear and syntenic segments (anchor pairs) from Welwitschia, were detected using i-ADHoRe (version 3.0) with 'level_2_only=TRUE' and 'cluster_type=hybrid'; All initial LTR-RTs detected from LTR-FINDER were blasted against the "Cores Seq" RefSeqdatabase in Gypsy Database 2.0 (GyDB) using blastall (version 2.2.26, ftp://ftp.ncbi.nlm.nih.gov/blast/executables/blast+/, with parameters -m 8 -a 4 -F F -v 500 -b 250 -e 1e-5); The flanking sequences were aligned by MUSCLE (version 3.8.31, http://www.drive5.com/muscle, with default parameters).

For manuscripts utilizing custom algorithms or software that are central to the research but not yet described in published literature, software must be made available to editors and reviewers. We strongly encourage code deposition in a community repository (e.g. GitHub). See the Nature Research guidelines for submitting code & software for further information.

Data

Policy information about availability of data

All manuscripts must include a data availability statement. This statement should provide the following information, where applicable:

- Accession codes, unique identifiers, or web links for publicly available datasets
- A list of figures that have associated raw data
- A description of any restrictions on data availability

The Welwitschia genome project has been deposited at the NCBI under the BioProject number PRJNA680422. The whole genome sequencing data were deposited in the Sequence Read Archive (SRA) database under the accession number SAMN16953877. The Welwtschia and Gnetum assemblies, gene sequences, and annotation data are also available at https://doi.org/10.5061/dryad.ht76hdrdr. The data or related program scripts that support the findings of this study are available from the corresponding author upon request.
The public database used in this study were listed as follow:
1. the library of RepbaseUpdate (20170127); 2. SwissProt protein database (http://www.gpmaw.com/html/swiss-prot.html); 3. the KEGG Automatic Annotation Server (version 2.1, https://www.genome.jp/kaas-bin/kaas_main, with default parameters); 4. RefSeqdatabase in Gypsy Database 2.0 (GyDB); 5. Pfam database version 32.0.

Field-specific reporting

Please select the one below that is the best fit for your research. If you are not sure, read the appropriate sections before making your selection.

☒ Life sciences ☐ Behavioural & social sciences ☐ Ecological, evolutionary & environmental sciences

For a reference copy of the document with all sections, see nature.com/documents/nr-reporting-summary-flat.pdf

Life sciences study design

All studies must disclose on these points even when the disclosure is negative.

Sample size	The sequenced individual Welwitschia were collected from natural wild individuals without any specific selection. The detailed information is offered in "method" of the main text. One male individual was used for whole genome sequencing and cone, leaf, root from the same individual were sampled for RNA-seq which used for annotation; Typical three biological replicates were took for subsequent RNA-seq and methylome sequencing.
Data exclusions	Sequencing data were generated on an Oxford Nanopore GridION, and reads with quality scores of less than 7 were discarded.
Replication	For methylome and transcriptome sequencing, the biological replicates were performed at different days with different research assistants. The results showed good consistency.
Randomization	All the biological samples indeed were belonging to resources in wild population which means covering genetic diversity under natural mutation.
Blinding	The purpose for comparisons were attempt to find varied expression pattern among different tissue types. So all groups with replicates were clarified before analyses.

Reporting for specific materials, systems and methods

We require information from authors about some types of materials, experimental systems and methods used in many studies. Here, indicate whether each material, system or method listed is relevant to your study. If you are not sure if a list item applies to your research, read the appropriate section before selecting a response.

Materials & experimental systems

n/a	Involved in the study	
☒	☐	Antibodies
☒	☐	Eukaryotic cell lines
☒	☐	Palaeontology and archaeology
☒	☐	Animals and other organisms
☒	☐	Human research participants
☒	☐	Clinical data
☒	☐	Dual use research of concern

Methods

n/a	Involved in the study	
☒	☐	ChIP-seq
☒	☐	Flow cytometry
☒	☐	MRI-based neuroimaging
