# Supplementary material for: The Welwitschia genome reveals a unique biology underpinning extreme longevity in deserts
Source: Nat Commun. 2021 Jul 12;12:4247. doi: 10.1038/s41467-021-24528-4 (PMC8275611; doi:10.1038/s41467-021-24528-4)
Supplement: Supplementary file 2 — Review file [file 41467_2021_24528_MOESM2_ESM.pdf]

REVIEWER COMMENTS

Reviewer #1 (Remarks to the Author):

Wan et al. report a chromosome-scale genome assembly of Welwitschia mirabilis, an evolutionary and ecologically important gymnosperm with remarkable biology. They identified changes in genome evolution, repetitive element composition, methylation, and gene expression putatively linked to the unique biology in Welwitschia. I see no technical issues with the genome assembly, annotation, and comparative genomics analyses of Welwitschia and Gnetum. I do have some concerns about the more speculative downstream analyses, and I think addressing these comments will strengthen the manuscript.

1. The patterns of LTR-RT bursts, LTR-RT removal and prevalence of solo LTRs, and differential methylation are consistent with the literature, and illustrate the interesting dynamics of repetitive elements in a large genome. The authors attribute these dynamics to the extreme environment of Welwitschia, which they speculate affects methylation patterns and subsequent TE bursts. Medium-large genomes are characterized by rapid turn over of repetitive elements with half-lives of less than a million years, and this pattern is not unique to Welwitschia or species in extreme environments. The authors present an interesting hypothesis, but I do not see how patterns of genome evolution can be strongly linked to the environment in this case, when these patterns are consistent with other gymnosperms from less extreme habitats.

2. Similarly, the authors claim in line 186 that the high abundance of solo LTRs is linked to selection for small genome sizes in water-stressed environments because this affects stomata size and patterning. This statement is similarly speculative, and the genome size of Welwitschia is enormous, especially compared to Gnetum (which lives in lower stress environments), and most extremophile plants found in Namibia such as succulent CAM plants and desiccation tolerant species. I do not think the paper should be devoid of these speculative claims, but I feel like some are too farfetched. At a minimum, claims like this should be removed from the results section and exclusively mentioned in the discussion.

3. The comparative genomics between Welwitschia and Gnetum are interesting and robust, but I am curious why more comparative analyses were not done between other gymnosperms with high quality genomes such as pine? Ginkgo is included in some analyses, but inclusion of other gymnosperms here, especially with regard to LTR dynamics, would be interesting.

4. Gene family expansions and expression patterns of genes related to environmental stress are highly speculative, and it seems like the authors cherry picked a small percentage of known stress responsive genes as factors controlling stress tolerance in Welwitschia. Below I outline my specific concerns.

Caseinolytic proteases have no differences in expression in the leaf tissue surveyed, and the authors speculate that this reflects protein homeostasis in the face of significant temperature and water stress. I looked at Clp1 expression in Arabidopsis, and it seems to be evenly expressed in all leaf tissues across various expression atlases, similar to what was found in Welwitschia. Clp proteins have a conserved role in all photosynthetic tissue, and I see no evidence that their expression is linked to stress tolerance in Welwitschia.
From the supplemental figure, there is no clear pattern of gene expression for the heat shock proteins. If they are playing a role in stress tolerance, shouldn't they be highly expressed in the older leaves, which are experiencing more severe changes in environment than the protected meristem?
LRR proteins are primarily involved in plant biotic stress, and I see no connection with abiotic stresses here.

The upregulation of a single ABA biosynthesis gene (NCED) is not necessarily linked to stress

tolerance. The ABA regulon is well-characterized, it would be interesting to test if ABA regulated genes are also upregulated in normal leaf conditions compared to other species.

There are some glaring omissions of well-known stress avoidance adaptations. For instance, Welwitschia is a CAM plant and CAM is associated with high water use efficiency and survival in arid environments. No analysis of CAM activity or genes associated with CAM is presented, but this would be one significant and well-supported adaptation linked to survival in hot dry environments.

Minor:
The authors present a nice chromosome-scale genome assembly of Gnetum as well. This is also an important resource for the community, and I think it should be included in the abstract somewhere.

Line 154, were telomeric repeats in Welwitschia identified elsewhere in the pseudomolecules or in the unassembled contigs?

The collinearity analysis between Welwitschia and Gnetum in Figure 2A is difficult to read, and it is almost impossible to see any patterns. I would suggest the authors make a marco-syntenic dot plot using a program such as MCscanx here instead.

Reviewer #2 (Remarks to the Author):

The manuscript represents a very comprehensive study of the Welwitschia genome. Welwitschia is a very unique plant species, isolated within the order of Gnetales, which grows in an extreme dry and hot habitat. The availabilty of the genome sequence in combination with some transcript expression data may allow to understand the biology of this unique species.The manuscript is well written. The authors provide the genome sequence, the DNA methylome of two types of somatic tissues (basal meristem and young leaves), hormone measurements and transcript analyses. All the se experiments appaer to be properly executed. The data show that a large porportion of the genome consists of repetitive elements which are distributed over all chromosomes, and that the genome has undergon substantial rearrangements after Gnetum and Welwitschia diverged. The genome has the capacity for nearly 27 000 protein-coding transcripts , of which the majority has been verified by RNA seq. The levels of cytosine methylation appear to be very high particularly in meristems and leaves.
The manuscript provides useful genomic data which will complement existing genome sequencing data.
The authors try to put their observations in an evolutionary context and speculate about adaptation mechanisms in the discussion. However, this remains hypothetical and no proofs are given.
There are several points which need to be clarified:
One point ist he sampling: were biological repetitions done fort he RNA seq, it does not read like this? However, for the hormone measurements five biological repetitions are mentioned? How much material was available and from which sources? This needs to be clearly described.
What is the basis for the following statement: solo-LTRs, potentially a general response to selection for smaller genome sizes in water-stressed environments, given that small genome sizes are associated with small stomatal pore sizes at high density1 ?
How complete are the transcriptome sequencing data?

Lines 416 to 420 should be moved to introduction
The authors use too often‟ high quality‟, of course the sequencing and assembly data should be of high quality.
The authors should include a very recent publication in the discussion in the manuscript: Jürgens et al. „Welwitschia: Phylogeography of a living fossil‟ Scientific Reports

Reviewer #3 (Remarks to the Author):

This is a well written manuscript based in a large amount of multiomic data. It provides novel information on the genome evolution of a charismatic under-studied plant species. The manuscript discusses the genome evolution of <i>Welwitschia mirabilis</i> in relation to another ancient gymnosperms (<i>Gnetum Montanum</i>, <i>Amborella trichopoda</i>, and <i>Ginkgo biloba</i>). The authors' discuss how genome size reduction by retrotransposon elimination and helped <i>Welwitschia mirabilis</i> adapting to harsh arid environments.

The authors then focus on analyzing the potential contribution of DNA methylation to transposable element silencing and sequence degradation and its possible implications as an adaptation to harsh, nutrient- and water-limited conditions.

Finally, the authors discuss how the duplication, expression, and epigenetic regulation of genes associated with leaf formation, development, and longevity might be another adaptation to environmental stress.

In my view, the article is not easy to read in the current format, with so much supplementary information. However, I understand that this comes with the nature of the extensive work carried out by the authors. I wonder if the authors would be better of splitting the manuscript into multiple sister publications.

Still, I believe this manuscript will attract the interest of a broad audience because of the species studied and the use and integration of multiple datasets. The experimental design and statistical methods used by the authors are strong although the number of individuals studied could have been higher and, certainly, the authors could have applied the same meticulous approach to describe the approaches used for DNAS methylation and gnee expression analysis that they used for the genomic analyses. I cannot comment on the validity of the analysis scripts employed here since they are only available upon request.

There are however some additional comments described below that the authors should address prior manuscript acceptance.

Major comments

It is no apparent to me what sort of library preparation approach the authors used for Nanopore sequencing, i.e., ligation or PCR based Nanopore chemistry. This should be specified since base modification information could be extracted from native DNA sequencing.

Given the high levels of C methylation identified in this study, it would have been important to introduce a control sequence during the bisulfite treatment to determine the efficiency of the C-U conversions of unmethylated Cs. This would remove any concerns of false positives being the reason for the high levels of methylation identified. I understand that there is nothing that can be done at this stage regarding the validation of the efficiency of the original bisulfite treatment but I would suggest that the authors either use Ni et al 2019 DeepSignal on the Nanopore Sequencing data (If libraries were generated using native DNA) or targeted Bisulfite sequencing to validate their WGBS results, both at a global and at a DMR level.

The authors do not report the coverage achieved during WGBS. This is also important in order to be able to call statistically significant DMRs. A low coverage, coupled with the reported high levels of DNA methylation might explain the low variation in CG and CHG methylation observed between tissue

types and between male and female individuals.

Minor comments

Lines 76-78 Out of historical interest, it would be positive to have the references to the mentioned extensive discussions between the Darwin, Gray and Hooker.

Line 154 remove "but"

Lines 209-210 The actual reported global average methylation level for <i>P. abies</i> CHH context 1.5% in <i>in vivo</i> tissue.

Line 415 Missing) at the end of sentence.

In supplemementary information

I believe the statement. "The situation is quite different with previous observation in conifers (84.3% of the total DMRs were in the CHG context between needle and somatic embryogenesis culture cells)" is not quite relevant here since the Ausin et al are comparing <i>in vivo</i> against <i>in vitro</i> cultured somatic embryos and it is well known that somatic embryogenesis significantly alters the methylation status of the cultured samples.

Reviewer #1 (Remarks to the Author):

Wan et al. report a chromosome-scale genome assembly of *Welwitschia mirabilis*, an evolutionary and ecologically important gymnosperm with remarkable biology. They identified changes in genome evolution, repetitive element composition, methylation, and gene expression putatively linked to the unique biology in *Welwitschia*. I see no technical issues with the genome assembly, annotation, and comparative genomics analyses of *Welwitschia* and *Gnetum*. I do have some concerns about the more speculative downstream analyses, and I think addressing these comments will strengthen the manuscript.

> We thank the referee for recognizing the importance of the *Welwitschia* genome. The referee queries some of the analyses and we address the concerns point by point below and revised the text where appropriate.

1. The patterns of LTR-RT bursts, LTR-RT removal and prevalence of solo LTRs, and differential methylation are consistent with the literature, and illustrate the interesting dynamics of repetitive elements in a large genome. The authors attribute these dynamics to the extreme environment of *Welwitschia*, which they speculate affects methylation patterns and subsequent TE bursts. Medium-large genomes are characterized by rapid turnover of repetitive elements with half-lives of less than a million years, and this pattern is not unique to *Welwitschia* or species in extreme environments. The authors present an interesting hypothesis, but I do not see how patterns of genome evolution can be strongly linked to the environment in this case, when these patterns are consistent with other gymnosperms from less extreme habitats.

> The dynamics of the repetitive sequences observed in *Welwitschia* are different compared with (i) those reported for other gymnosperms that have been studied, or (ii) 'medium-large genome' species as we explain in the discussion. Nevertheless, in this section of the manuscript we agree with the referee that we are being overly speculative, and hence the links with aridity have been removed, see next comment.

2. Similarly, the authors claim in line 186 that the high abundance of solo LTRs is linked to selection for small genome sizes in water-stressed environments because this affects stomata size and patterning. This statement is similarly speculative, and the genome size of *Welwitschia* is enormous, especially compared to *Gnetum* (which lives in lower stress environments), and most extremophile plants found in Namibia such as succulent CAM plants and desiccation tolerant species. I do not think the paper should be devoid of these speculative claims, but I feel like some are too farfetched. At a minimum, claims like this should be removed from the results section and exclusively mentioned in the discussion.

> While we could develop this argument further if the editorial board recommended us to do so, we note that the inference would still remain speculative. Thus, as suggested by the referee, we agree that the paper is improved by removing this speculation from the results section; we hope our responses here and above (see (1)) address the referee's concerns.

3. The comparative genomics between *Welwitschia* and *Gnetum* are interesting and robust, but I am curious why more comparative analyses were not done between other gymnosperms with high quality genomes such as pine? Ginkgo is included in some analyses, but inclusion

of other gymnosperms here, especially with regard to LTR dynamics, would be interesting.

We thank the referee for supporting the robustness of our approach, but suggests that adding further comparative analyses with e.g. *Pinus* would be of interest. Our previous work on the *Gnetum* genome (Wan et al., *Nature Plants*. 4, 82-89. 2018) did include comparative analyses between *Gnetum* with *Pinus taeda*. The results were coincident with the pattern observed in Nystedt et al. 2013, showing that most LTR clades in these gymnosperms displayed lineage-specific, deep divergent histories, indicating independent and slow accumulation of ancient repeats independently in each lineage. Here we use the same comparative approach to compare *Gnetum* and *Ginkgo*, and the results are the same (i.e. the presence of deep ancient diverging, species-specific lineages. Yet these results contrast with what we observe for sequences from *Welwitschia*, *Gnetum* and *Amborella*, where deeply diverging clades retain elements from these three species. These data (Fig.2f) and published relationships with *Pinus* (Wan et al. 2018, suppl Fig. 4) are now made clearer in the manuscript (see second paragraph under '*Repetitive sequence dynamics in* Welwitschia').

4. Gene family expansions and expression patterns of genes related to environmental stress are highly speculative, and it seems like the authors cherry picked a small percentage of known stress responsive genes as factors controlling stress tolerance in *Welwitschia*. Below I outline my specific concerns.

We apologize in misleading the referee into thinking that we may have 'cherry picked' our results. Please can we assure the referee and the editorial board that we had not. The genes we analysed come from the comprehensive characterization of specific expanded gene families in *Welwitschia* compared with other representative land plants, as we mention in the Methods section of the manuscript under *'Characterization of specific expanded gene families*' section and shown in the results in Supplementary Fig. 10. From this we identified genes that had expanded in copy number and are known to be involved in stress responses. These included genes for LRR, WRKY, MYB, bHLH, and HSP20-like etc. which were particularly highly expanded in *Welwitschia.* Thus, the expanded families that were also stress related were the focus of a more detailed study. We have now made this clearer in the main manuscript under '*Genes controlling continuous growth*', paragraph 3.

Caseinolytic proteases have no differences in expression in the leaf tissue surveyed, and the authors speculate that this reflects protein homeostasis in the face of significant temperature and water stress. I looked at Clp1 expression in *Arabidopsis*, and it seems to be evenly expressed in all leaf tissues across various expression atlases, similar to what was found in *Welwitschia*. Clp proteins have a conserved role in all photosynthetic tissue, and I see no evidence that their expression is linked to stress tolerance in *Welwitschia*.

We thank the referee for pointing out this and agree that Clp genes play a conserved role in photosynthetic tissue. Nevertheless, we are aware of two publications showing that Clp proteins are more abundant in young leaves compared with older ones, these papers report studies in *Arabidopsis* (Sjögren et al *The Plant Cell*. 18(10): 2635, 2019) and rice (Dong et al. *Plant Physiology*, 162(4):1867, 2013). We had cited both papers in this

context. In the former paper on *Arabidopsis*, in the body of their text they say “With the exception of ClpP6, which showed no significant change, all chloroplast-localized ClpP/R proteins were more abundant in the inner leaves of wild-type plants [i.e. younger] than in the outer leaves. The extent of this increase varied considerably among the different proteins, ranging from only a 25% increase for ClpR2 to a 2.5- to 3-fold increase for ClpP4" (Sjögren et al *The Plant Cell*. 18(10): 2635, 2019). For the later paper on rice, the authors say in the abstract “*VYL* [rice orthologue of Clp] expression is constitutive in most tissues examined but most abundant in leaf sections containing chloroplasts in early stages of development” (Dong et al. *Plant Physiology*, 162(4):1867, 2013). Following the referee’s comment, we looked up the expression of Clp genes in different ages of leaves in *Arabidopsis* in AtGenExpress, data which comes from Schmid et al., *Nature Genetics, 2005*, and compares leaves ranging in age from 7 days to 35 days old. We downloaded the data and using a linear mixed model analysis showed significant differences in gene expression in these tissues, with higher levels in very young leaves (F-value = 3.792, P-value = 0.002). In contrast, in *Welwitschia*, differences were not significantly different between young and old leaf sections (P-value > 0.21). Thus, we feel we are justified in our statements in the submitted manuscript and request that we keep the text as it was.

From the supplemental figure, there is no clear pattern of gene expression for the heat shock proteins. If they are playing a role in stress tolerance, shouldn’t they be highly expressed in the older leaves, which are experiencing more severe changes in environment than the protected meristem?

We observed the specific expansion of genes belonging to the ‘C VI’ subclade of HSP-20 in *Welwitschia*. The tandemly duplicated copies are shown to be highly expressed in meristematic tissue compared to either young or old sections of the leaves. It is likely that the meristematic tissue of *Welwitschia* holds the key to this plant’s extreme longevity, as well as the continuous growth of its elongated leaves. In wild populations (see images below), the main body of the plant can remain healthy even when the leaves are largely destroyed. However, once the meristematic tissue is damaged, the individual soon dies. Thus, it is reasonable to expect a higher expression of HSP in meristems than in leaves, since protecting this tissue from heat or water stress damage is essential. We have added a sentence in the manuscript to highlight this point under the third paragraph of the section ‘*Genes controlling the extreme longevity of leaves and adaptations to environmental stress*’.

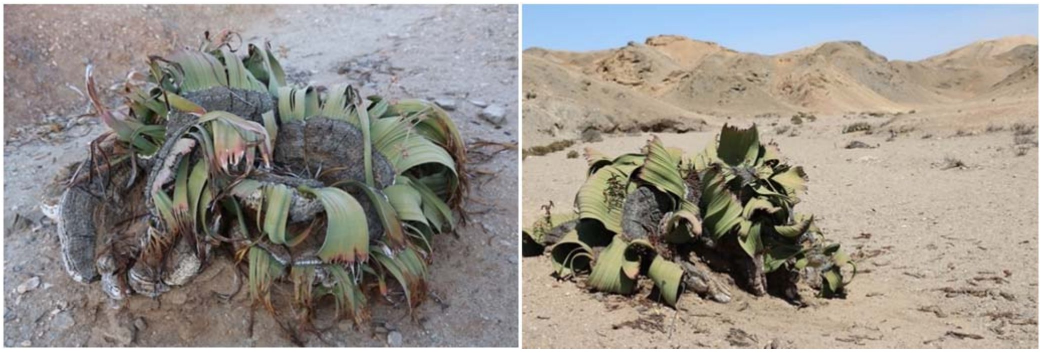

Fig. The damaged leaves of *Welwitschia*, probably chewed by feral donkeys

LRR proteins are primarily involved in plant biotic stress, and I see no connection with abiotic stresses here.

We agree with the referee, thus we have removed abiotic stress from the sentence, which now reads: "For coping with biotic stress, we observed that nucleotide-binding site–leucine-rich repeat (LRR) protein genes which play a role in the biotic stress responses were expanded in copy number in *Welwitschia* compared with other gnetophytes and herbaceous angiosperms that have been analysed[64] (Supplementary Table 10c), and as also observed in other long-lived gymnosperm and angiosperm trees[51,65], indicating further adaptations related to the long lifespan of these plants[51]".

The upregulation of a single ABA biosynthesis gene (NCED) is not necessarily linked to stress tolerance. The ABA regulon is well-characterized, it would be interesting to test if ABA regulated genes are also upregulated in normal leaf conditions compared to other species.

The referee is correct that ABA biosynthesis is not necessarily linked to stress tolerance, although ABA plays a major role in stress tolerance. Because we had identified NCED as a gene family showing *Welwitschia*-specific gene amplification AND because it has a role in stress tolerance, we thought it was worthwhile exploring this gene in more detail (i.e. methylation levels of paralogues, levels of ABA in tissues). We accept the link with stress tolerance is not established without direct experimentation. Nevertheless, in the manuscript we highlighted the study showing that in "lettuce, *Lactuca sativa*, the promoter of *NCED4* is reported to play a role in sensing and responding to heat and is necessary to inhibit seed germination at high temperatures[68]". The gene copies, their methylation and expression levels in *Welwitschia* will provide the tools for future studies.

In response to the referee's comments, we revisited the text (section '*Genes controlling the extreme longevity of leaves and adaptations to environmental stress' paragraph 5)* to make it clear that the link with stress response still needs to be fully demonstrated. We now write "It will be informative to fully demonstrate a link between NCED expression, epigenetic controls and ABA synthesis in protecting *Welwitschia* against heat stress. We expect that ABA functions by limiting growth in the earliest developmental stages of the leaf, but once the fibrous tissues are fully developed, this regulation becomes less

important for the long-term functioning of the leaf and the genes become silenced".

There are some glaring omissions of well-known stress avoidance adaptations. For instance, Welwitschia is a CAM plant and CAM is associated with high water use efficiency and survival in arid environments. No analysis of CAM activity or genes associated with CAM is presented, but this would be one significant and well-supported adaptation linked to survival in hot dry environments.

As we said above, we focused on genes that had expanded in number when selecting targets for detailed analysis, to avoid the 'cherry picking' problem that the referee highlighted above.

Nevertheless, the issue of CAM metabolism is interesting. While *Welwitschia* has indeed been shown to be capable of CAM photosynthesis, a 20-year study found that it makes little use of CAM (von Willert et al., *Functional Plant Biology* 32, 389-395. 2005). During our analyses, we did identify the CAM pathway genes in *Welwitschia*, based on orthologs in pineapple (*Ananas comosus* L. Merr., CAM plant), maize (*Zea mays* L., C4 plant) and rice (*Oryza sativa* L., C3 plant) (see the table below). There was no evidence of any specific increase in the number of copies for any of these gene families in *Welwitschia* when compared with other species analysed, where it has been argued that CAM photosynthesis evolved through the re-organization of paralogous genes to generate new enzyme function (Chen et al., *Horticulture Research.* 7, 5, 2020).

Table. CAM pathway genes in *Welwitschia* and other plants

Gene	*Ananas comosus*	*Phalaenopsis equestris*	*Oryza sativa*	*Zea mays*	*Welwitschia mirabilis*	*Gnetum montanum*	*Ginkgo biloba*
Carbonic anhydrase (CA)	9	15	16	16	10	7	12
Phosphoenolpyruvate carboxylase (PEPC)	3	2	7	6	2	2	3
Phosphoenolpyruvate carboxylase–related kinase	2	2	2	2	3	2	2
Malate dehydrogenase (MDH)	14	5	9	13	5	6	9
Malic enzyme (ME)	5	5	7	8	3	4	3
Phosphoenolpyruvate carboxykinase (PPCK)	1	2	3	6	0	0	2
Pyruvate phosphate dikinase (PPDK)	1	1	2	2	1	1	1
Pyruvate phosphate dikinase regulatory protein	1	1	1	1	1	1	1

Minor:

The authors present a nice chromosome-scale genome assembly of *Gnetum* as well. This is also an important resource for the community, and I think it should be included in the abstract somewhere.

We thank the referee for highlighting the importance of the enhanced *Gnetum* assembly. We realize that we had also failed to highlight the quality of the *Welwitschia* assembly in the abstract, so our new abstract now reads "The *Welwitschia* chromosome level assembly here, along with a new high-quality assembly for *Gnetum montanum*, enhances our understanding of genome evolution in gnetophytes. It also provides critical new insights into the extraordinary development of *Welwitschia*'s ever-growing leaves, enabling its survival in such hostile conditions".

Line 154, were telomeric repeats in *Welwitschia* identified elsewhere in the pseudomolecules or in the unassembled contigs?

With regard to telomeric repeats, we did not characterize them here, although from *in situ* hybridization experiments, we do know that they are of the typical (TTTAGGG)n type found in most plants (unpublished). We are planning another specific study focused on

this specific topic, using approaches specifically designed to distinguish between telomeric motifs at the telomere as opposed to interstitial telomere-like motifs.

The collinearity analysis between *Welwitschia* and *Gnetum* in Figure 2A is difficult to read, and it is almost impossible to see any patterns. I would suggest the authors make a macro-syntenic dot plot using a program such as MCscanx here instead.

We thank the referee for the suggestion. We have made a macro-syntenic dot plot between *Gnetum* and *Welwitschia* and this figure is now added as Supplementary Fig. 17 in Method section ("*Detection of whole genome duplication (WGD) events*' second paragraph) which is cited in the text together with our previous comparison within *Welwitschia*.

With regard to the current Figure 2a, we request that the image is retained here since it also shows the distribution of repetitive sequences along each of the chromosomes.

Reviewer #2 (Remarks to the Author):

The manuscript represents a very comprehensive study of the *Welwitschia* genome. *Welwitschia* is a very unique plant species, isolated within the order of Gnetales, which grows in an extreme dry and hot habitat. The availabilty of the genome sequence in combination with some transcript expression data may allow understanding the biology of this unique species. The manuscript is well written. The authors provide the genome sequence, the DNA methylome of two types of somatic tissues (basal meristem and young leaves), hormone measurements and transcript analyses. All the experiments appear to be properly executed. The data show that a large proportion of the genome consists of repetitive elements which are distributed over all chromosomes, and that the genome has undergone substantial rearrangements after *Gnetum* and *Welwitschia* diverged. The genome has the capacity for nearly 27 000 protein-coding transcripts, of which the majority has been verified by RNA seq. The levels of cytosine methylation appear to be very high particularly in meristems and leaves. The manuscript provides useful genomic data which will complement existing genome sequencing data. The authors try to put their observations in an evolutionary context and speculate about adaptation mechanisms in the discussion. However, this remains hypothetical and no proofs are given. Here are several points which need to be clarified:

We thank the referee for their kind comments regarding the importance of this genome.

One point is he sampling: were biological repetitions done for the RNA seq, it does not read like this? However, for the hormone measurements five biological repetitions are mentioned? How much material was available and from which sources? This needs to be clearly described.

We appreciate the point and have added the descriptive information of the sequenced materials in the new section of Methods called "Plant materials". We have also listed the information in Supplementary Table 6, which clarifies how we sampled material in this study and how many biological replicates were made for RNA seq and methylome seq. As to the hormone measurements, we have mentioned the number of replicates in the

appropriate section in Methods '*Measurement of phytohormones and determination of endogenous Brassinosteroid (BR) levels*'. We have also enhanced the information on the plant material studied, which now includes plant accession number and locality details. We hope these modifications address the referee's concerns.

What is the basis for the following statement: solo-LTRs, potentially a general response to selection for smaller genome sizes in water-stressed environments, given that small genome sizes are associated with small stomatal pore sizes at high density?

We agree with the referee that this was not clear, and the point is not important enough to use up a lot of space in its explanation, so we have removed this from the results section.

How complete are the transcriptome sequencing data? Lines 416 to 420 should be moved to introduction. The authors use too often "high quality", of course the sequencing and assembly data should be of high quality.

We thank the referee for pointing this out. We have now added the requested information to Supplementary Table 6 which includes an assessment of the transcriptome seq data. The use of "high quality" is a reference to the quality of the genome assembly generated by us, or in other papers and it is now mentioned only twice.

The authors should include a very recent publication in the discussion in the manuscript: Jürgens et al. *Welwitschia*: Phylogeography of a living fossil "Scientific Reports

We thank the referee for alerting us to this publication and the reference has now been added to the introduction. Furthermore, we also contacted Prof. Norbert Jürgens who kindly provided us with the latest distribution records of *Welwitschia*'s, thus we have updated Figure 1.

Reviewer #3 (Remarks to the Author):

This is a well written manuscript based in a large amount of multiomic data. It provides novel information on the genome evolution of a charismatic under-studied plant species. The manuscript discusses the genome evolution of *Welwitschia mirabilis* in relation to another ancient gymnosperms (*Gnetum Montanum*, *Amborella trichopoda*, and *Ginkgo biloba*). The authors discuss how genome size reduction by retrotransposon elimination and helped *Welwitschia mirabilis* adapting to harsh arid environments.
The authors then focus on analyzing the potential contribution of DNA methylation to transposable element silencing and sequence degradation and its possible implications as an adaptation to harsh, nutrient- and water-limited conditions.
Finally, the authors discuss how the duplication, expression, and epigenetic regulation of genes associated with leaf formation, development, and longevity might be another adaptation to environmental stress.

We thank the referee for his/her generous comments and overview of the manuscript.

In my view, the article is not easy to read in the current format, with so much supplementary information. However, I understand that this comes with the nature of the extensive work

carried out by the authors. I wonder if the authors would be better of splitting the manuscript into multiple sister publications.

We thank the referee for this suggestion. As the referee kindly acknowledges, we have an enormous amount of information on this genome. The data presented support and reinforce each other and we hope that the key findings we have extracted and presented generates a compelling paper suitable for Nature Communications. We think that it is only by analysing and presenting all these different components into a combined analysis that deep insights into *Welwitschia*'s longevity and morphology fully emerges. The style is established in the literature for major genome initiatives like this one, so we hope the referee and editorial board are content that we keep the current structure of the manuscript intact.

Still, I believe this manuscript will attract the interest of a broad audience because of the species studied and the use and integration of multiple datasets. The experimental design and statistical methods used by the authors are strong although the number of individuals studied could have been higher and, certainly, the authors could have applied the same meticulous approach to describe the approaches used for DNAs methylation and gene expression analysis that they used for the genomic analyses. I cannot comment on the validity of the analysis scripts employed here since they are only available upon request.

We thank the referee for acknowledging the broad interest of our work to a diverse audience.

There are however some additional comments described below that the authors should address prior manuscript acceptance.

Major comments

It is no apparent to me what sort of library preparation approach the authors used for Nanopore sequencing, i.e., ligation or PCR based Nanopore chemistry. This should be specified since base modification information could be extracted from native DNA sequencing.

We thank the referee for pointing this out and we have now specified the library preparation approach in the Methods section under section '*De novo* sequencing and genome assembly' we write: "*After extracting high quality DNA, the large size fraction was selected by automated gel electrophoresis (BluePippin). Then, the DNA was treated with the End-repair/dA tailing module. After purification, adapter ligation was performed using ligation sequencing kit (LSK109, Oxford Nanopore Technologies). Finally, DNA library was quantified by Qubit.*"

Given the high levels of C methylation identified in this study, it would have been important to introduce a control sequence during the bisulfite treatment to determine the efficiency of the C-U conversions of unmethylated Cs. This would remove any concerns of false positives being the reason for the high levels of methylation identified. I understand that there is nothing that can be done at this stage regarding the validation of the efficiency of the original bisulfite treatment but I would suggest that the authors either use Ni et al 2019 DeepSignal on the Nanopore Sequencing data (If libraries were generated using native DNA) or targeted

Bisulfite sequencing to validate their WGBS results, both at a global and at a DMR level.

We thank the referee for pointing out this. We did in fact use unmethylated lambda phage DNA to control the efficiency of bisulfite conversion in the BS-seq study. The conversion rate ranged from 99.44% to 99.48% across all samples. These data are now provided in Supplementary Table 7b.

In addition, we have now specified the details of the method used in the section 'Methylome and transcriptome sequencing' where we write "*Bisulfite conversion was conducted on 1 µg fragmented DNA using the EZ DNA Methylation-Gold Kit (Zymo Research, Cat. No. D5005). A total of 5 ng of lambda phage DNA was added to determine the efficiency of C-U conversion of unmethylated Cs*".

The authors do not report the coverage achieved during WGBS. This is also important in order to be able to call statistically significant DMRs. A low coverage, coupled with the reported high levels of DNA methylation might explain the low variation in CG and CHG methylation observed between tissue types and between male and female individuals.

We thank the referee for pointing this out. The average sequencing depth was ~14.5x and information on read coverage for each sample is now provided in Supplementary Table 6 while information on data quality is given in Supplementary Table 7b. We believe our sequencing depth is acceptable given that each sample has three biological replicates (Ziller et al., *Nature Methods*. 12(3): 230-232, 2015).

Minor comments

Lines 76-78 Out of historical interest, it would be positive to have the references to the mentioned extensive discussions between the Darwin, Gray and Hooker.

The reference (Friedman, *American Journal of Botany,* 102, 312–324. 2015) has now been cited in the revised manuscript.

Line 154 remove "but"

Corrected

Lines 209-210 The actual reported global average methylation level for *P. abies* CHH context 1.5% in *in vivo* tissue.

We thank the reviewer for the correction and the number has now been changed.

Line 415 Missing) at the end of sentence.

Corrected

In supplementary information

I believe the statement. "The situation is quite different with previous observation in conifers (84.3% of the total DMRs were in the CHG context between needle and somatic embryogenesis culture cells)" is not quite relevant here since the Ausin et al are comparing *in*

vivo against *in vitro* cultured somatic embryos and it is well known that somatic embryogenesis significantly alters the methylation status of the cultured samples.

We agree with the referee on this and have removed the statement.

REVIEWERS' COMMENTS

Reviewer #1 (Remarks to the Author):

The authors have addressed my previous comments and concerns in their revision.

Reviewer #2 (Remarks to the Author):

The authors have tried to address most points of the reviewers. Nevertheless I noted some points which should be revised:

„Reduced GC content will confer a genomic advantage under nutrient limitation‟ this statement should be removed evidence is not really available .
Line 194 of ongoing removal: replace removal by reduction
Line 198: wild sourced material replace by material collected in the wild (give also place of collection)
Line 274: better say: are impossible to be killed
Line 369: „once the meristematic tissue is damaged, the individual soon dies‟ this is not specific to Welwitschia, this is true for probably any plant, so avoid overinterpretation
Line 459: young section of leaf of male individuals: how ia a young section defined?
Line 470: omit high quality and replace by high molecular weight (indicate size range)
Line 472: Then, the DNA was treated with the End-repair/dA tailing module (supplier?)

Reviewer #3 (Remarks to the Author):

I recommend this article to be accepted for publication

REVIEWERS' COMMENTS

Reviewer #1 (Remarks to the Author):

The authors have addressed my previous comments and concerns in their revision.

Reviewer #2 (Remarks to the Author):

The authors have tried to address most points of the reviewers. Nevertheless I noted some points which should be revised:

"Reduced GC content will confer a genomic advantage under nutrient limitation", this statement should be removed evidence is not really available .
We thank referee and agree that we state this too strongly, but it is known that GC dinucleotides are more N demanding than AT dinucleotides. In shortening the abstract this section has now been removed.

Line 194 of ongoing removal: replace removal by reduction
Corrected

Line 198: wild sourced material replace by material collected in the wild (give also place of collection)
Corrected. The place of collection is given in the 'Plant Materials' section of the methods in main text where we write, 'collected in 'Welwitschia Wash' (S23.6124; E 15.1696), in the Namib-Naukluft Park and from the northern bank of the Kuiseb River'.

Line 274: better say: are impossible to be killed
We actually have used a play on the translated quote 'two leaves that cannot die'

Line 369: "once the meristematic tissue is damaged, the individual soon dies" this is not specific to Welwitschia, this is true for probably any plant, so avoid overinterpretation
The referee is right. However, for this point we are only trying to explain the higher expression of *HSP-20* copies in meristem than the leaves.
Line 459: young section of leaf of male individuals: how is a young section defined?
The young section of leaves we sampled are very close to the terminal grooves as we marked in Figure 4c. This is now referenced in 'Plant Materials' of the 'Methods' section.

Line 470: omit high quality and replace by high molecular weight (indicate size range)
Corrected and size added under section 'De novo sequencing and genome assembly'.

Line 472: Then, the DNA was treated with the End-repair/dA tailing module (supplier?)
The information had been added as "*the DNA was treated with the End-repair/dA tailing module (New England Biolabs, Inc.)*"
Reviewer #3 (Remarks to the Author):

I recommend this article to be accepted for publication
